# Topology surveillance of the lanosterol demethylase CYP51A1 by signal peptide peptidase

**DOI:** 10.1242/jcs.262333

**Published:** 2024-12-12

**Authors:** Nikita Sergejevs, Dönem Avci, Michael L. van de Weijer, Robin A. Corey, Marius K. Lemberg, Pedro Carvalho

**Affiliations:** ^1^ Sir William Dunn School of Pathology, University of Oxford, South Parks Road, Oxford OX1 3RE, UK; ^2^ Center for Biochemistry and Cologne Excellence Cluster on Cellular Stress Responses in Aging-Associated Diseases (CECAD), Medical Faculty, University of Cologne, 50931 Cologne, Germany; ^3^ School of Physiology, Pharmacology & Neuroscience, University of Bristol, Biomedical Sciences Building, University Walk, Bristol BS8 1TD, UK

**Keywords:** Intramembrane proteolysis, Endoplasmic reticulum-associated protein degradation, ERAD, Ubiquitin ligase, Endoplasmic reticulum, Protein quality control, Signal peptide peptidase, SPP

## Abstract

Cleavage of transmembrane segments on target proteins by the aspartyl intramembrane protease signal peptide peptidase (SPP, encoded by *HM13*) has been linked to immunity, viral infection and protein quality control. How SPP recognizes its various substrates and specifies their fate remains elusive. Here, we identify the lanosterol demethylase CYP51A1 as an SPP substrate and show that SPP-catalysed cleavage triggers CYP51A1 clearance by endoplasmic reticulum-associated degradation (ERAD). We observe that SPP targets only a fraction of CYP51A1 molecules, and we identify an amphipathic helix in the CYP51A1 N terminus as a key determinant for SPP recognition. SPP recognition is remarkably specific to CYP51A1 molecules with the amphipathic helix aberrantly inserted in the membrane with a type II orientation. Thus, our data are consistent with a role for SPP in topology surveillance, triggering the clearance of certain potentially non-functional conformers.

## Teaser

**Highlighted Article:** We show that the intramembrane protease SPP conducts topology surveillance of CYP51A1 by initiating quality control of incorrectly inserted molecules.

## INTRODUCTION

In eukaryotic cells, the biogenesis of integral membrane and secretory proteins occurs at the endoplasmic reticulum (ER). The ER is the largest membrane-bound organelle, and it possesses a myriad of factors to facilitate maturation, folding and post-translational modifications of target proteins (Helenius and Aebi, 2004; Braakman and Hebert, 2013). These processes are prone to failure, and a significant fraction of newly synthesized polypeptides end up misfolded. Accumulation of these species is toxic, compromising ER protein homeostasis and cell physiology (Hartl and Hayer-Hartl, 2009). Indeed, protein homeostasis defects are a common hallmark of pathologies, including cystic fibrosis, Parkinson's disease and Alzheimer's disease (Dubnikov et al., 2017; Bhattacharya and Qi, 2019; Needham et al., 2019). By detecting and eliminating misfolded and other potentially toxic species, quality control systems, such as ER-associated degradation (ERAD), play a central role in protein homeostasis. The ERAD process promotes the recognition, ER extraction and ubiquitylation of aberrant proteins to facilitate their degradation by cytosolic proteasomes (Christianson and Ye, 2014; Wu and Rapoport, 2018; Mehrtash and Hochstrasser, 2019). Even though ERAD is primarily linked to degradation of misfolded and unassembled proteins, it also targets specific functional proteins in a regulated fashion (Ruggiano et al., 2016; Matsumoto et al., 2019; Natarajan et al., 2020; McKenna et al., 2022; Yagita et al., 2023). In general, ERAD consists of multiple branches, each defined by a membrane-embedded E3 ubiquitin ligase complex with specificity towards distinct substrates (Krshnan et al., 2022a). The various branches all converge on the cytosolic p97 ATPase (also known as VCP), which facilitates the extraction of ubiquitylated substrates from the ER membrane and their delivery to the proteasome for degradation (Christianson and Carvalho, 2022). To date, mechanisms of membrane substrate recognition, as well as the role of auxiliary factors such as intramembrane proteases in this process, remain ill defined.

Intramembrane proteolysis is an irreversible enzymatic reaction that provides an attractive mechanism for dynamic regulation of the membrane proteome (Kopan and Ilagan, 2004; Kühnle et al., 2019). In general, intramembrane proteases cleave peptide bonds within the plane of the cellular membranes – a hydrophobic environment that is rarely exposed to water (Sun et al., 2016). So far, several ER-resident intramembrane proteases have been shown to be involved in a non-canonical proteolytic arm of protein quality control, by functionally interacting with ERAD machinery to target substrates for degradation (Kühnle et al., 2019). A member of the aspartyl intramembrane protease family, signal peptide peptidase (SPP, encoded by *HM13*), has been found to interact with E3 ubiquitin ligases, such as TRC8 (also known as RNF139) and MARCHF6, to selectively target tail-anchored (TA) membrane proteins for degradation by ERAD (Boname et al., 2014; Chen et al., 2014; Stefanovic-Barrett et al., 2018; Avci et al., 2019).

SPP has been found to be essential for surveillance of the major histocompatibility complex class I (MHC-I) molecules HLA-A, HLA-B and HLA-C, with cleaved MHC-I signal peptides loaded onto the non-classical MHC-I molecule HLA-E for presentation at the cell surface and self-recognition by natural killer cells (Lemberg et al., 2001; Bland et al., 2003). Recently, SPP has also been found to more globally regulate the peptide repertoire presented at the cell surface, with SPP knockout (KO) cells displaying fewer surface MHC-I molecules than wild-type (WT) cells (Bruno et al., 2023).

In addition, SPP has been found to play a role in virus propagation mechanisms by participating in production of the mature core protein of hepatitis C virus (HCV), with inhibition of the intramembrane protease activity leading to degradation of immature HCV core protein by the ubiquitin–proteasome system and a subsequent decrease in the abundance of infectious viral particles (McLauchlan et al., 2002; Aizawa et al., 2016).

SPP is highly expressed in different tumour cells, with SPP overexpression correlating with poor prognosis in human lung and breast cancers (Hsu et al., 2019). Depletion of SPP results in inhibition of tumour proliferation and migration both *in vitro* and *in vivo* (Hsu et al., 2019; Liu et al., 2022), at least partially through impaired cleavage of the TA membrane protein heme oxygenase 1 (HO-1) and the type II membrane protein FKBP8 (Hsu et al., 2015).

Furthermore, SPP has been implicated in the recognition of aberrant polytopic membrane proteins, such as truncated versions of bovine rhodopsin (Crawshaw et al., 2004). Moreover, recent evidence suggests a role of SPP in cleavage of mislocalized mitochondrial TA proteins for their subsequent degradation by ERAD when the ER-resident dislocase ATP13A1 is absent (McKenna et al., 2022). An SPP homologue in yeast, Ypf1, has been shown to interact with the E3 ubiquitin ligase Doa10 (also known as Ssm4) and the rhomboid pseudoprotease Dfm1 to regulate the abundance of high-affinity zinc transporters as an adaptive response to nutrient depletion (Avci et al., 2014). With a number of reports providing evidence on the alternative role of SPP in membrane protein quality control, how the intramembrane protease cooperates with the E3 ubiquitin ligase complex for substrate delivery for subsequent downstream processes is still unknown.

A recent genome-wide CRISPR/Cas9 screen for ERAD components implicated in the clearance of a model misfolded membrane protein, CYP51A1TM, identified a novel ERAD branch involving an RNF185–membralin (MBRL) complex (van de Weijer et al., 2020). Curiously, SPP was also detected among the top hits of this genetic screen. Thus far, the mechanism of SPP interaction with components of the ERAD machinery, specifically the features recognized on the substrate and sequence of events, has remained enigmatic. In this study, we identify SPP as a protein quality control factor involved in initiating the clearance of a population of lanosterol demethylase CYP51A1 molecules. Using biochemical and genetic approaches, we show that CYP51A1 contains a metastable N-terminal amphipathic helix (AH) that is recognized by SPP when aberrantly inserted in the ER membrane in a type II orientation, leading to its subsequent degradation via the RNF185–MBRL ERAD complex. These findings describe a previously unrecognized function of SPP in protein topology surveillance.

## RESULTS

### SPP is required for CYP51A1 degradation

**Fig. 1.**
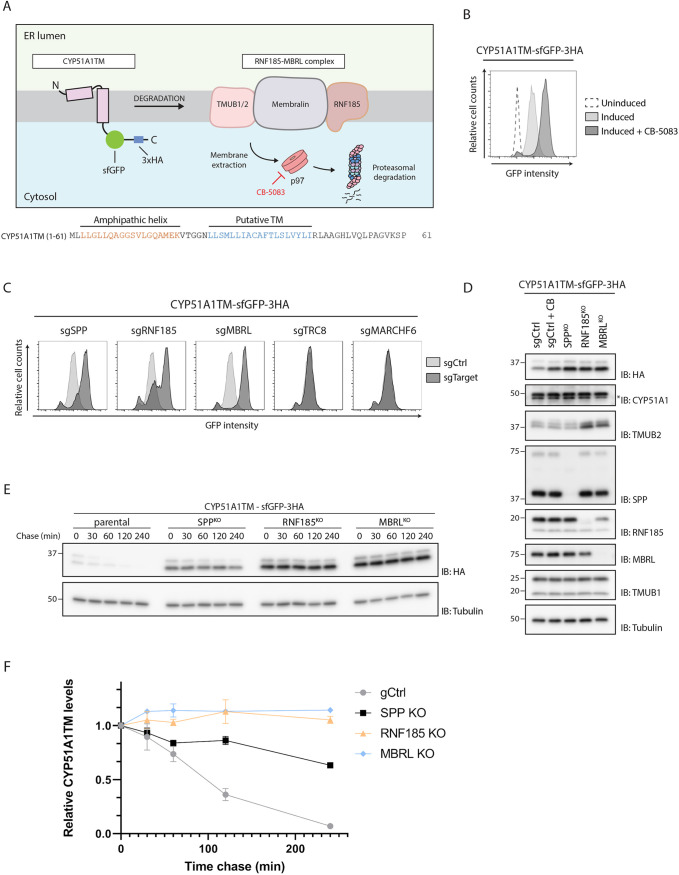
**SPP is required for CYP51A1 and CYP51A1TM degradation.** (A) Schematic overview of CYP51A1TM degradation by the RNF185–MBRL E3 ubiquitin ligase complex. The model substrate consists of the first 61 amino acids of lanosterol demethylase, CYP51A1, encompassing an N-terminal AH followed by a single TM domain and is expressed as a fusion to sfGFP and 3×HA tags. The p97 ATPase is inhibited by the specific inhibitor CB-5083. Protein sequences of the AH and predicted TM domain are highlighted below the schematic in orange and blue, respectively. (B) Flow cytometry analysis (based on GFP fluorescence) of doxycycline-induced expression of CYP51A1TM in HEK293 T-Rex Flip In cells. Analysis was performed 16 h after the time of doxycycline induction in cells that were uninduced (dotted line), induced (light grey), or induced and incubated for 4 h with p97 inhibitor (dark grey; CB-5083, 2.5 µM). (C,D) Flow cytometry (C) and immunoblotting (D) analysis of doxycycline-induced expression (16 h) of CYP51A1TM in HEK293 T-Rex Flip In cells either treated for 4 h with p97 inhibitor CB-5083 (CB) or transfected with plasmids encoding gRNAs (sg) targeting the indicated genes. In C, fluorescence intensity is compared between cells transfected with a control plasmid (sgCtrl, light grey) versus cells transfected with a target gRNA (sgTarget, dark grey). In D, asterisk represents non-specific band on CYP51A1 immunoblot. Molecular masses are indicated in kDa. Data in B–D are representative of three independent experiments. (E,F) Turnover of CYP51A1TM was analysed upon treatment with cycloheximide over a 4 h time course to block new protein translation in parental HEK293 T-Rex Flip In cells transfected with a control plasmid (gCtrl in F) and in SPP, RNF185 and MBRL KO cells. (E) Cells were harvested at the indicated time points and analysed by SDS-PAGE and immunoblotting. Molecular masses are indicated in kDa. (F) Quantification of the immunoblots, presented as the mean of three independent biological replicates (*n*=3); error bars represent s.d. IB, immunoblot.

A previous genome-wide CRISPR-Cas9 genetic screen identified an ERAD complex consisting of the multi-spanning membrane protein MBRL, the ubiquitin ligase RNF185, and a member of the transmembrane and ubiquitin-like family (TMUB) – either TMUB1 or TMUB2. This RNF185–MBRL complex was found to be essential for the turnover of the lanosterol demethylase CYP51A1 as well as CYP51A1TM, a derived model substrate consisting of the first 61 amino acids of CYP51A1 (van de Weijer et al., 2020), encompassing the N-terminal AH followed by a single transmembrane (TM) domain (Fig. 1A). The same genetic screen also identified the aspartyl intramembrane protease SPP as an essential factor for the degradation of CYP51A1TM (van de Weijer et al., 2020). We expressed the CYP51A1TM model substrate as a fusion to superfolder GFP (sfGFP) and HA tags (CYP51A1TM–sfGFP–3HA), to facilitate detection by flow cytometry and immunoblotting, from a doxycycline-inducible promoter in HEK293 T-Rex Flip In cells. Consistent with our previous observations (van de Weijer et al., 2020), we observed that the steady-state levels of CYP51A1TM increased upon inhibition of the p97 ATPase with CB-5083, a potent and highly selective inhibitor of the D2 ATPase domain of p97 that blocks ERAD substrate extraction from the membrane (Zhou et al., 2015; Tang et al., 2019) (Fig. 1A,B). Similarly, depletion of SPP or components of the RNF185–MBRL ERAD complex also resulted in higher CYP51A1TM steady-state levels (Fig. 1C,D). In contrast, depletion of other ERAD factors, such as the ubiquitin ligases TRC8 or MARCHF6 previously shown to cooperate with SPP on the degradation of TA proteins, had no effect (Fig. 1C) (Boname et al., 2014; Stefanovic-Barrett et al., 2018; Avci et al., 2024). Importantly, endogenous CYP51A1 was found to be regulated in a similar fashion, as depletion of SPP, RNF185 and MBRL also resulted in an increase in its steady-state levels (Fig. 1D). The increase in CYP51A1 levels upon SPP depletion was reproducible across biological replicates and was observed in multiple SPP-depleted clones of HEK293 T-Rex Flip In and HeLa cell lines (Fig. S1A,B). Depletion of SPP did not affect the steady-state levels of TMUB2, another substrate of the RNF185–MBRL complex (Fig. 1D), indicating that its effect is specific to CYP51A1 and that SPP does not interfere with the general function of the RNF185–MBRL complex.

To test whether the increased CYP51A1TM steady-state levels resulted from a defect in protein degradation, we performed cycloheximide chase experiments (Fig. 1E,F). In control HEK293 T-Rex Flip In cells, CYP51A1TM was a short-lived protein with a half-life of around 90 min, whereas depletion of RNF185 or MBRL blocked degradation of CYP51A1TM, consistent with previous reports (van de Weijer et al., 2020). Similarly, the half-life of CYP51A1TM was substantially extended in cells lacking SPP but to a lower extent than the effect of loss of RNF185 or MBRL (Fig. 1E,F). Thus, SPP plays an important role in degradation of CYP51A1TM.

### CYP51A1 and CYP51A1TM are direct substrates of SPP

**Fig. 2.**
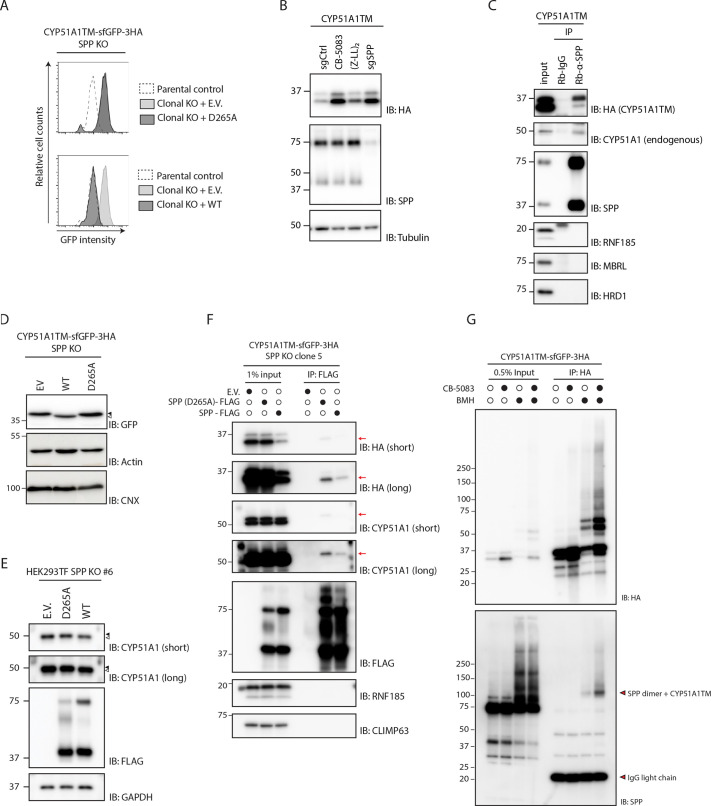
**SPP directly binds to CYP51A1 and CYP51A1TM, and its catalytic activity is required for ERAD.** (A) Model substrate levels were assessed by flow cytometry in clonal SPP KO HEK293 T-Rex Flip In cells expressing cDNA encoding either the empty vector (E.V., light grey), a catalytically inactive SPP mutant (D265A; dark grey, top panel) or WT SPP (dark grey, bottom panel). Fluorescence intensity was compared to that of WT control cells (dotted line). (B) Immunoblot (IB) analysis of HEK293 T-Rex Flip In cells expressing CYP51A1TM treated with a control gRNA (sgCtrl), compared to treatments with p97 inhibitor (CB-5083, 4 h) or SPP-specific inhibitor [(Z-LL)_2_-ketone, 16 h]. SPP KO HEK293 T-Rex Flip In cells (sgSPP) were used as a positive control of substrate stabilization levels. (C) Proteins co-precipitating with endogenous SPP. HEK293 T-Rex Flip In cells expressing CYP51A1TM were lysed in 1% DMNG lysis buffer, after which endogenous SPP was immunoprecipitated (IP) using Protein-G beads conjugated to SPP antibody (Rb-α-SPP). Proteins were eluted and subjected to SDS-PAGE followed by immunoblotting for the proteins indicated. Rabbit IgG (Rb-IgG) was used as a negative control. Input lane was loaded with 1% of total lysate. (D) Immunoblot analysis of CYP51A1TM levels in SPP KO HEK293 T-Rex Flip In cells upon expression of empty vector (EV), or of WT or catalytically inactive (D265A) mutants of SPP. Dark arrowhead shows unprocessed version of CYP51A1TM, whereas white arrowhead shows the faster-migrating, SPP-processed version of the substrate. Actin and calnexin are used as loading controls. (E) Immunoblot analysis of endogenous CYP51A1 levels in SPP KO HEK293 T-Rex Flip In cells upon expression of empty vector (E.V.), or of WT or catalytically inactive (D265A) mutants of SPP. The lysates were analysed by loading onto 10% acrylamide gels using a Tris-Tricine buffering system. Dark arrowhead shows the unprocessed version of CYP51A1TM, whereas the white arrowhead shows the faster-migrating, SPP-processed version of the substrate. Short and long exposures of the CYP51A1 blot are shown. (F) Proteins co-precipitating with ectopic WT SPP and catalytically inactive (D265A) SPP mutant. Clonal SPP KO HEK293 T-Rex Flip In cells expressing the model substrate were transduced either with empty vector (E.V.), or with catalytically inactive SPP (D265A) or WT SPP protease appended to a C-terminal 3×FLAG tag. Cells expressing the target constructs were lysed in 1% DMNG buffer, after which proteins co-precipitating with ectopically expressed FLAG-tagged SPP were analysed by SDS-PAGE and immunoblotting. Red arrows indicate either the transgene-specific band (for anti-HA blots) or endogenous protein-specific band (for anti-CYP51A1 blots). Short and long exposures of the HA and CYP51A1 blots are shown. (G) BMH crosslinking reactions in HEK293 T-Rex Flip In cells expressing CYP51A1TM performed in the presence or absence of p97 inhibitor CB-5083 (2.5 µM, 4 h). Crosslinked lysates after quenching were subjected to immunoprecipitation using anti-HA beads, and eluted proteins were subjected to SDS-PAGE followed by immunoblotting for the indicated proteins. Samples were analysed directly (Input) or after denaturing immunoprecipitation with anti-HA antibody. The upper red arrowhead indicates direct crosslink adducts between endogenous SPP and CYP51A1TM. The lower red arrowhead marks IgG light chain. In B–G, molecular masses are indicated in kDa. Data in A–G are representative of three (*n*=3) independent experiments.

The proteolytic activity of SPP requires catalytic aspartate residues in TM domain 6 (YD motif) and TM domain 7 (GxGD motif, where x indicates any amino acid), and an SPP D265A mutant has previously been shown to be catalytically inactive (Weihofen et al., 2002). To test the role of SPP catalytic activity in CYP51A1TM degradation we re-expressed either WT SPP or the D265A catalytically dead mutant in SPP clonal KO cells. Expression of WT SPP restored CYP51A1TM degradation, as observed by the low steady-state levels, whereas the SPP D256A mutant failed to do so (Fig. 2A). Consistent with a requirement for SPP catalytic activity, treatment of cells with the SPP-specific inhibitor (Z-LL)_2_-ketone increased the steady-state level of both endogenous CYP51A1 (Fig. S1C) and CYP51A1TM (Fig. 2B; Fig. S1D). These results suggest that CYP51A1TM and CYP51A1 are substrates of SPP. To further explore this possibility, we used immunoprecipitation to test whether SPP interacts with these proteins. We observed that endogenous SPP interacts with both CYP51A1TM and endogenous CYP51A1 (Fig. 2C).

We next asked whether CYP51A1TM is a direct substrate for SPP. Since proteolytic ERAD intermediates generated by endogenous SPP are commonly difficult to detect (Boname et al., 2014; Chen et al., 2014; Avci et al., 2019), we used an overexpression system and a high-resolution gel allowing the detection of small changes in molecular mass. Ectopic expression of WT SPP, but not of catalytically inactive D265A mutant, in SPP KO cells resulted in a faster migrating form of both CYP51A1TM (Fig. 2D) and endogenous CYP51A1 (Fig. 2E), likely due to N-terminal trimming. Consistent with such an enzyme–substrate interaction, the catalytically inactive D265A mutant precipitated higher amounts of both CYP51A1TM and endogenous CYP51A1 (Fig. 2F), when compared to the WT SPP, showing a substrate trapping effect, as has been observed previously (Schrul et al., 2010; Chen et al., 2014). In all these experiments the interactions were specific, given that other abundant ER membrane proteins did not interact with SPP (Fig. 2C,F). Therefore, SPP binds to CYP51A1TM and CYP51A1 independently of its proteolytic activity, but this activity is required to promote turnover of an N-terminally trimmed version of the substrate via the ERAD machinery.

**Fig. 3.**
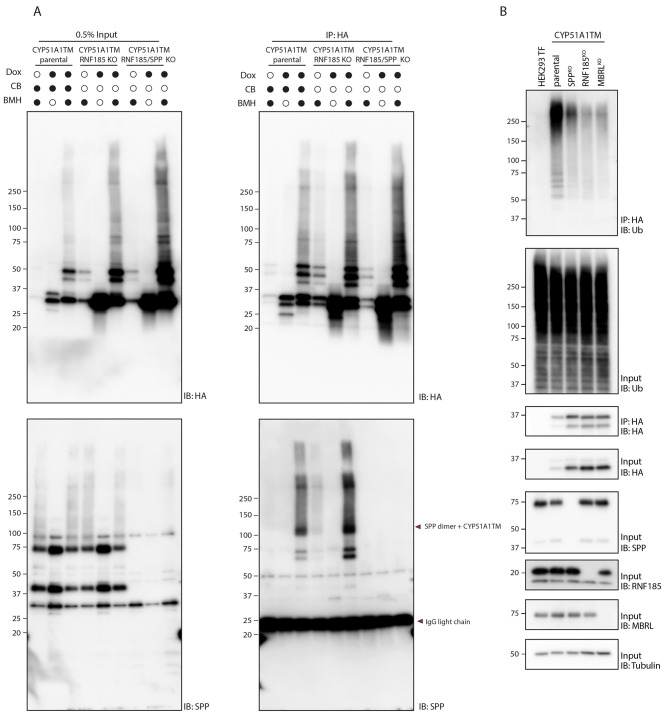
**SPP acts upstream of the RNF185–MBRL E3 ubiquitin ligase complex.** (A) Assessment of a direct SPP binding using BMH crosslinker. Similar to Fig. 2G, BMH crosslinking reactions were performed in parental HEK293 T-Rex Flip In cells, as well as in HEK293 T-Rex Flip In cells depleted for either E3 ubiquitin ligase RNF185 (RNF185 KO) or both SPP and RNF185 (RNF185/SPP KO). Expression of CYP51A1TM was induced by treatment with doxycycline (Dox), as indicated. Samples were analysed directly (Input) or after denaturing immunoprecipitation (IP) with anti-HA antibody by immunoblotting (IB) for the indicated proteins. Red arrowheads represent direct crosslink adducts between endogenous SPP and CYP51A1TM (upper arrowhead) and IgG light chain (lower arrowhead). CB, CB-5083 treatment. (B) Assessment of the ubiquitylation status of CYP51A1TM. The substrate was immunoprecipitated from HEK293 T-Rex Flip In cells expressing CYP51A1TM using anti-HA magnetic beads, and ubiquitylation levels were compared between parental cells and cells lacking the genes indicated. Parental HEK293 T-Rex Flip In cells lacking CYP51A1TM–sfGFP–3×HA (HEK293 TF) were used as a negative control. In A and B, molecular masses are indicated in kDa. Data are representative of three independent experiments.

Next, we used the cysteine-reactive crosslinker bismaleimidohexane (BMH) to test whether SPP binds CYP51A1TM directly (Crawshaw et al., 2004). This was a suitable approach given that CYP51A1TM–sfGFP–3HA has four cysteine residues (one in the TM domain and three in sfGFP and the HA tags; Fig. S2A) and SPP contains five. Incubation of cells with BMH led to the appearance of CYP51A1TM-crosslinked products. These adducts were particularly prominent upon enrichment by immunoprecipitation (Fig. 2G). Interestingly, one of the crosslinked bands also reacted with an anti-SPP antibody, and considering the molecular mass of this band, it likely corresponded to an SPP dimer crosslinked to CYP51A1TM (Fig. 2G). The identity of the SPP–CYP51A1TM crosslink was confirmed by depletion of SPP (Fig. 3A) and mutation of the cysteine residues on CYP51A1TM (Fig. S2B,C). Moreover, SPP crosslinked specifically with CYP51A1TM but not with Erg11TM, a topologically similar model substrate of the MARCHF6 ERAD complex that has been shown to be degraded in an SPP-independent manner (Fig. S3A,B) (van de Weijer et al., 2020). Thus, CYP51A1TM and CYP51A1 bind directly to and are processed by SPP.

### SPP triggers ERAD of CYP51A1

Acute inhibition of ERAD with the p97 inhibitor CB-5083 led to an increase in the CYP51A1TM–SPP crosslink, suggesting that this interaction occurs early during CYP51A1TM degradation (Fig. 2G). A similar increase in CYP51A1TM–SPP crosslink was observed upon depletion of RNF185, suggesting that the binding occurred prior to CYP51A1TM ubiquitylation (Fig. 3A). Therefore, we asked whether SPP activity was required for CYP51A1TM ubiquitylation. Indeed, depletion of SPP resulted in a strong reduction of CYP51A1TM ubiquitylation (Fig. 3B). Altogether, these data indicate that SPP-catalysed N-terminal trimming triggers CYP51A1TM ubiquitylation by the RNF185–MBRL complex and subsequent degradation by the proteasome.

### SPP regulates CYP51A1 but no other cytochrome P450 enzymes

**Fig. 4.**
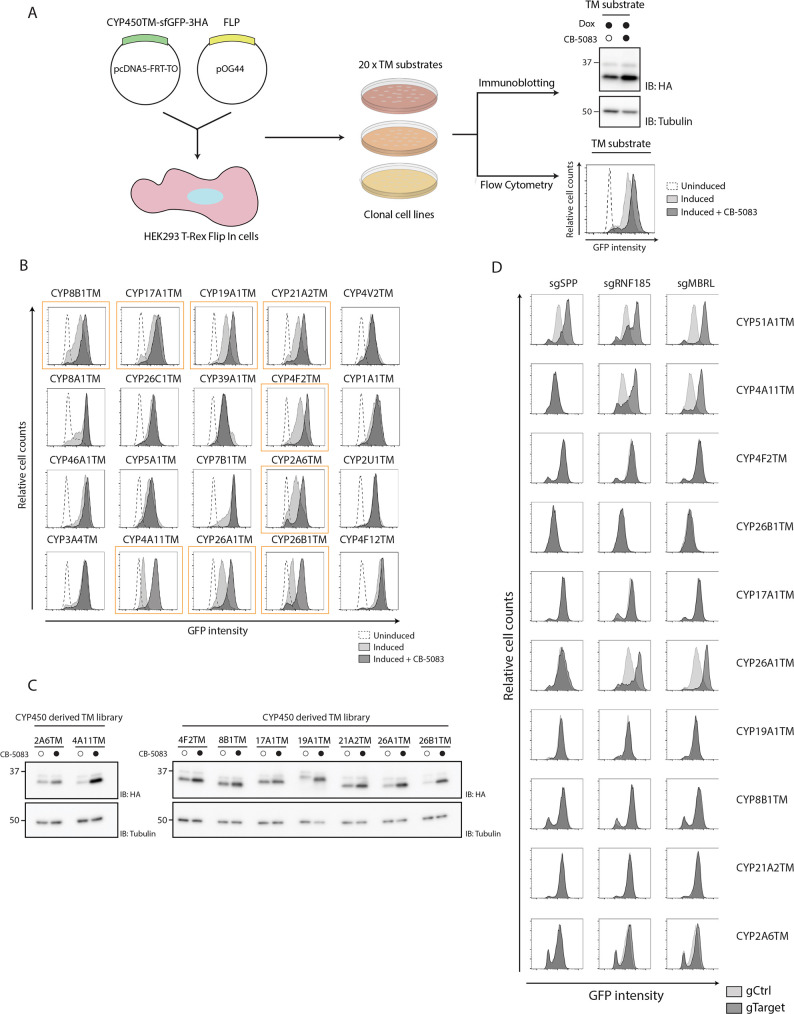
**SPP is required for degradation of CYP51A1TM but not of other CYP450 family members.** (A) Workflow to generate and validate HEK293 T-Rex Flip In cell lines stably expressing sfGFP–3×HA-tagged model substrates. Each cell line integrating a tagged model substrate was screened for expression of target constructs as well as stabilization upon treatment with the p97 inhibitor CB-5083 (4 h) by immunoblotting (IB) and flow cytometry. Dox, doxycycline induction. (B) Flow cytometry analysis of CYP450-derived model substrates in HEK293 T-Rex Flip In cells left uninduced (dotted line), induced with doxycycline (light grey), or induced and incubated for 4 h with p97 inhibitor (dark grey, CB-5083). Nine bona fide ERAD substrates are highlighted with an orange border. (C) Immunoblot analysis of HEK293 T-Rex Flip In cells expressing unstable CYP450-derived model substrates, as seen in B. Model substrate expression was induced with doxycycline and, where indicated, cells were incubated with the p97 inhibitor CB-5083 for 4 h. Molecular masses are indicated in kDa. (D) Flow cytometry analysis of unstable CYP450-derived model substrates in HEK293 T-Rex Flip In cells transfected with plasmids encoding gRNAs (sg) targeting the indicated genes (dark grey, gTarget) versus cells transfected with a control plasmid (light grey, gCtrl). Data in B–D are representative of three independent experiments.

CYP51A1 is a member of a large family of cytochrome P450 (CYP450) enzymes involved in a variety of functions such as lipid biosynthesis and drug metabolism (Nebert and Russell, 2002; Bishop-Bailey et al., 2014). Like CYP51A1, these enzymes are single-pass type III membrane proteins, with an N-terminal TM segment through which they associate with organellar membranes, mostly ER and mitochondria, followed by a conserved catalytic domain in the cytosol (Black, 1992; Monk et al., 2014). To test whether SPP has a general role in regulating the stability of CYP450 proteins localized to the ER, we developed a library of simplified model substrates consisting of the N-terminal membrane domains of 20 CYP450s (Table S1). Like CYP51A1TM, N-terminal regions of the various CYP450s were fused to sfGFP and HA tags and expressed under a doxycycline-inducible promoter in HEK293 T-Rex Flip In cells (Fig. 4A). Of the 20 CYP450s tested, nine were ERAD substrates, as their steady-state levels were increased upon acute inhibition of the general ERAD factor p97 (Fig. 4B,C). Turnover by ERAD of these nine CYP450 model substrates was further confirmed with gRNAs against SPP, RNF185 and MBRL (Fig. 4D). Remarkably, none of these nine ERAD substrates required SPP for their degradation. Thus, SPP has a highly selective role in triggering the ERAD of CYP51A1.

### SPP recognizes the CYP51A1 AH if aberrantly inserted in the membrane

**Fig. 5.**
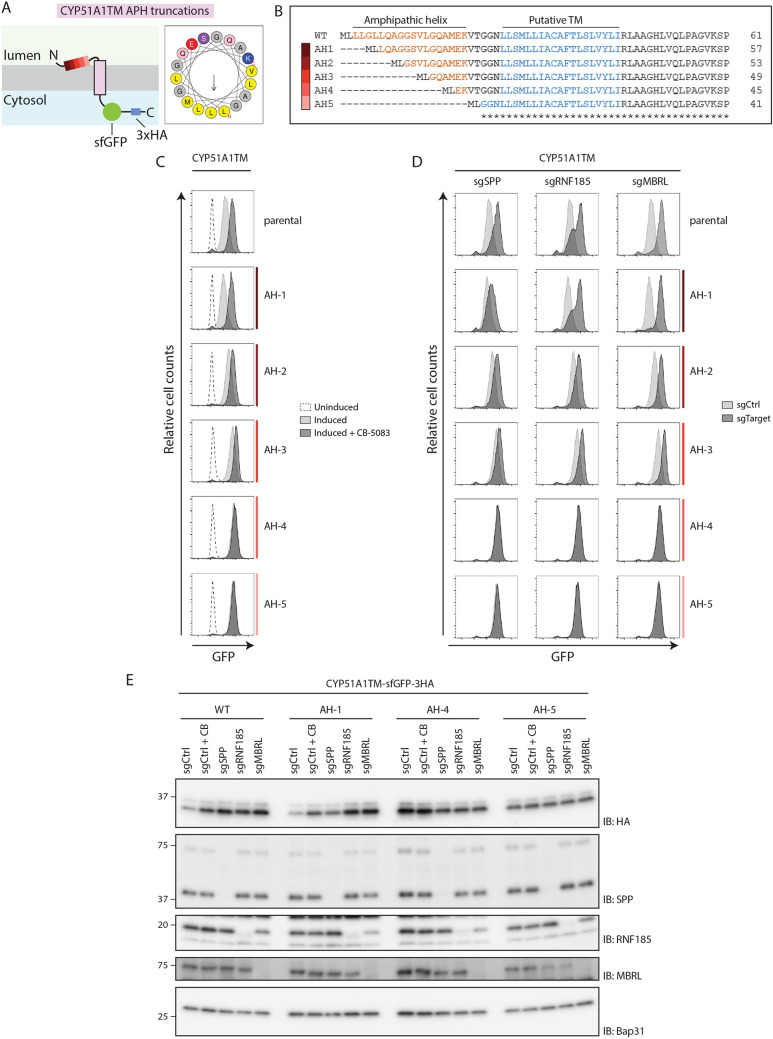
**SPP recognizes the AH of CYP51A1TM.** (A) Left: schematic depiction of CYP51A1TM, highlighting the region of the AH. The AH region is colour-coded to represent the truncations analysed in this study (see B). Right: helical wheel projection of the N-terminal AH generated using HeliQuest (Gautier et al., 2008). Arrow indicates direction and magnitude of the hydrophobic moment. Color coding was achieved by running the HeliQuest analysis tool using default parameters. Namely, hydrophobic residues are shown in yellow (valine, leucine and methionine), polar residues are shown in purple (serine), basic residues are shown in dark blue (lysine), acidic residues are shown in red (glutamic acid), glutamine is shown in pink, small residues (alanine and glycine) are shown in grey. APH, amphipathic helix. (B) Sequence alignment of AH truncation mutants of CYP51A1TM. AH and putative TM domain are coloured orange and blue, respectively. Colour coding represents the extent of the truncations, in line with A. Asterisks indicate sequence present in all truncations. (C) Analysis of the steady-state levels of AH truncation mutants of CYP51A1TM as shown in B. Flow cytometry analysis of doxycycline-induced expression of AH truncations in HEK293 T-Rex Flip In cells that were left uninduced (dotted line), induced (light grey), or induced and incubated for 4 h with p97 inhibitor (dark grey; CB-5083, 2.5 µM). (D) Same as C, but cells were transfected with plasmids encoding gRNAs (sg) targeting the genes indicated. Fluorescence intensity of construct expression upon gene KO (dark grey, sgTarget) was compared to cells transfected with a control plasmid (light grey, sgCtrl). (E) Same as C and D, cells expressing the truncated constructs were either treated with p97 inhibitor CB-5083 (4 h; CB) or transfected with plasmids encoding gRNAs targeting indicated genes and analysed by immunoblotting (IB). Steady-state levels of the model substrate were compared to cells transfected with a control sgRNA (sgCtrl). Molecular masses are indicated in kDa. Data in C–E are representative of three independent experiments.

Analysis of the various CYP450s revealed that CYP51A1 has an N-terminal AH that is not apparent in other family members (Fig. 5A). The N-terminal AH is conserved in the yeast CYP51A1 homologue Erg11, as revealed by a crystal structure (Monk et al., 2014). We tested whether this AH was a prerequisite for the SPP-dependent degradation of CYP51A1TM. To this end, we generated AH truncations, deleting four residues at a time corresponding to an α-helical turn (Pauling et al., 1951) (Fig. 5B). Remarkably, a gradual reduction in the AH length resulted in reduced SPP dependence and degradation by ERAD (Fig. 5C–E; Fig. S4). Complete loss of SPP dependence resulted in a stable CYP51A1TM truncation (Fig. 5C–E). This is in agreement with our previous observation that swapping the CYP51A1 AH with a distinct AH results in a stable protein (van de Weijer et al., 2020). Altogether these data indicate that SPP-catalysed cleavage of the AH initiates CYP51A1 ERAD by generating a degron that is recognized by the RNF185–MBRL complex.

**Fig. 6.**
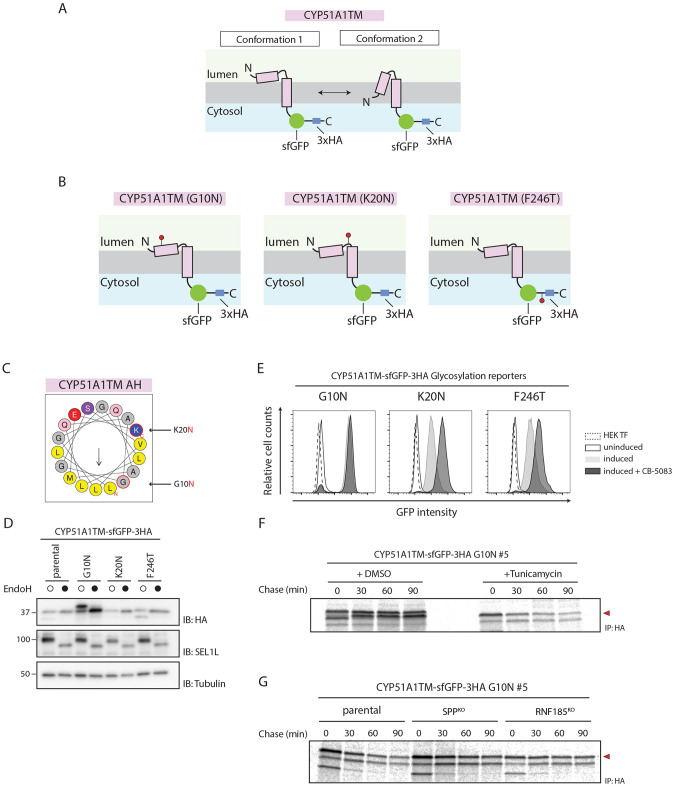
**The CYP51A1TM AH adopts two distinct topologies, with only one conformation being recognized by SPP for quality control.** (A) Schematic depiction of the two proposed topologies adopted by CYP51A1TM. Conformation 1 represents a pool of substrate with the AH in the ER lumen that is stable and independent of ERAD. Conformation 2 represents a substrate with a type II-oriented membrane span that would be unstable and susceptible for SPP-mediated quality control. (B) Cartoon representation of the three glycosylation reporters used in this study. The indicated amino acid substitutions were made to introduce glycosylation sites (shown in red) on the substrate at positions corresponding to either the AH (G10) region, the region juxtaposed to the TM segment (K20) or the cytosolic region (F246). (C) Helical wheel projection of the N-terminal AH of CYP51A1. The schematic was generated using HeliQuest (Gautier et al., 2008). Residues that were mutated to introduce artificial glycosylation sites at the N terminus (G10N and K20N) are highlighted. Central arrow indicates direction and magnitude of the hydrophobic moment. Color coding was achieved by running the HeliQuest analysis tool using default parameters. Namely, hydrophobic residues are shown in yellow (valine, leucine, methionine), polar residues are shown in purple (serine), basic residues are shown in dark blue (lysine), acidic residues are shown in red (glutamic acid), glutamine is shown in pink, small residues (alanine and glycine) are shown in grey. (D) HEK293 T-Rex Flip In cells expressing CYP51A1TM with glycosylation sites at the indicated positions were analysed by Endo H treatment and immunoblotting (IB). ER membrane-resident essential adaptor protein of E3 ubiquitin ligase HRD1, SEL1L, was used as a positive control for Endo H treatment. Molecular masses are indicated in kDa. (E) Assessment of the effect of glycosylation status on degradation of CYP51A1TM reporters. HEK293 T-Rex Flip In cells expressing the CYP51A1TM mutants shown in B were left uninduced (black line), induced (light grey), or induced and incubated for 4 h with p97 inhibitor CB-5083 (dark grey). Parental Flp-In HEK293 cells without the transgenic substrate were used as a control (dashed line, HEK TF). Steady-state levels of each reporter were analysed by flow cytometry. (F) Pulse–chase analysis of CYP51A1TM (G10N). The turnover of CYP51A1TM (G10N) glycosylation reporter was visualized by metabolic labelling and pulse–chase analysis using a ^35^S-labelled methionine/cysteine mix. HEK293 T-Rex Flip In cells expressing the reporter were labelled with radioactive isotopes for 15 min, and the target protein was recovered by HA immunoprecipitation (IP) in control cells treated with DMSO or in cells treated with the glycosylation inhibitor tunicamycin for 4 h at the chase times indicated and analysed by autoradiography. Red arrowhead marks the substrate specific band. (G) Same as F, but cells were transfected with plasmids encoding gRNAs targeting the genes indicated. Cells were treated with tunicamycin (4 h) to inhibit glycosylation and analysed by autoradiography. Red arrowhead represents the substrate specific band. Data in D–G are representative of three independent experiments.

SPP substrates identified so far display a type II membrane topology (N terminus in the cytosol). Therefore, our finding that SPP triggers CYP51A1 ERAD upon recognition of an AH predicted to localize to the ER lumen was surprising. Since an *in silico* prediction tool for ER-targeting signal sequences gave a low score to the CYP51A1 AH (Fig. S5A), we hypothesized that it could adopt distinct topologies: a luminal AH configuration and a type II-oriented TM span mimicking a signal sequence (Fig. 6A). This hypothesis was further supported by artificial intelligence (AI)-guided structural predictions (Jumper et al., 2021; Del Alamo et al., 2022; Abramson et al., 2024), which predicted that the AH does not interact with the rest of the CYP51A1 protein and adopts multiple positions and conformations (Fig. S5B–D). Interestingly, some of the top-ranked states predict the AH in a type II-like topology (Fig. S5B–D). To more directly test if CYP51A1 AH can adopt a type II orientation, we asked whether it could replace the signal sequence of bovine prolactin, a prototypical secreted protein (Fig. S5E). Indeed, we observed that CYP51A1 AH could efficiently promote prolactin secretion from cells (Fig. S5F).

According to our hypothesis and the preferred substrate topology reported for SPP (Weihofen and Martoglio, 2003), only the CYP51A1 molecules with the AH inserted into the membrane would be recognized and trimmed by SPP, and subsequently degraded by ERAD (Fig. 6A). To test this model, we investigated the topology of CYP51A1TM by introducing glycosylation reporters at three positions, corresponding to the AH (G10), juxtaposed to the TM segment (K20) and in the cytosolic region (F246) (Fig. 6B,C). Endoglycosidase H (Endo H) digestion indicated that the cytosolic reporter was not glycosylated, as expected (Fig. 6D). Similarly, we did not detect modification of the reporter adjacent to the TM segment, likely due to its proximity to the membrane (Nilsson and von Heijne, 2000). CYP51A1TM (G10N), which had the AH glycosylation reporter, was partially glycosylated, suggesting that only a fraction of CYP51A1TM molecules expose the AH to the ER lumen, where the glycosylation activity resides. Interestingly, glycosylation within the AH resulted in higher steady-state levels of CYP51A1TM (G10N), suggesting that the modification prevented its degradation (Fig. 6E). Moreover, and in contrast to CYP51A1TM and the two other glycosylation reporters, CYP51A1TM (G10N) levels did not increase upon p97 inhibition, suggesting that it was no longer an ERAD substrate (Fig. 6E). Therefore, modification of the AH with a bulky hydrophilic glycan appears to impede CYP51A1TM (G10N) recognition by SPP. Other modifications at the N terminus of CYP51A1TM, such as addition of epitope tags, also prevented CYP51A1TM recognition and degradation by ERAD (Fig. S6). These data are consistent with a model where the CYP51A1 AH adopts distinct topologies. Modifications favouring ER lumen localization of the AH prevent CYP51A1 ERAD.

This model predicts that conditions that prevent glycosylation of CYP51A1TM (G10N) should convert it into an ERAD substrate, similar to CYP51A1. To test this, we used pulse–chase experiments to study the turnover of CYP51A1TM (G10N) in cells treated with tunicamycin, a well-characterized inhibitor of N-linked glycosylation. Consistent with the model above, whereas glycosylated CYP51A1TM (G10N) was stable, blocking its glycosylation with tunicamycin rendered the protein unstable (Fig. 6F). Importantly, under tunicamycin treatment conditions, the degradation of CYP51A1TM (G10N) depended on SPP and RNF185 (Fig. 6G). These data indicate that the CYP51A1 AH can normally adopt two conformations, with only one being dependent on SPP for degradation.

SPP activity ensures that CYP51A1 is present in the ER membrane with one defined topology. We asked whether SPP had a general role in topology surveillance by analysing a subset of multi-pass membrane proteins. We focused on G-protein-coupled receptors (GPCRs), since members of this protein family have been shown to interact with SPP (Crawshaw et al., 2004; Schrul et al., 2010; Chitwood and Hegde, 2020). Moreover, factors controlling GPCR topogenesis, such as the ER membrane protein complex (EMC), and tools to interrogate this process have been established (Chitwood et al., 2018). To this end, we expressed the GPCRs β1 adrenergic receptor (β1AR, also known as ADRB1), rhodopsin (Rho) and type-2 angiotensin II receptor (AGTR2) appended to C-terminal GFP and RFP reporters separated by a viral P2A sequence (Fig. S7A,B). Depletion of the core EMC subunit EMC3 strongly impaired the biogenesis of all three GPCRs (Fig. S7C), as previously shown (Chitwood et al., 2018). The low levels of GPCRs in EMC3-deficient cells were restored to normal levels by acute p97 inhibition, suggesting that ERAD is responsible for GPCR degradation in cells lacking EMC. In contrast, depletion of SPP alone or together with EMC3 did not affect GPCR levels, suggesting that SPP is not essential for their quality control (Fig. S7C). Given that multiple degradation signals might be present in full-length GPCRs, we analysed the role of SPP in the quality control of a simplified model substrate encompassing two TM segments of rhodopsin, OP117 (Fig. S7D) (Crawshaw et al., 2004; Schrul et al., 2010). This construct was fused to moxVenus and HA tags and contained a glycosylation site proximal to the N terminus that informed us of its topology. As in the case of the full-length GPCRs, EMC3 depletion had a strong impact on the biogenesis of OP117. In contrast, depletion of the dislocase ATP13A1, which is involved in membrane extraction of proteins with reversed topology (McKenna et al., 2022), had no effect. As with the full-length GPCRs, depletion of SPP, either alone or in various combinations, did not appear to interfere with the biogenesis and levels of the OP117 model substrate (Fig. S7E). Thus, SPP has a specific set of substrates, and the topology surveillance of GPCRs is independent of SPP.

### The AH of CYP51A1 is sufficient to trigger SPP-dependent ERAD

**Fig. 7.**
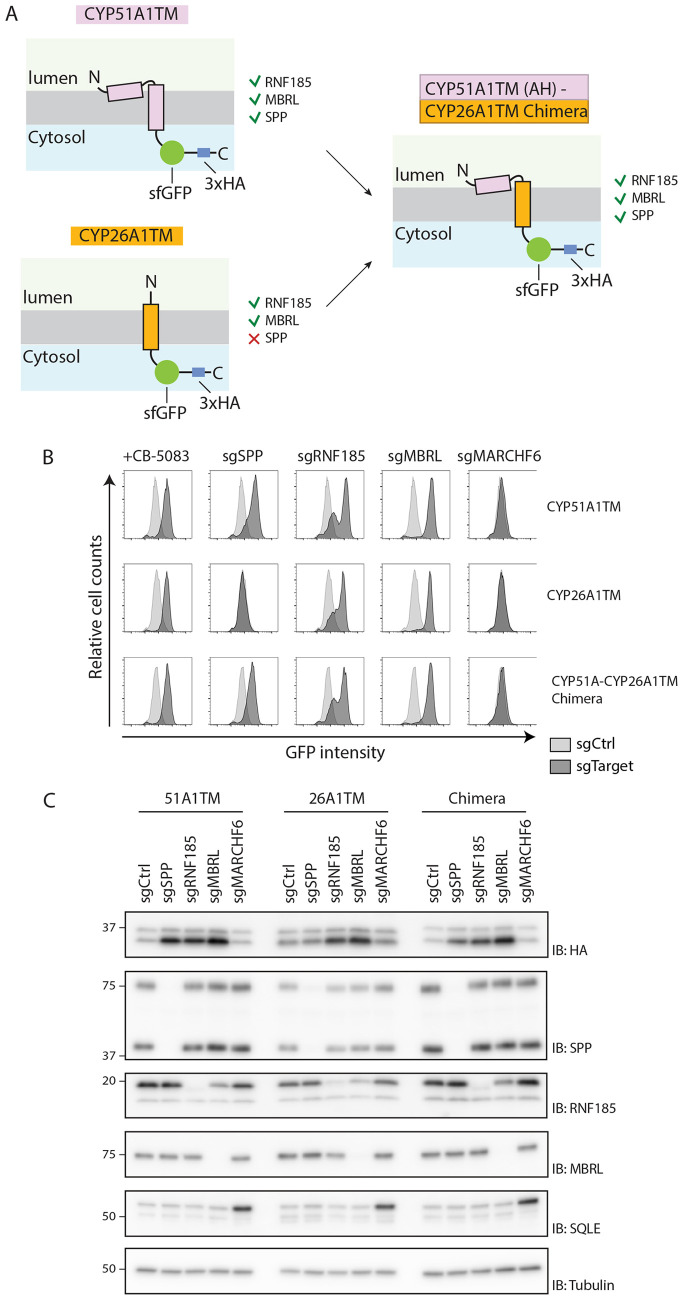
**The metastable CYP51A1TM AH is sufficient to trigger SPP-dependent quality control.** (A) Schematic depiction of two model substrates used in this experiment. CYP51A1TM is dependent on SPP, RNF185 and MBRL for quality control (top panel, in pink), whereas CYP26A1TM is SPP-independent (bottom panel, in orange). A chimeric construct generated by appending the N-terminal AH of CYP51A1TM on CYP26A1TM is shown on the right (pink–orange construct fusion). (B,C) Comparison of substrate dependencies on various ERAD factors. Flow cytometry (B) and SDS-PAGE (C) analysis of HEK293 T-Rex Flip In cells expressing the three constructs shown in A. Construct expression was analysed upon induction with doxycycline either in the presence of the p97 inhibitor CB-5083 (4 h) or following transfection with plasmids encoding gRNAs (sg) targeting the genes indicated. In B, fluorescence intensity was compared between cells transfected with a control gRNA plasmid (light grey, sgCtrl) versus a plasmid encoding the gRNA of interest (dark grey, sgTarget). In C, molecular masses are indicated in kDa. IB, immunoblot. Data in B and C are representative of three independent experiments.

Having shown that SPP-mediated quality control of CYP51A1 requires the AH, we then asked whether the presence of this AH was sufficient to confer SPP dependence to an ERAD substrate. To address this issue, we took advantage of CYP26A1TM, a substrate of the RNF185–MBRL complex that is degraded independently of SPP (Fig. 7A,B). CYP26A1TM was used to generate a chimera with the AH of CYP51A1 (Fig. 7A). The degradation of this chimera was still dependent on the RNF185–MBRL ERAD complex. However, in contrast to CYP26A1TM, the degradation of the chimeric protein required SPP (Fig. 7B,C). Thus, the CYP51A1 AH is necessary and sufficient to trigger SPP-dependent degradation of an ERAD substrate.

## DISCUSSION

**Fig. 8.**
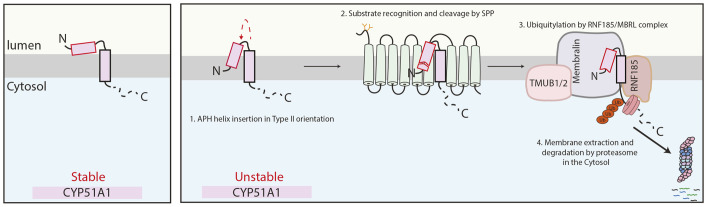
**Proposed model of CYP51A1 recognition by SPP.** In cells, the lanosterol demethylase CYP51A1 can exist in two distinct conformations, with only one conformation being recognized for membrane protein quality control. The early recognition event by SPP consists of an N-terminal cleavage within the AH of the substrate. This generates a truncated version of CYP51A1 that is subsequently recognized by the RNF185–MBRL E3 ubiquitin ligase complex, extracted from the ER membrane and degraded by the cytosolic proteasomes. APH, amphipathic helix.

In this study, we characterized how SPP promotes quality control of the lanosterol demethylase CYP51A1 and triggers a population of this enzyme to undergo ERAD-mediated degradation. We show that the CYP51A1 AH displays a metastable behaviour, being either localized to the ER lumen or inserting into the ER membrane with a signal sequence-like type II orientation. In the latter topology, the AH is recognized and cleaved by SPP, which triggers degradation by the RNF185–MBRL ubiquitin ligase ERAD complex (Fig. 8). Under our experimental conditions, a small fraction of CYP51A1 undergoes SPP-dependent degradation. In contrast, most of the CYP51A1TM, a truncated version lacking the cytosolic catalytic domain, is degraded by this quality control process, suggesting that presence of the cytosolic region intereferes with the localization of the AH to the ER lumen.

Interestingly, the yeast CYP51A1 homologue displays strong constraints on the relative orientation of cytosolic and membrane domains, as revealed by structural studies (Monk et al., 2014). These constrains are important for the cytosolic catalytic region to access its hydrophobic substrate (lanosterol) in the ER membrane (Monk et al., 2014). Although structural information for human CYP51A1 is not available, it is plausible that the insertion of the AH in a type II orientation interferes with CYP51A1 enzymatic activity. Future studies should test this possibility and evaluate the importance of SPP-mediated quality control in regulating the pool of active CYP51A1.

The behaviour of the AH and CYP51A1 turnover can also be controlled by other (extrinsic) factors, such as the lipid composition of the ER membrane or the presence of CYP51A1 partners (Park et al., 2017; Scott et al., 2020). The ER integral proteins PGRMC1 and PGRMC2 bind to CYP51A1 and other CYP450 enzymes and have been shown to affect their turnover (Hughes et al., 2007; McGuire et al., 2021). Thus, PGRMC1 and PGRMC2 would be good candidates to interfere with SPP-dependent turnover of CYP51A1; however, this does not appear to be the case (Fig. S8).

SPP has been previously implicated in the ERAD-mediated clearance of some TA proteins and the type II membrane protein XBP1u (Boname et al., 2014; Chen et al., 2014; Avci et al., 2019; Hsu et al., 2019; Yücel et al., 2019; McKenna et al., 2022). Like CYP51A1, these substrates contain type II-oriented membrane spans. Upon recognition, SPP promotes their cleavage within the plane of the membrane, which triggers their ubiquitylation by ERAD ubiquitin ligases. These events appear to occur in large biochemical assemblies with SPP interacting with the ubiquitin ligases, either TRC8 or MARCHF6, in the case of TA proteins or with the ERAD component DERLIN-1, in the case of XBP1u (Boname et al., 2014; Chen et al., 2014; Stefanovic-Barrett et al., 2018; Avci et al., 2024). Although a similar order of events occurs in the case of CYP51A1 degradation, we have no evidence that SPP interacts with the RNF185–MBRL complex. Thus, how CYP51A1 is handed from SPP to the ubiquitin ligase complex and whether this requires additional factors remains unclear.

SPP and its yeast orthologue Ypf1 have also been linked to quality control of multi-spanning membrane proteins. For SPP, this was mainly based on crosslinking experiments that identified direct SPP crosslinks to truncated GPCRs such as rhodopsin (Crawshaw et al., 2004; Schrul et al., 2010). However, in this study, we failed to observe an obvious role for SPP in the quality control of GPCRs, suggesting that SPP does not have a general role in the clearance of proteins with incorrect topology. Considering that SPP performs essential functions and its ablation in mice results in embryonic lethality (Aizawa et al., 2016), the critical SPP substrates remain elusive and should be investigated in the future.

## MATERIALS AND METHODS

### Cell culture

HEK293T-Rex Flip In cells (Flp-In T-Rex-293; Invitrogen) and HeLa cells (ATCC) were cultured at 37°C with 5% CO_2_ in Dulbecco's modified Eagle's medium (DMEM; Sigma-Aldrich), supplemented with penicillin-streptomycin (10 units/ml; Gibco), 10% fetal bovine serum (FBS; Gibco) and L-glutamine (2 mM; Gibco). Cells were routinely checked for mycoplasma contamination and confirmed to be negative. CB-5083 (Selleckchem , cat. no. S8101), is a potent and highly selective inhibitor of p97 ATPase. CB-5083 was dissolved in DMSO and added directly to cell culture medium to a final concentration of 2.5 μM and incubated for 4 h. Substrate expression was achieved by addition of doxycycline (Merck, cat. no. D9891) to a final concentration of 100 ng/ml in complete DMEM followed by overnight incubation.

### Transient transfection

Transient transfections were performed using 40 kDa linear polyethyleneimine (PEI MAX; Polysciences) (Durocher et al., 2002). For 12-well plates, 1 µg plasmid DNA and 2.5 µl of PEI per well were used. If not stated otherwise, cells were harvested 24 h after transfection. For inhibition of SPP, 50 µM (Z-LL)_2_-ketone (Sigma-Aldrich, cat. no. SML1442) was added from stock solutions in dimethyl sulfoxide (DMSO).

### Generation of stable HEK293 T-Rex Flip In cell lines

Stable cell lines, integrating target genes under a doxycycline inducible promoter were generated using TransIT-LT1^®^ reagent (Mirus Bio, cat. no. MIR 2305), according to the manufacturer's protocol. In brief, the day before the experiment, ∼10^6^ HEK293 TRex Flip In cells were seeded in a 6-well plate format in 2 ml complete DMEM. The following day, cells were co-transfected with pcDNA™5/FRT/TO (V652020; Thermo Fisher Scientific) plasmid carrying the gene of interest, together with the Flp recombinase plasmid (pOG44; V600520; Thermo Fisher Scientific) at a ratio of 1:9 with a total of 2.5 μg. At 24 h after transfection, the medium was replaced with fresh DMEM containing 150 μg/ml hygromycin B (Millipore) and 4 μg/ml blasticidin S (Millipore), and cells were selected for 2–3 weeks. Single colonies were then picked and counterselected with 100 μg/ml Zeocin (Invitrogen, cat. no. R25001) to validate successful integration of the target gene prior to analysis by flow cytometry and immunoblotting.

### Plasmids and molecular cloning

The original pcDNA5-FRT-TO plasmid was obtained from Invitrogen. Sequences encoding human ER-resident CYP450 enzymes were ordered as gene blocks (IDT), and KpnI-Bsp119I fragments were inserted in a pcDNA5-FRT-TO-based parental vector containing superfolder GFP (sfGFP) and haemagglutinin (3×HA tag) (van de Weijer et al., 2020) using standard restriction cloning techniques. Amino acid sequences of CYP450-derived model substrates used in this study are outlined in Table S1. The plasmid encoding human SPP with a C-terminal triple FLAG tag was cloned in a dual promoter lentiviral vector, as described previously (van de Weijer et al., 2014). The active site mutant of SPP (SPP D265A) was introduced by site-directed mutagenesis. Truncated AH constructs, cysteine mutants and glycosylation reporter mutants were generated using standard restriction cloning techniques. The fusion construct encoding the N-terminal AH of CYP51A1 (MAAAAGMLLLGLLQAGGSVLG) fused to prolactin with a single HA tag at the C terminus was ordered as a gene block cloned in pcDNA3.1+ (BioCat). Construct encoding the first 117 residues of bovine rhodopsin (OP117, Gene ID 509933) with C-terminal moxVenus and triple HA tags was ordered as gene block (IDT) and cloned into pcDNA5-FRT-TO. Stable cell lines expressing GPCR reporters with C-terminal GFP–P2A–RFP tags were a gift from Ramanujan Hegde (MRC Laboratory of Molecular Biology, Cambridge, UK) and have been described previously (Chitwood and Hegde, 2020). gRNAs for gene deletions were cloned in a dual promoter lentiviral vector, as described previously (van de Weijer et al., 2014). Cysteine-less versions of CYP51A1TM were generated by either replacing native cysteine residues of the sfGFP-3xHA portion of the construct at position 113, 135 and 343 with serine (for the GFP C-less CYP51A1TM construct) or by replacing cysteine residues in both the TM domain and the sfGFP-3xHA portion of the substrate at positions 34, 113, 135 and 343 with serine, respectively (for the full C-less CYP51A1TM construct). The N-terminal tags (3xFLAG, Myc or V5) were appended upstream of the CYP51A1TM coding sequence, separated by a 2× GGGGS linker. The chimeric CYP51ATM(AH)-CYP26A1TM construct was generated by appending the N-terminal amphipathic helix (AH) of CYP51A1TM (MLLLGLLQAGGSVLGQAMEK) to the CYP26A1TM coding sequence shown in Table S1. The Erg11TM construct was previously described (van de Weijer et al., 2020). Cysteine mutagenesis was conducted by replacing native cysteine residues with serine in the putative amphipathic helix at position 11 (alanine) or putative TM domain residues 34, 35, 36 and 37 (all isoleucine).

### Generation of CRISPR/Cas9-mediated KO cell lines

To generate CRISPR/Cas9-mediated KO cell lines, HEK293 T-Rex Flip In cells were transfected with target plasmid DNA using TransIT-LT1 reagent, according to manufacturer's protocol. The following day, complete medium was replaced with medium containing puromycin at a concentration of 2 μg/ml. 72 h after antibiotic selection, the medium was changed back to complete DMEM, and cells were seeded for analysis by flow cytometry and immunoblotting. The following gRNAs targeting specified genes were used: gSPP, 5′-GCCGTTATGCGGATCGCTGA-3′; gRNF185, 5′-GAATGGCGCTGGCGAGAGCGG-3′; gMBRL, 5′-CGAAGAACTCGAAGAGACGG-3′; gTRC8, 5′-GGCGCTCGAAGTGGCGCTC-3′; gMARCHF6, 5′-GACAAGATGGACACCGCGG-3′; gEMC3, 5′-GAAGTGATGATAACGATGGGT-3′; gATP13A1, 5′-GAACAGCGCCAACCGCCGGTA-3′; gPGRMC1, 5′-GCTCTACAAGATCGTGCGCG-3′; gPGRMC2, 5′-GGCGTTGGCGCTTCTGACGG-3′.

Non-specific gRNA was used as a negative control throughout the study. For generation of clonal KO cells, polyclonal populations carrying CRISPR/Cas9-mediated KOs were single-cell sorted using a BD FACS Aria III.

### Lentivirus production and transduction in adherent mammalian cell cultures

Lentivirus was produced in a 24-well plate format using commercially available TransIT-LT1 (Mirus, cat. no. MIR 2305 ) transfection reagents and second-generation packaging vectors following standard lentivirus production protocols (Krshnan et al., 2022b). In brief, 293T Lenti-X cells (Takara Bio, cat. no. 632180) were co-transfected with lentiviral and packaging plasmids (psPAX2, Addgene plasmid #12260 and pMD2.G, Addgene plasmid #12259) using the Mirus LT1 transfection reagent (Mirus, cat. no. MIR 2305) in 24-well tissue culture plate format. After 72 h, the medium was harvested. For transduction, cells were seeded in a 24-well tissue culture plate at 1.5×10^5^ cells for HEK cells. The next day, 200 μl of lentivirus was added onto the cells. After 24 h, the medium was replaced and cells were grown for further 24 h. Cells were expanded on a 10 cm tissue culture-treated Petri dish in the presence of antibiotic selection.

### Immunoblotting

If not stated otherwise, cells were lysed in 1× Laemmli sample buffer (0.2 M Tris-HCl pH 6.8, 6 % SDS, 30 % glycerol and 0.2 % Bromophenol Blue) and incubated on a ThermoMixer at 37°C for 15 min. Whole-cell lysates were then subjected to SDS-PAGE using Precast 26-well 4–20% Criterion TGX gels (Bio-Rad). Next, proteins were transferred to PVDF membranes (Bio-Rad) and blocked with 5% bovine serum albumin (BSA) or 5% milk for 1 h. Following this, membranes were incubated with antibodies of interest overnight in a cold room on a rocking platform. Subsequently, blots were washed three times with PBS containing 0.1% Tween 20 (PBS-T; Sigma-Aldrich, cat. no. P1739) and probed with secondary antibodies for 1 h. Reactive protein-specific bands were identified by ECL solution (Perkin Elmer) and visualized using an AI 600 (GE Healthcare).

Full details of primary and secondary antibodies used in this study are provided in Table S2. Primary antibodies were used following the manufacturers’ recommended protocols. All HRP-conjugated secondary antibodies for western blotting were used at 1:10,000. Western blot quantifications were performed in Image Studio software LiCor (https://www.licor.com/bio/image-studio/) using the measure tool to obtain band intensities. To account for loading differences, the intensity of the loading control in each lane was taken and normalized to an average of 1. The band intensity was then divided by the normalized loading control amount to obtain the intensity value. Original, uncropped western blotting data used in the figures are displayed in Figs S9–S22.

### Cysteine-based crosslinking

Crosslinking was carried using a non-cleavable, membrane-permeable crosslinker – bismaleimidohexane (BMH). The day before the experiment, 1.2 million cells were seeded in a 6-well plate format in complete DMEM, and target protein expression was induced with 100 ng/ml doxycycline for 16 h. Next day, cells were trypsinized, resuspended in complete DMEM, centrifuged at 500 ***g*** for 5 min and washed three times with ice-cold PBS (Sigma). Cell pellets were then resuspended in 2 ml PBS and split into two pre-chilled 2 ml Eppendorf tubes (for use with or without BMH). 50 mM BMH crosslinker was dissolved in DMSO and added to a final concentration of 1 mM directly into the tube, and crosslinking was performed in a cold room in the dark on ice for 45 min. Quenching was performed with 25 mM dithiothreitol (DTT) for 15 min on ice. Following quenching, the cell suspension was centrifuged at 500 ***g*** for 5 min. Supernatant containing quenched BMH was removed by aspiration, and the cell pellet was lysed in 1% SDS/TBS buffer (1% SDS, 1 M TBS pH 7.5) supplemented with cOmplete protease inhibitor cocktail (Sigma) and Benzonase (Sigma) on a ThermoMixer (1000 rpm) for 20 min, at 37°C. Following lysis, SDS concentration was diluted to 0.2% with RIPA buffer (50 mM Tris-HCl pH 7.5, 150mM NaCl, 1% Triton X-100, 0.5% sodium deoxycholate and 0.1% SDS), and the lysate was centrifuged at 14,000 ***g*** for 15 min. Post-nuclear supernatants were collected into fresh 1.5 ml Eppendorf tubes and incubated with anti-HA magnetic beads (Pierce) for 2 h at 4°C rotating head-over-head. Beads were washed three times with RIPA buffer, and proteins were eluted in 1× Laemmli sample buffer (without DTT). DTT was then added to a final concentration of 100 mM, and immunoblotting was conducted as described above.

### Substrate ubiquitylation assay

The day before the experiment, ∼2 million cells were seeded in a 10 cm dish format, and protein expression was induced with 100 ng/ml doxycycline for 24 h. The next day, cells were lysed in RIPA buffer (50 mM Tris-HCl pH 7.5, 150 mM NaCl, 1% Triton-X100, 0.5% sodium deoxycholate, 0.1% SDS) supplemented with cOmplete protease inhibitor cocktail (Roche) and 20 mM *N*-ethylmaleimide (Sigma). Cell lysis was performed in a cold room, rotating head-over-head for 1 h. Cell nuclei and debris were pelleted by centrifugation in a pre-cooled centrifuge at 20,000 ***g*** for 20 min. Post-nuclear supernatants were isolated and incubated with anti-HA magnetic beads (Pierce, Thermo Fisher Scientific) in the cold room with head-over-head rotation for 2 h. Beads were washed three times with RIPA buffer, and samples were eluted in 30 μl of 1× Laemmli sample buffer without DTT at 65°C for 10 min. Following this, DTT was added to a final concentration of 100 mM, and immunoblotting was performed as described above.

### Co-immunoprecipitation

The day before the experiment, ∼5×10^6^ cells were seeded in a 10 cm dish format, and protein expression was induced with doxycycline for 24 h. The next day, cells in 10 cm dishes were washed with cold PBS on ice and lysed by scraping in 1% DMNG (Anatrace) lysis buffer (50 mM Tris-HCl pH 7.5, 150 mM NaCl) containing cOmplete protease inhibitor cocktail (Roche). The lysates were transferred into ice cold 2 ml Eppendorf tubes and rotated head-over-head for 1 h at 4°C. Cell nuclei and debris were pelleted by centrifugation in a pre-cooled centrifuge at 20,000 ***g*** for 20 min. Post-nuclear supernatants were isolated and incubated with anti-HA, anti-FLAG or Protein-G magnetic beads (Pierce, Thermo Fisher Scientific) in a cold room with head-over-head rotation for 2 h. When Protein-G magnetic beads were used for native immunoprecipitation, the lysates were pre-incubated with the target antibody (anti-SPP, 1 μg; cat. no. ab190253, Abcam) or IgG (rabbit, 1 μg; GeneTex, cat. no. GTX35035) before Protein-G beads were added and lysates were incubated for another 1 h in the cold room (4°C) with head-over-head rotation. After three washes in 0.1% DMNG washing buffer (50 mM Tris-HCl pH 7.5, 150 mM NaCl), proteins were eluted in 1× sample buffer for 20 min at 37°C or using 3×FLAG peptide (500 ng/μl; Sigma-Aldrich) for 30 min on ice. In the case of elution using FLAG-peptide, the eluate was transferred to a new 1.5 ml Eppendorf tube and denatured by adding Laemmli sample buffer containing DTT. Denatured material was either stored at −20°C or used directly for immunoblotting as described.

### Flow cytometry

Cells were trypsinized, washed once with ice-cold PBS and re-suspended in FACS buffer (1 mM EDTA, 2% FBS in PBS). Cells were then assessed for expression of target constructs by fluorescence using a BD LSRFortessa X-20, and data were processed using FlowJo 10.4 (https://www.flowjo.com/). At least 10,000 cells per sample were analysed, gating on the main population in the forward scatter/side scatter (FSC/SSC) plot.

### Pulse–chase labelling

Cells from confluent T175 flasks (∼10–15 million cells) were recovered by trypsinization, washed with PBS and resuspended in 100 μl of starvation medium (Met- and Cys-free medium; cat. no. 21013024, Sigma) and labelled with 2 mCi [^35^S] methionine/cysteine labelling mix (PerkinElmer) for 15 min at 37°C. Labelled cells were chased with complete medium for the time points indicated. Treatment with 2.5 μg/ml tunicamycin (Cell Signaling Technology, cat. no. 12819S) was undertaken in DMEM for 4 h. Cells were washed once with ice-cold PBS and either stored at −20°C or directly lysed in RIPA buffer (50 mM Tris-HCl pH 7.5, 150 mM NaCl, 1% Triton-X100, 0.5% sodium deoxycholate, 0.1% SDS) supplemented with cOmplete protease inhibitor cocktail (Roche) for 1 h with head-over-head rotation in a cold room. Post-nuclear supernatants were isolated, transferred to fresh, pre-chilled 1.5 ml Eppendorf tubes and incubated with anti-HA magnetic beads (Pierce, Thermo Fisher Scientific) in a cold room with head-over-head rotation for 2 h. Beads were washed three times with RIPA buffer, and samples were eluted in 30 µl of 1× sample buffer without DTT at 65°C for 10 min. Following this, DTT was added to a final concentration of 100 mM and immunoblotting was performed as described above. Radiolabelled proteins were visualized on Typhoon FLA 9500 scanner and analysed using Fiji software (https://imagej.net/software/fiji/).

### Cycloheximide chase

The day before the experiment, 1.2×10^5^ cells were seeded in a 24-well plate, pre-coated with poly-L-lysine, and protein expression was induced with 100 ng/ml doxycycline for 24 h. The next day, cells were incubated with cycloheximide (50 μg/ml) for the indicated time, after which the medium was aspirated and the cells were directly lysed in 1× Laemmli sample buffer containing benzonase (Sigma), cOmplete protease inhibitor cocktail (Roche) and 100 mM DTT. Lysates were incubated on a ThermoMixer for 20 min after which the denatured lysates were either frozen or used directly for immunoblotting.

### Glycosylation assay

The glycosylation assay was performed according to standard manufacturer's protocol (NEB, cat. no. P0702S). In brief, cells in 24-well plates were lysed in 200 μl of RIPA buffer for 1 h in a cold room with head-over-head rotation. Following this, lysates were centrifuged at 20,000 ***g*** for 20 min at 4°C. Post-nuclear supernatant was collected, split into two parts in 1.5 ml Eppendorf tubes (for use with or without Endo H) and supplemented with 10× Denaturing buffer (NEB, cat. no. P0702S). Lysates were incubated at 95°C for 10 min after which denatured samples were supplemented with Buffer 3 (NEB, cat. no. P0702S) and 1000 units (2 μl of 500,000 units/ml) of Endo H (NEB, cat. no. P0702S) and incubated for a further 2 h at 37°C with mild shaking (400 rpm). Following this, samples were supplemented with 3× Laemmli sample buffer and loaded for SDS-PAGE.

### Secretion assay

For the analysis of secreted proteins, culture medium was collected from the wells before harvesting the cells and centrifuged to remove cell debris, followed by precipitation using 10% trichloroacetic acid. After centrifugation at 4°C, 15,000 ***g*** for 5 min, supernatant was removed and the pellet was washed twice with 100% acetone. Dried protein pellet was then resuspended in equal volume of SDS-sample buffer as the corresponding lysate samples for equivalent loading. Samples were incubated at 65°C for 15 min before being loaded for SDS-PAGE. When indicated, cells were pre-treated with 2 µM cavinafungin (gift from Martin Spiess, University of Basel, Switzerland) for 16 h before harvesting.

### Fluorescence microscopy

One day prior, HEK293 T-Rex Flip In cells were seeded onto round coverslips in a 12-well plate pre-coated with poly-L-lysine. 24 h later, the medium was replaced with complete DMEM supplemented with 100 ng/ml doxycycline for 16 h to induce construct expression. Cell culture medium was aspirated, and the cells were washed with PBS once. Following this, cells were fixed by adding 1 ml of 4% methanol-free paraformaldehyde (PFA; 28908, Thermo Fisher Scientific) in PBS for 15 min at 37°C. PFA was removed, and fixed cells were washed three times with PBS and incubated in 1 ml blocking buffer (0.1% saponin, 1% BSA in PBS) for 15 min at room temperature. Following two washes with PBS, permeabilized cells were incubated with primary antibody in blocking buffer for 1 h in the dark, by placing the coverslips onto pre-spotted antibody aliquots on parafilm underneath a custom aluminium foil-covered moisturising chamber. Cover slips were then transferred to a fresh 12-well plate and washed with blocking buffer three times. Fluorophore-conjugated secondary antibody incubation was done in a similar way to primary antibody incubation. Coverslips were washed with PBS and incubated in 1 ml of DAPI-containing PBS for 5 min at room temperature, followed by three 5 min washes with PBS. Coverslips were then mounted on microscopy glass slides in non-hardening mounting medium (H-1900; Vector Laboratories) and sealed with nail polish. Samples were imaged immediately after full solidification of mounting medium (∼2 h) or kept at 4°C until further processing. Primary antibodies for immunofluorescence microscopy were against: GFP (mouse; Sigma 11814460001; RRID:AB_390913; 1:1000); Bap31 (mouse; Enzo ALX-804-601-C100; RRID:AB_205079; 1:1000). Fluorescently labelled secondary antibodies for immunofluorescence were used at 1:400. See Table S2 for details. Cell nuclei were stained with DAPI (Thermo Fisher Scientific , cat. no. 00-4959-52).

Fixed cells on slides were imaged at room temperature using a Zeiss Axio Observer Z1 equipped with a complementary metal-oxide semiconductor (CMOS) camera (Hamatsu ORCA-Flash4.0), controlled by 3i Slidebook 6.0 software (https://www.intelligent-imaging.com/Slidebook). The system was equipped with a Plan-APOCHROMAT ×63 1.4 objective, used with an immersion oil (Immersol W 2010, Carl Zeiss; refractive index of 1.518). The resulting images were exported as TIFF files.

### AI-guided structural modelling

The default AlphaFold2 CYP51A1 model was downloaded from the EBI database (https://alphafold.ebi.ac.uk/entry/Q16850) (Varadi et al., 2022). AlphaFold2 with MSA subsampling was run using ColabFold (Mirdita et al., 2022). The max_msa setting was set to 16:32, num_recycles was set to 1, and num_seeds was set to 8, to generate 40 models. The top 10 were ranked according to predicted local-distance difference test (pLDDT) values, with pLDDT plots for the top five shown in Fig. S5C. AlphaFold3 models were run on the webserver (https://golgi.sandbox.google.com/) using default settings.

### Quantification and statistical analysis

Western blot data were quantified using Image Studio software (Li-Cor, https://www.licor.com/bio/image-studio/) or ImageJ (https://imagej.net/ij/), and graphs were plotted using GraphPad Prism (https://www.graphpad.com/). Representative images of at least three independent experiments are shown.

## Supplementary Material



10.1242/joces.262333_sup1 Supplementary information
