# Supplementary material for: Topology surveillance of the lanosterol demethylase CYP51A1 by signal peptide peptidase
Source: J Cell Sci. 2024 Dec 12;137(23):jcs262333. doi: 10.1242/jcs.262333 (PMC11827857; doi:10.1242/jcs.262333)
Supplement: Supplementary information [file joces-137-262333-s1.pdf]

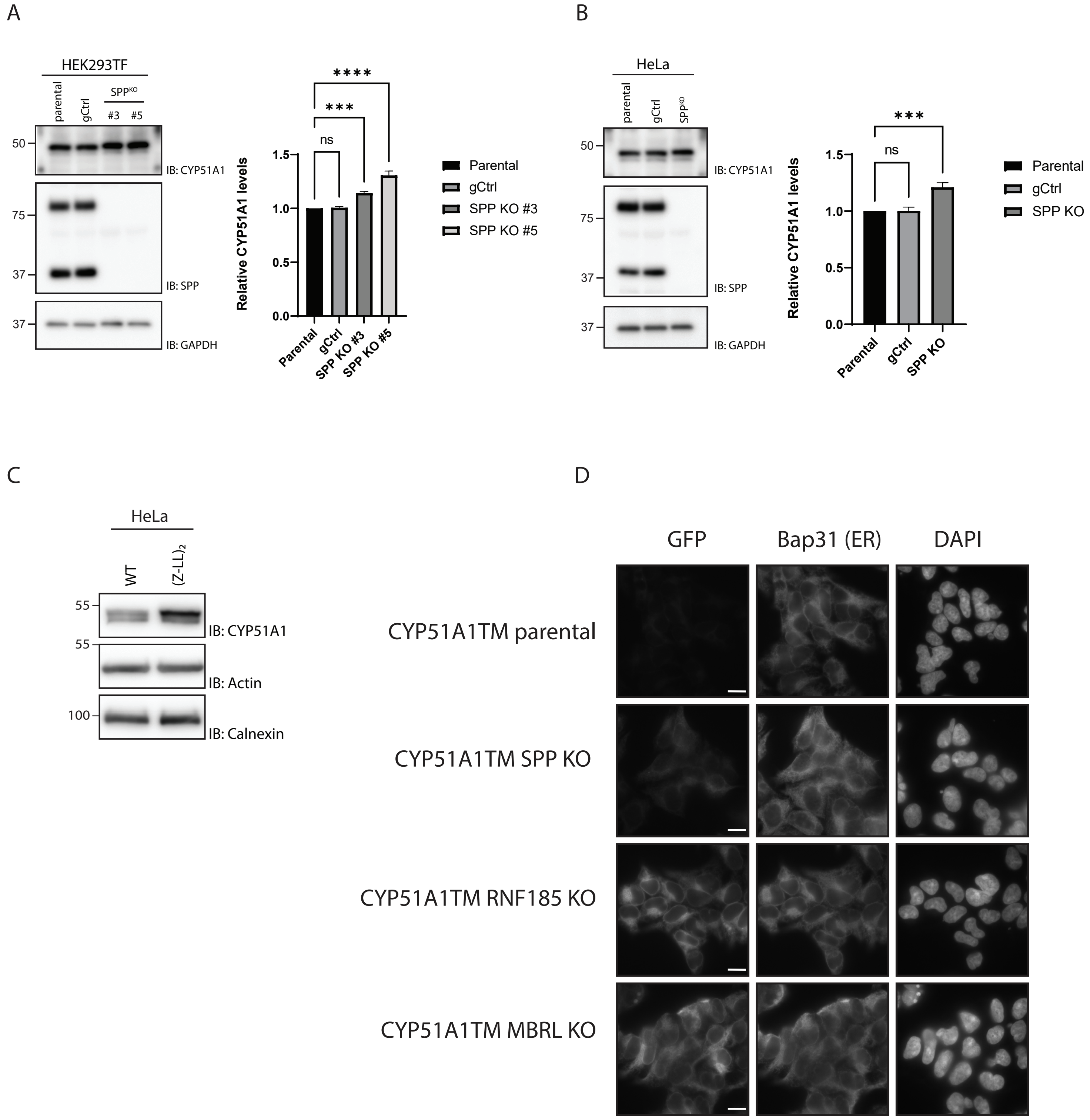

Fig. S1. Endogenous CYP51A1 is a substrate of SPP

A. Western blot analysis showing steady state levels of endogenous CYP51A1 in HEK-293TRex Flip in cells. GAPDH was used as a loading control. Quantification shows mean ± SD, n=3. Three biological replicates were analysed. Statistical analysis was performed using ordinary one-way ANOVA, p value for *** = 0.0002 and **** = <0.0001
B. Western blot analysis showing steady state levels of endogenous CYP51A1 in HeLa cells. GAPDH was used as a loading control. Quantification shows mean ± SD, n=3. Three biological replicates were analysed. Statistical analysis was performed using ordinary one-way ANOVA, p value for *** = 0.0003.
C. Immunoblot analysis showing steady state levels of endogenous CYP51A1 in HeLa cells upon 16 h treatment with $(Z\text{-}LL)_2$-ketone or DMSO. Actin and calnexin was used as loading control.
D. CYP51A1TM localizes to the ER. HEK293TRex Flip In cells expressing CYP51A1TM were depleted of SPP, RNF185 or MBRL and localization of the substrate was detected by GFP fluorescence. Bap31 was used as an ER-marker and nuclei were stained with DAPI. Bars, 15 µm

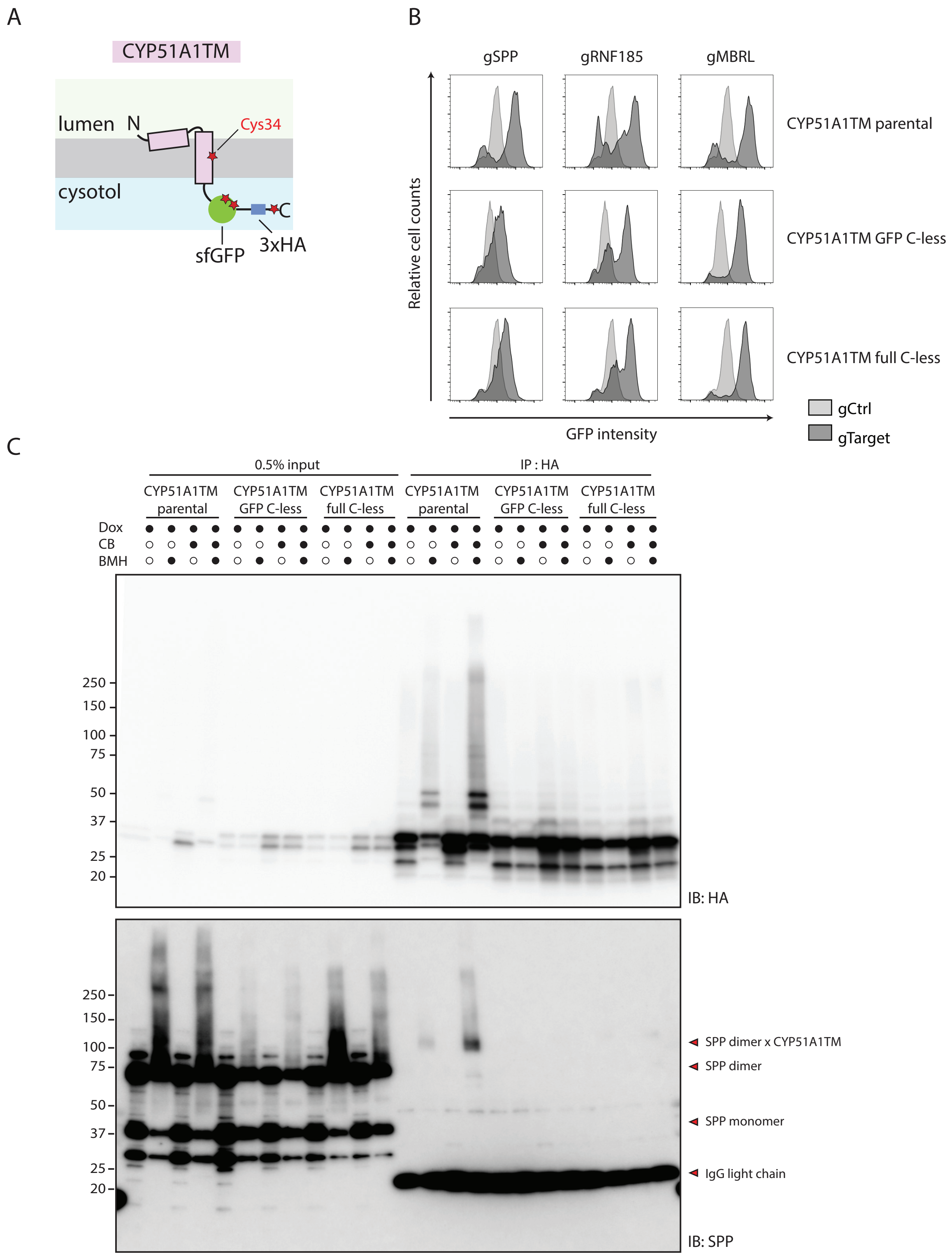

Fig. S2. Identification of cysteine residues on CYP51A1TM required for direct substrate cross-links to endogenous SPP.

A. Schematic depiction of cysteine residues located on CYP51A1TM construct investigated in this study. CYP51A1TM has single cysteine residue within putative TM, followed by three additional cysteines located in the C-terminal sfGFP and 3xHA tags. Cysteine residues are shown with red stars.
B. Flow Cytometry analysis of doxycycline induced expression of wild-type and cysteine mutants (as shown in Fig. S2A.) of CYP51A1TM in HEK293 T-Rex Flip In cells transfected with plasmids encoding gRNA`s targeting indicated genes (dark grey) versus cells transfected with a control plasmid (light grey). Substrate labeled "GFP C-less" represents CYP51A1TM-sfGFP-3HA with cysteines in position 38 and 70 of the sfGFP tag mutated to serines. Substrate labeled "full C-less" has an additional cysteine at the very C-terminus of the CYP51A1TM construct swapped to serine.
C. BMH crosslinking reactions in cells expressing various cysteine mutants of CYP51A1TM. Constructs used in (B) were incubated for 45 minutes with 1mm of cysteine-reactive crosslinker-bismaleimidohex-ane(BMH), quenched with DTT (25mM for 15 minutes), and the lysates generated were immunoprecipi-tated using anti-HA beads and analysed by immunoblotting. Red arrow represents direct substrate crosslinks to endogenous SPP.

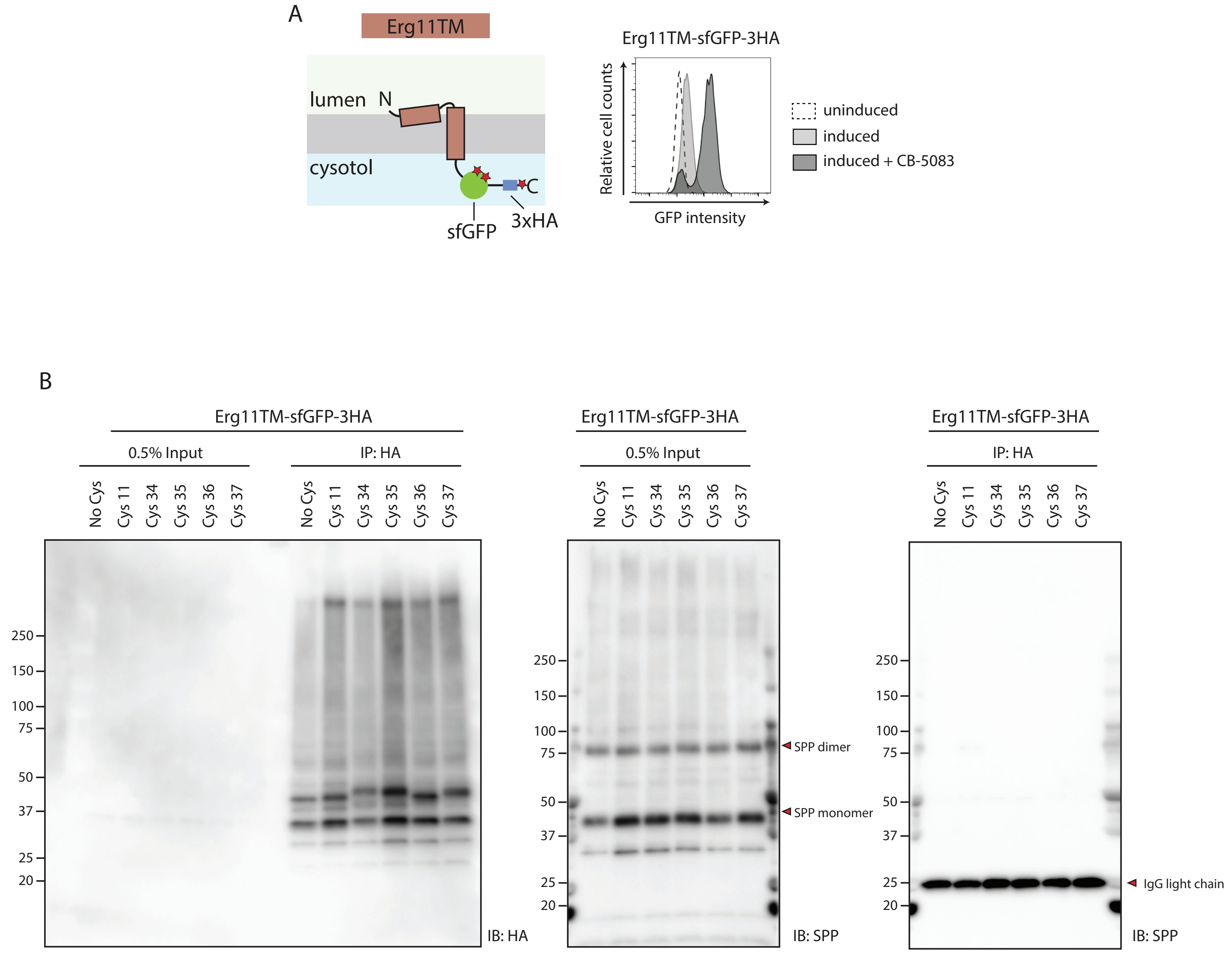

Fig. S3. Topologically similar model substrate of MARCHF6 ERAD complex, Erg11TM, and its cysteine mutants do not crosslink to endogenous SPP.

A. Schematic depiction of Erg11TM (left) and validation of Erg11TM as an ERAD substrate (right). Expression levels upon doxycycline induced expression in presence or absence of p97 inhibitor, CB-5083 (4h, right panel, light grey versus dark grey respectively). Uninduced cells were used as a control (right panel, dotted line). Red stars represent cysteine residues present in the model construct.

B. Assessment of Erg11TM crosslinking pattern. Wild-type Erg11TM as well as cysteine mutants of the Erg11TM construct under doxyxycline inducible promoter were stably expressed in HEK293 TRex Flip In cells and incubated with 1mM BMH for 45 minutes. Cys11 is introduced in the AH region and Cys34-37 in the TM region of the substrate. The lysates after quenching with 25mM DTT were immunoprecipitated (anti-HA) and analysed by SDS-PAGE, probing with HA antibody (left) as well as SPP-specific antibody (both Input and IP shown).

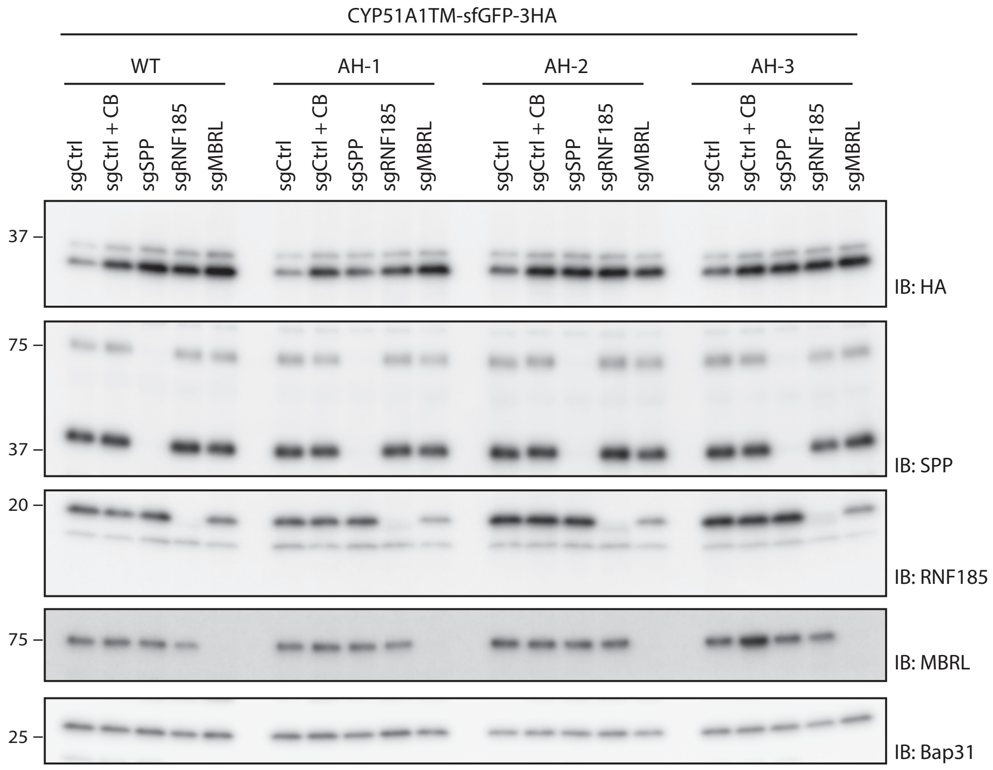

Fig. S4. SPP recognizes AH of CYP51A1TM.

A. Same as Figure 5E but comparing WT CYP51A1TM construct against AH truncations AH-1, AH-2, and AH-3 (as seen in Figure 5B, C, D).

Journal of Cell Science • Supplementary information

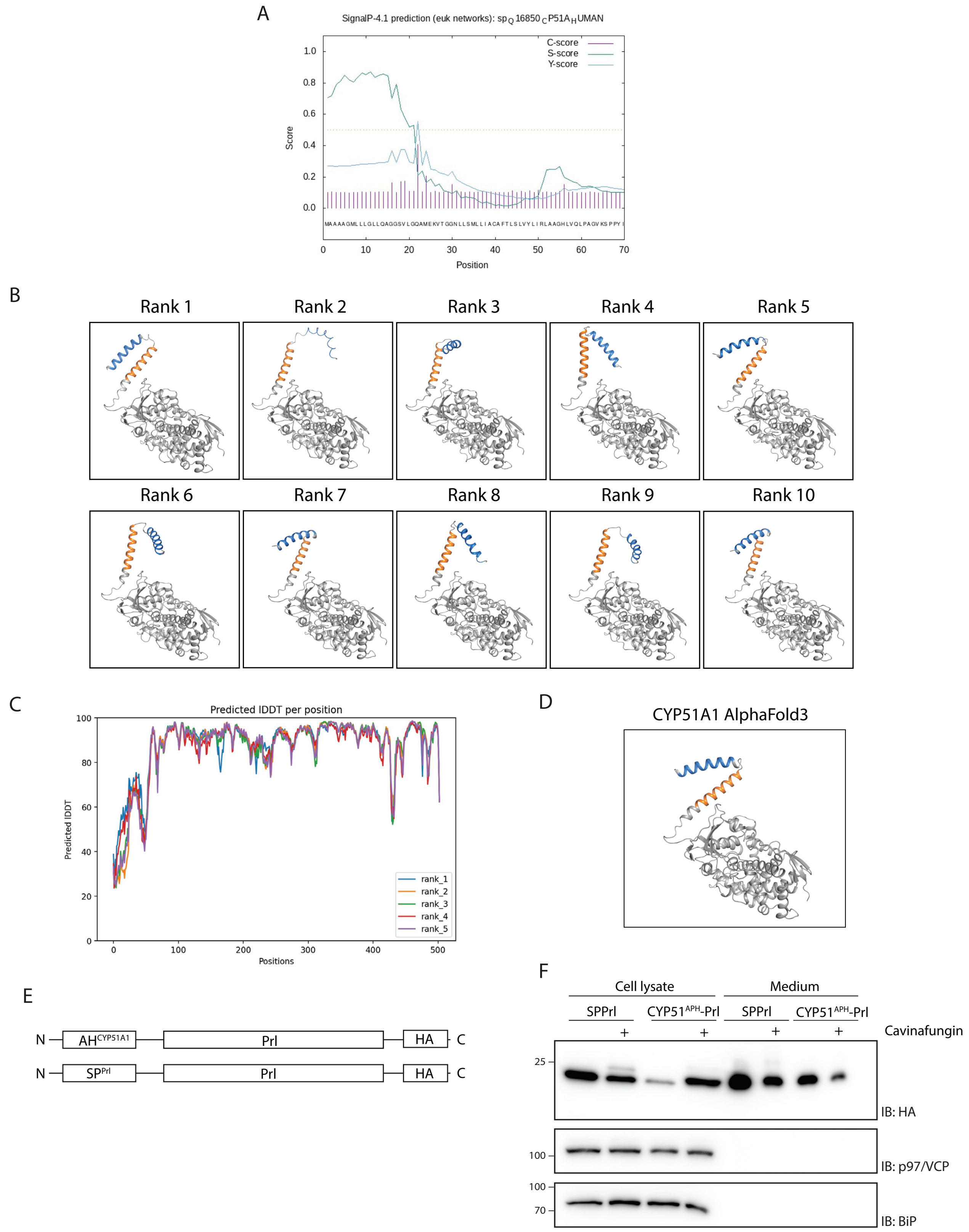

Fig. S5. Conformational dynamics of AH of CYP51A1 allows it to adopt a type II topology.

A. Graphical output of SignalP 4.1 scores for the first 70 amino acid residues of CYP51A1. Predicted cleavage sites are shown as peaks in the Y-score

B. AlphaFold2 models of CYP51A1 produced using MSA subsampling, and coloured as per Figure S5A. AlphaFold2 calculations were done utilising the MSA subsampling approach to generate additional conformational sampling (Del Alamo et al., 2022). Top 10 scoring results are shown, revealing large conformational dynamics of the N-terminus of the protein. Proteins are aligned based on the cytosolic domain

C. AlphaFold per-residue confidence score (pLDDT) plotted for the top 5 ranked models from panel B.

D. AlphaFold3 model of CYP51A1, coloured and aligned as per panels B.

E. Schematic depiction of the model constructs used in secretion assay. Signal sequence of bovine prolactin was either left intact (SPPrl) or substituted with N-terminal AH of CYP51A1 (CYP51A1-APH-Prl). Both constructs have C-terminal HA tag for detection by Immunoblot.

F. Western blot analysis depicting the secretion assay for bovine prolactin and CYP51A1 AH-prolactin fusion, untreated or treated with signal peptidase inhibitor, cavinafungin (2 μM, 16h), to inhibit signal peptide processing. p97/VCP and BiP are used as controls.

Journal of Cell Science • Supplementary information

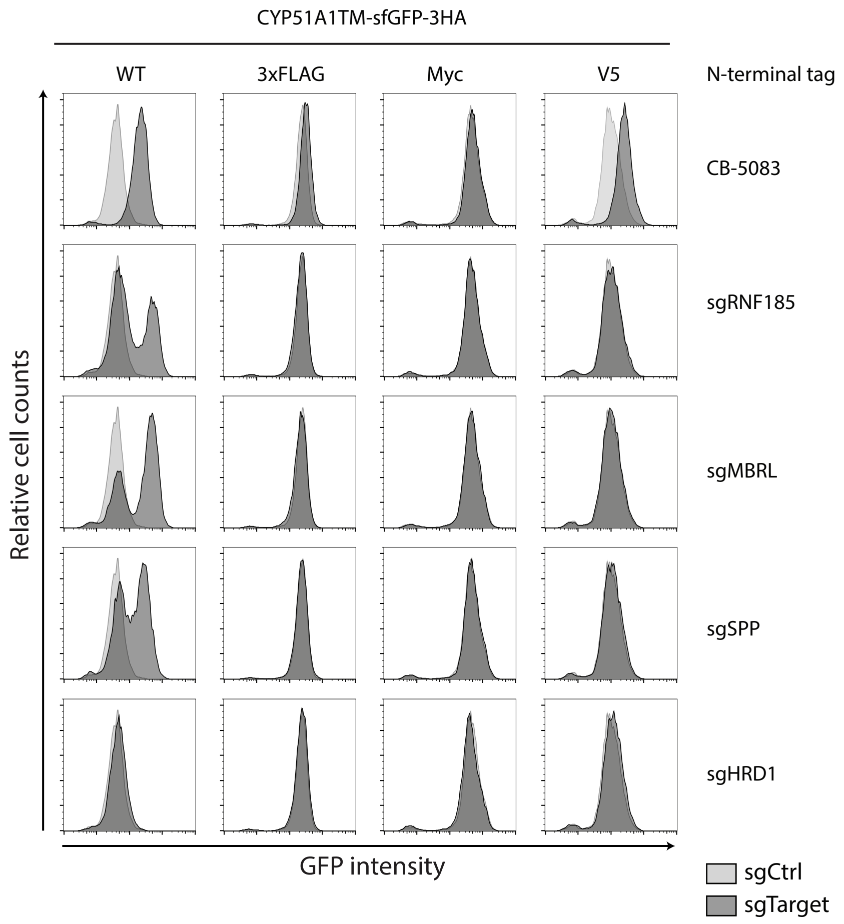

Fig. S6. N-terminal tags of CYP51A1 inhibit their SPP-dependent quality control.

CYP51A1TM was N-terminally tagged with either FLAG, Myc or V5 tags and the stability of the model protein was assessed by Flow Cytometry (based on GFP fluorescence) in presence of either p97 inhibitor CB-5083 or gRNA's targeting the genes indicated (dark grey) versus gRNA control (light grey).

Journal of Cell Science • Supplementary information

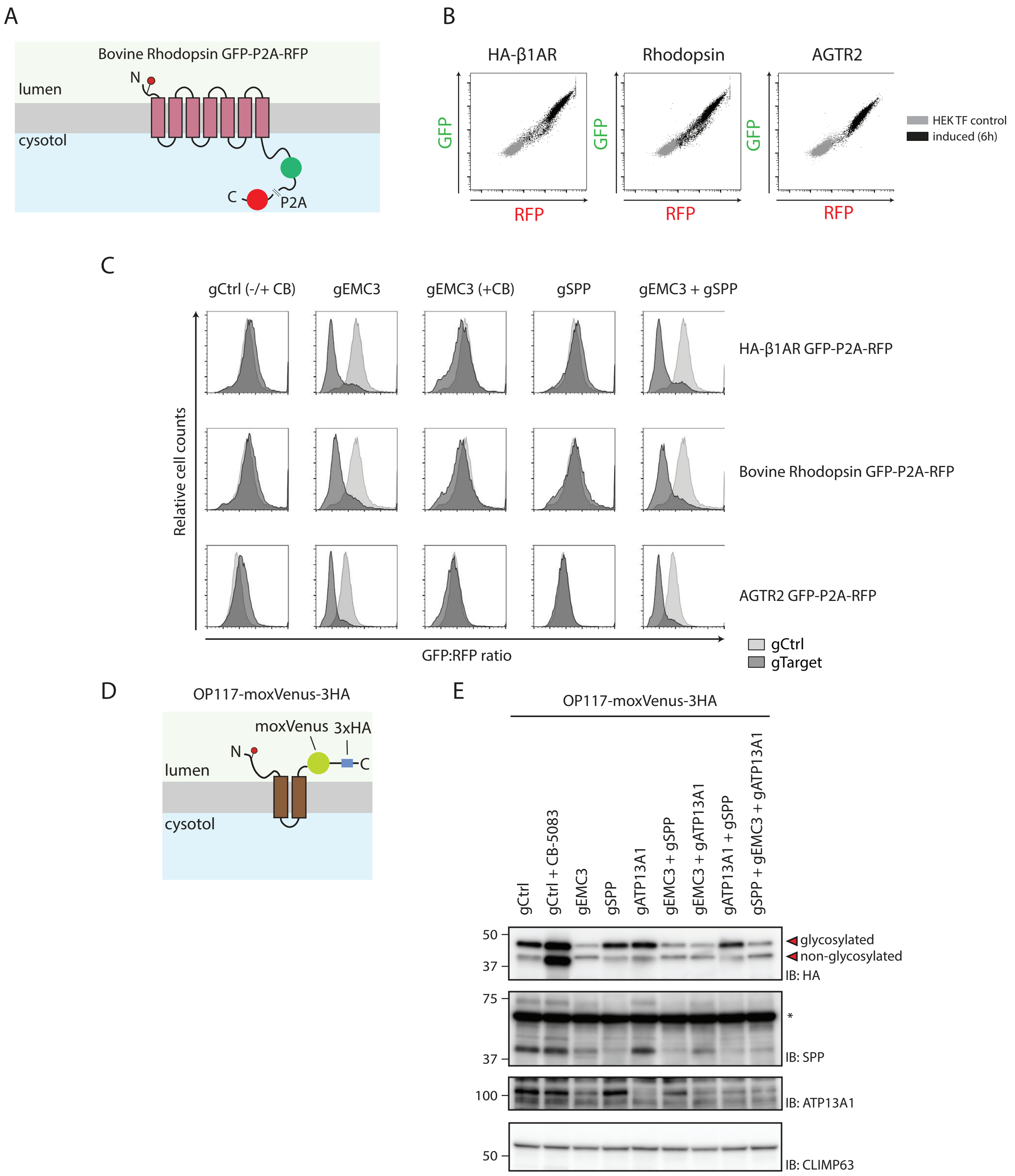

Fig. S7. SPP does not play a role in topology surveillance of G-protein coupled receptors (GPCRs).

A. Schematic representation of the GPCR used in this study, bovine rhodopsin. The construct was appended to C-terminal GFP and RFP tags separated by a viral P2A sequence. This allowed each translation cycle to generate two distinct products – a GFP tagged protein of interest and an RFP that serves as an internal translational control.
B. Scatter plots of the indicated GPCRs, each expressed from a doxycycline inducible promoter in HEK293 TF stable cell lines. Cells were incubated with doxycycline for 6h to prevent oversaturation of the ERAD machinery. Expression profiles of GPCRs of interest (dark grey) were compared to control HEK293 TRex Flip In cells (light grey).
C. Ratiometric analysis of GPCR expression upon transfection with plasmids encoding for the indicated gRNA's (dark grey) or control gRNA (light grey). Where indicated, cells were further treated with p97 inhibitor, CB-5083.
D. Schematic representation of the truncated version of bovine rhodopsin, OP117. The construct encompasses two TM domains and is appended to the C-terminal moxVenus and 3xHA tags.
E. Assessment of the steady-state levels of OP117, shown in (D), upon transfection with indicated gRNA's and/or treatment with p97 inhibitor, CB-5083 (4h). Red arrows represent two populations of the substrate – inserted in the right orientation (glycosylated) and inverted orientation (non-glycosylated). Asterisk represents a non-specific band.

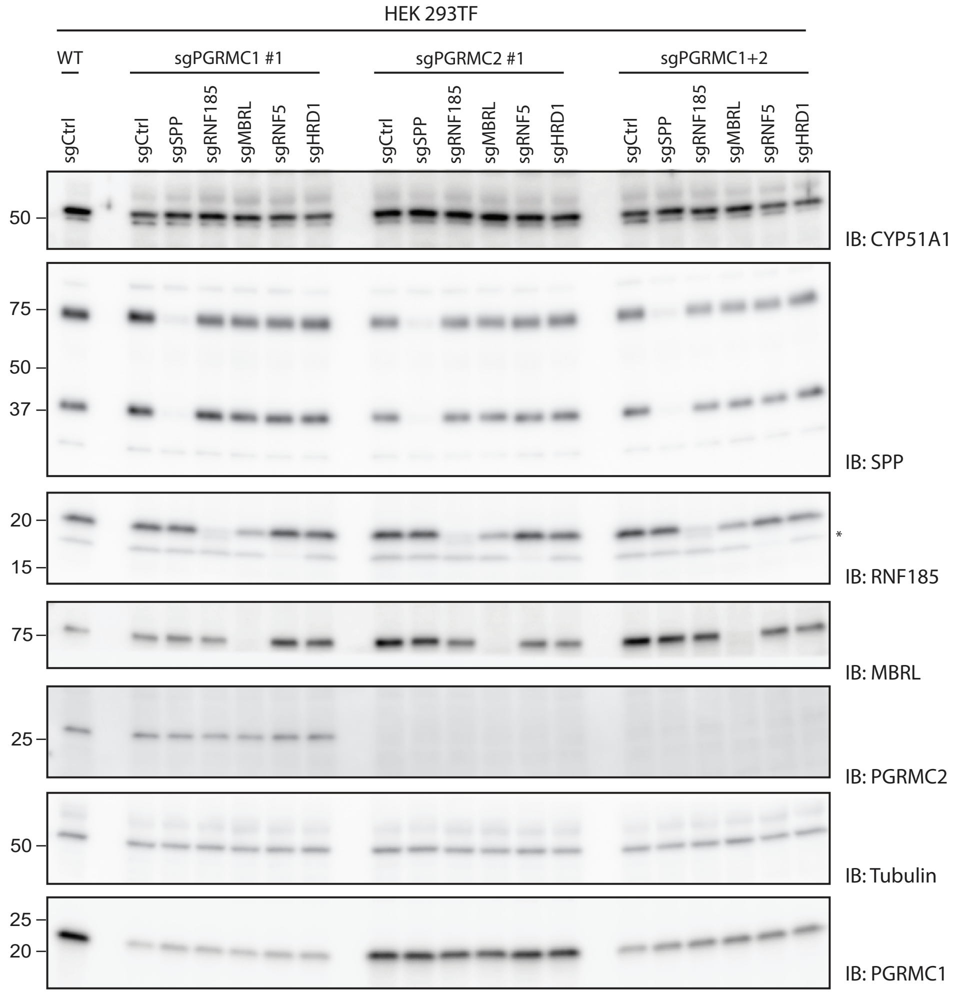

Fig. S8. PGRMC1/2 do not play a role in SPP-mediated ERAD of CYP51A1

Immunoblot assessing steady-state levels of endogenous CYP51A1 in cells carrying individual (PGRMC1, PGRMC2) or simultaneous (PGRMC1+2) depletion of the PGRMC's transfected with a panel of gRNA's against target ERAD factors indicated. Asterisk represents RNF5-specific band.

Journal of Cell Science • Supplementary information

Table S1. Sequences of CYP450-derived model substrates used in this study.

All constructs were designed to terminate on a helix breaking residue, proline. Model substrates are grouped by their correspondence to the indicated family of CYP450 enzymes. Putative TMs of each protein, identified using AFTM database (available at: https://conglab.swmed.edu/AFTM/) are underlined.

Family of CYP450	Protein	Model TM substrate	Protein sequence (putative TM based on AFTM database)
**CYP51**	CYP51A1	**CYP51A1TM (1-61)**	MLLLGLLQAGGSVLGQAMEKVTGGNLLSMLLIACAFTLSLVYLIRLAAGHLVQLPAGVKSP
**CYP1**	CYP1A1	**CYP1A1TM (1-59)**	MLFPISMSATEFLLASVIFCLVFWVIRASRPQVPKGLKNPPGPWGWPLIGHMLTLGKNP
**CYP2**	CYP2A6	**CYP2A6TM (1-67)**	MLASGMLLVALLVCLTVMVLMSVWQQRKSKGKLPPGPTPLPFIGNYLQLNTEQMYNSLMKISERYGP
	CYP2U1	**CYP2U1TM (1-60)**	MSSPGPSQPPAEDPPWPARLLRAPLGLLRLDPSGGALLLCGLVALLGWSWLRRRRARGIP
**CYP3**	CYP3A4	**CYP3A4TM (1-45)**	MALIPDLAMETWLLLAVSLVLLYLYGTHSHGLFKKLGIPGPTPLP
**CYP4**	CYP4A11	**CYP4A11TM (1-54)**	MSVSVLSPSRLLGDVSGILQAASLLILLLLLIKAVQLYLHRQWLLKALQQFPCP
	CYP4F2	**CYP4F2TM (1-69)**	MSQLSLSWLGLWPVAASPWLLLLLVGASWLLAHVLAWTYAFYDNCRRLRCFPQPPRRNWFWGHQGMVNP
	CYP4F12	**CYP4F12TM (1-55)**	MSLLSLPWLGLRPVATSPWLLLLLVVGSWLLARILAWTYAFYNNCRRLQCFPQPP
	CYP4V2	**CYP4V2TM (1-58)**	MAGLWLGLVWQKLLLWGAASALSLAGASLVLSLLQRVASYARKWQQMRPIPTVARAYP
**CYP5**	CYP5A1	**CYP5A1TM (1-51)**	MEALGFLKLEVNGPMVTVALSVALLALLKWYSTSAFSRLEKLGLRHPKPSP
**CYP7**	CYP7B1	**CYP7B1TM (1-66)**	MAGEVSAATGRFSLERLGLPGLALAAALLLLALCLLVRRTRRPGEPPLIKGWLPYLGVVLNLRKDP
**CYP8**	CYP8A1	**CYP8A1TM (1-38)**	MAWAALLGLLAALLLLLLLSRRRTRRPGEPPLDLGSIP
	CYP8B1	**CYP8B1TM (1-41)**	MVLWGPVLGALLVVIAGYLCLPGMLRQRRPWEPPLDKGTVP
**CYP17**	CYP17A1	**CYP17A1TM(1-62)**	MWELVALLLLTLAYLFWPKRRCPGAKYPKSLLSLPLVGSLPFLPRHGHMHNNFFKLQKKYGP
**CYP19**	CYP19A1	**CYP19A1TM (1-58)**	MVLEMLNPIHYNITSIVPEAMPAATMPVLLLTGLFLLVWNYEGTSSIPGPGYCMGIGP
**CYP21**	CYP21A2	**CYP21A2TM(1-57)**	MLLLGLLLLPLLAGARLLWNWWKLRSLHLPPLAPGFLHLLQPDLPIYLLGLTQKFGP
**CYP26**	CYP26A1	**CYP26A1TM(1-52)**	MGLPALLASALCTFVLPLLLFLAAIKLWDLYCVSGRDRSCALPLPPGTMGFP
	CYP26B1	**CYP26B1TM(1-57)**	MLFEGLDLVSALATLAACLVSVTLLLAVSQQLWQLRWAATRDKSCKLPIPKGSMGFP
	CYP26C1	**CYP26C1TM (1-57)**	MFPWGLSCLSVLGAAGTALLCAGLLLSLAQHLWTLRWMLSRDRASTLPLPKGSMGWP
**CYP39**	CYP39A1	**CYP39A1TM (1-62)**	MELISPTVIIILGCLALFLLLQRKNLRRPPCIKGWIPWIGVGFEFGKAPLEFIEKARIKYGP
**CYP46**	CYP46A1	**CYP46A1TM (1-47)**	MSPGLLLLGSAVLLAFGLCCTFVHRARSRYEHIPGPPRPSFLLGHLP

Table S2. List of antibodies used in this study.

Antigen	Organism	Dilution	Source	Catalog number
**Primary**				
HA (3F10)	Rat	1 to 2000	Roche	11867423001; RRID:AB_390918
Tubulin	Rat	1 to 1000	Santa Cruz	sc-53030; RRID:AB_2272440
RNF185	Rabbit	1 to 5000	Abcam	ab181999; RRID:AB_2922962
MBRL	Rabbit	1 to 1000	Atlas Antibodies	HPA042669; RRID:AB_10794916
FLAG	Mouse	1 to 2000	Sigma	F1804; RRID:AB_262044
GAPDH	Mouse	1 to 5000	ProteinTech	60004-1-Ig; RRID:AB_2107436
CYP51A1	Rabbit	1 to 2500	ProteinTech	13431-1-AP; RRID:AB_2088571
TMUB2	Rabbit	1 to 1000	ProteinTech	28044-1-AP; RRID:AB_2881045
SPP	Rabbit	1 to 5000	ProteinTech	20416-1-AP
TMUB1	Rabbit	1 to 5000	Abcam	ab180586; RRID:AB_2922961
HRD1	Rabbit	1 to 1000	Cell Signaling	14773; RRID:AB_2798607
GFP	Mouse	1 to 1000	Sigma	11814460001; RRID:AB_390913
Actin	Mouse	1 to 1000	Sigma	A2228; RRID:AB_476697
Calnexin	Rabbit	1 to 2000	Abcam	ab92573; , RRID:AB_10563673
CLIMP63	Rabbit	1 to 1000	ProteinTech	16686-1-AP; RRID:AB_2276275
Ubiquitin	Rabbit	1 to 5000	Cell Signaling	43124; RRID:AB_2180538
Bap31	Mouse	1 to 1000	Enzo	ALX-804-601-C100; RRID:AB_2050797
Sel1L	Rabbit	1 to 1000	Abcam	ab78298; RRID:AB_2285813
ATP13A1	Rabbit	1 to 1000	ProteinTech	16244-1-AP
SQLE	Rabbit	1 to 1000	ProteinTech	12544-1-AP; RRID:AB_2195888
p97/VCP	Rabbit	1 to 20 000	N/A	Gift from Marius Lemberg
BiP	Rabbit	1 to 500	Abcam	ab21685; RRID:AB_2119834
PGRMC1	Rabbit	1 to 1000	ProteinTech	12990-1-AP; RRID:AB_2164342
PGRMC2	Mouse	1 to 1000	ProteinTech	60249-1-Ig; RRID:AB_2881370
**Secondary**				
Rabbit IgG	Mouse	1 to 10 000	Jackson ImmunoResearch	211-032-171; RRID:AB_2339149
Rat IgG	Goat	1 to 10 000	Jackson ImmunoResearch	112-035-175; RRID:AB_2338140
Mouse IgG	Goat	1 to 10 000	Jackson ImmunoResearch	115-035-174; RRID:AB_2338512
Alexa Fluor Mouse IgG 568	Goat	1 to 400	Thermo Fisher Scientific	A11004; RRID:AB_2534072

Journal of Cell Science • Supplementary information

Figure 1D

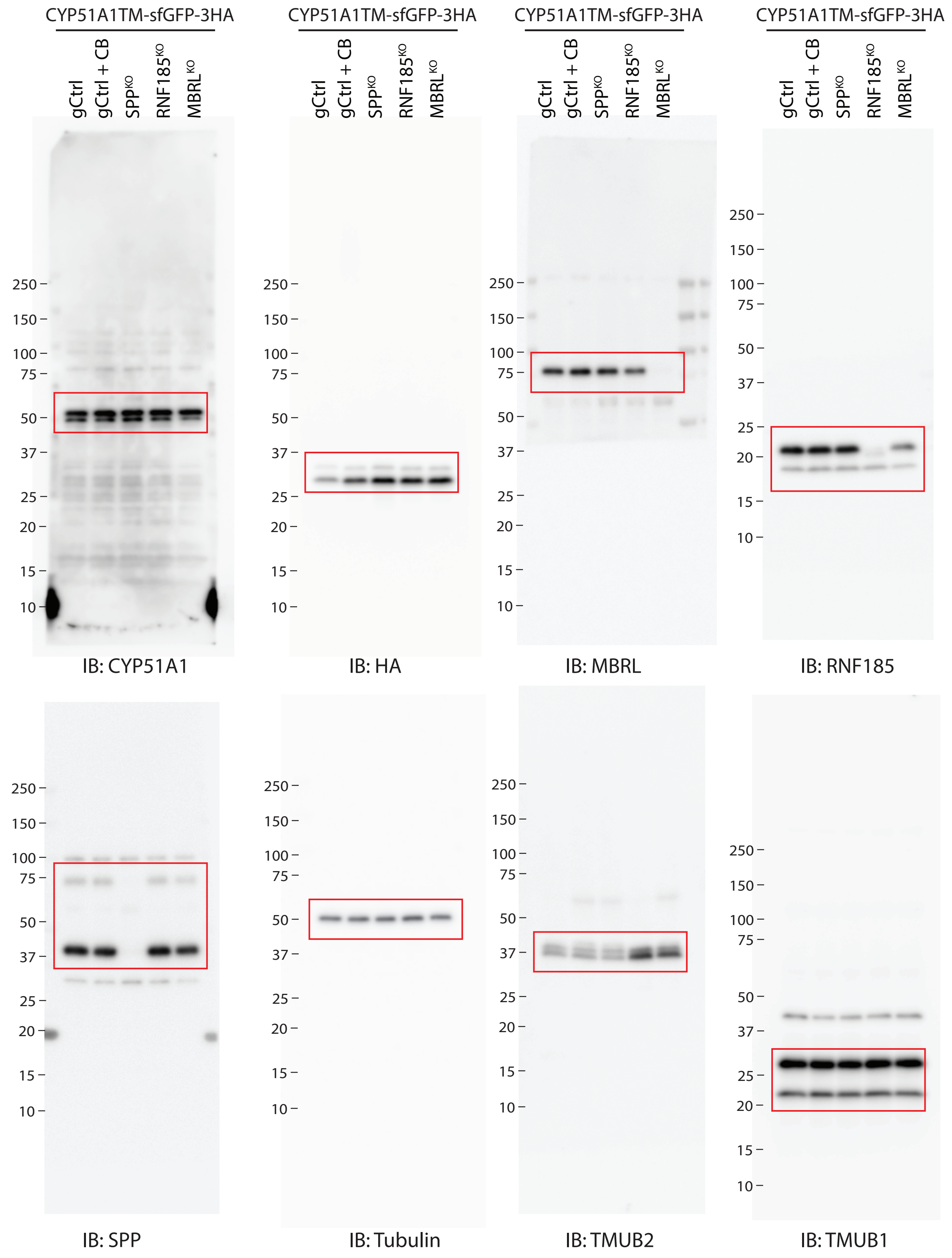

Fig. S9. Blot Transparency Figures - Full western blot images of Figure 1D.

Journal of Cell Science • Supplementary information

Figure 1E

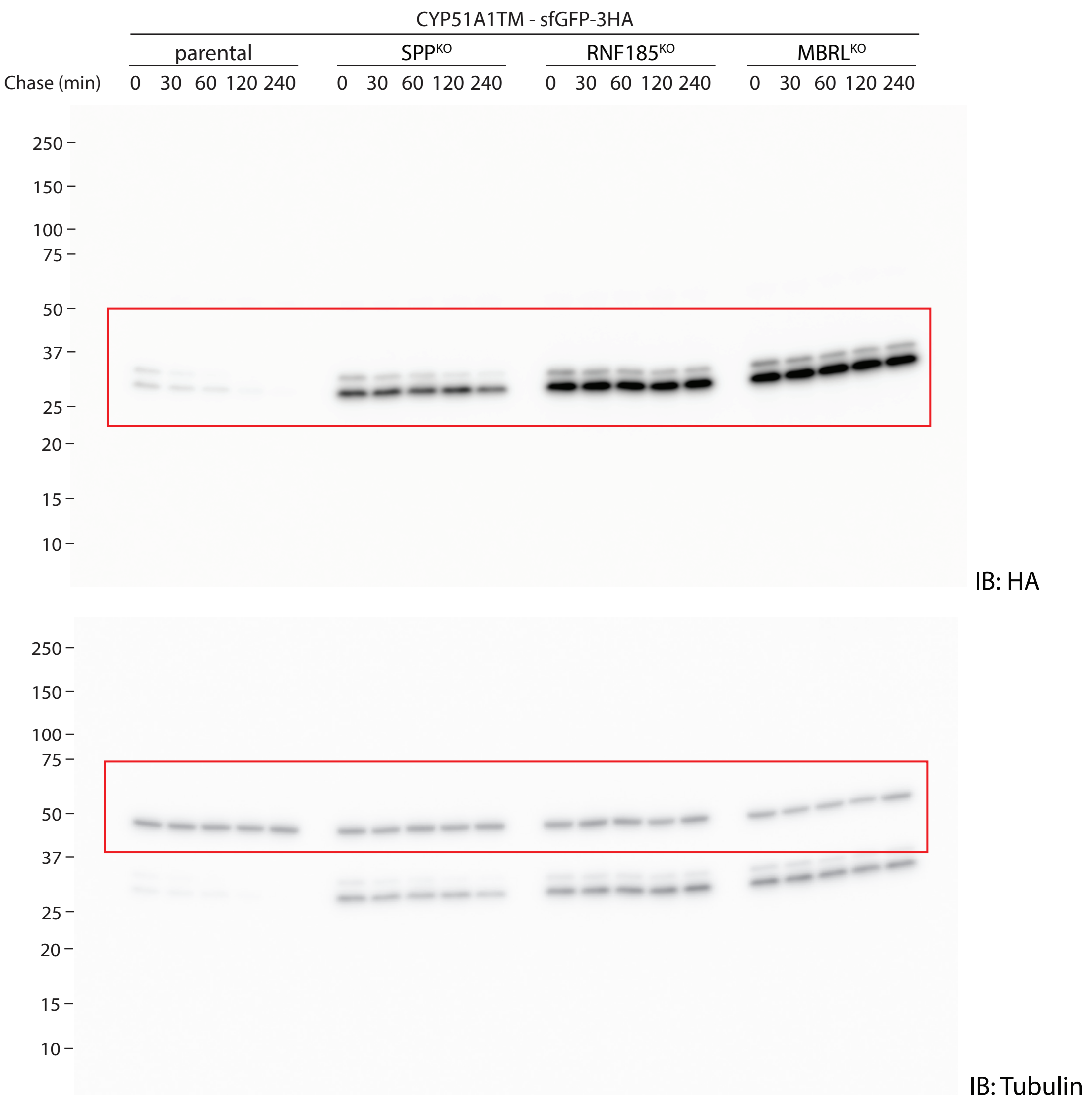

Fig. S10. Blot Transparency Figures - Full western blot images of Fig. 1E.

Journal of Cell Science • Supplementary information

Figure 2B

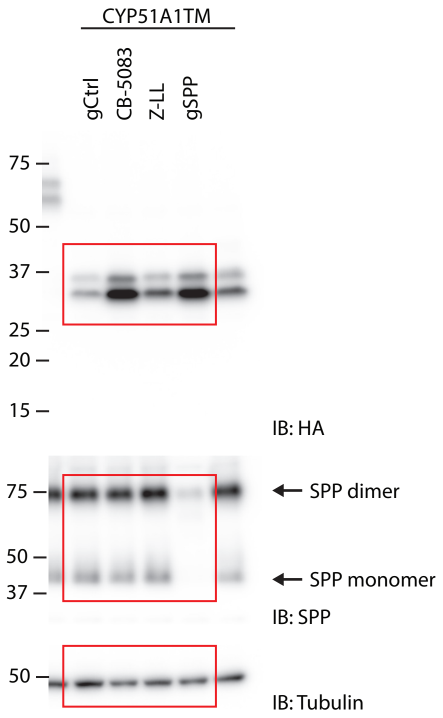

Figure 2C

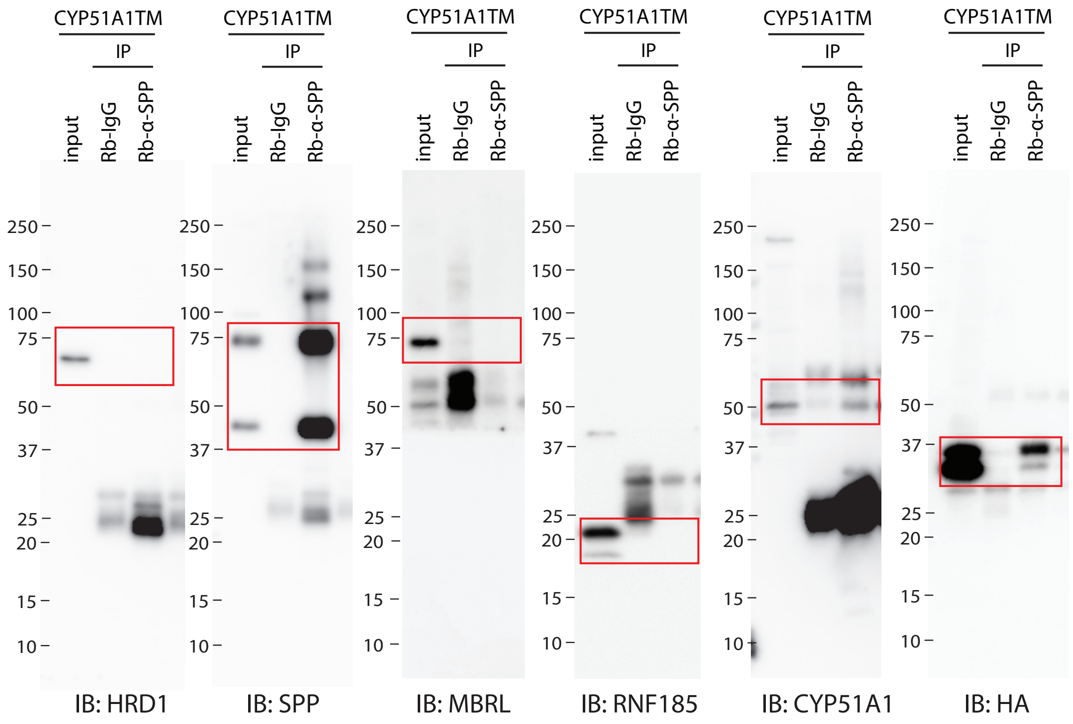

Figure 2D

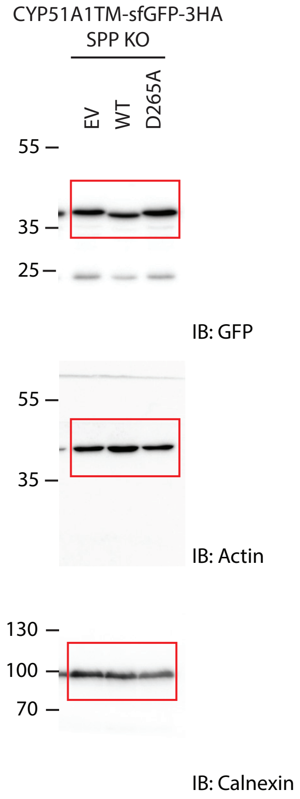

Figure 2E

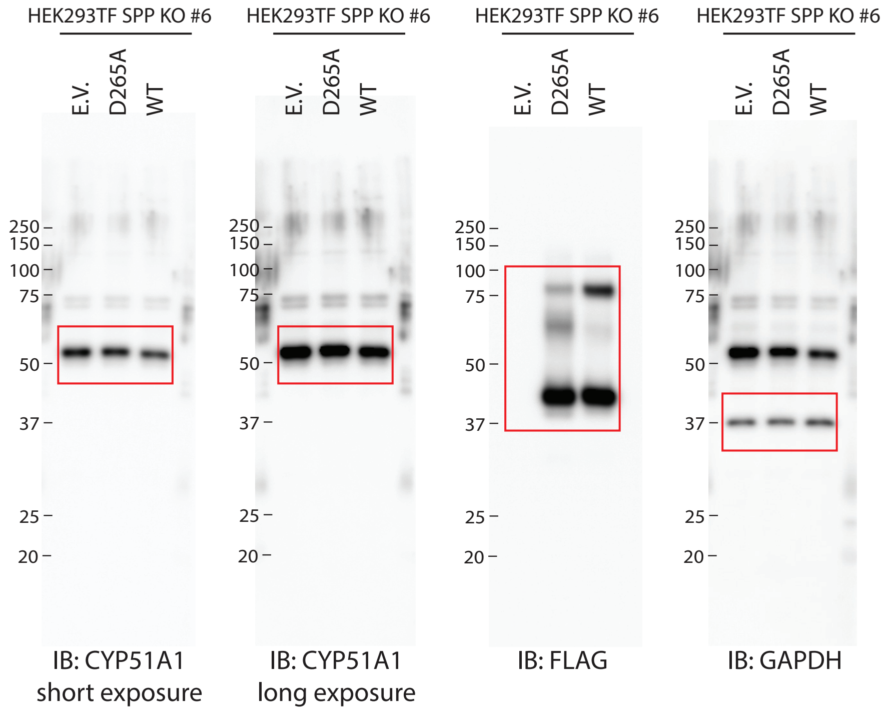

Fig. S11. Blot Transparency Figures - Full western blot images of Fig. 2A-E.

Figure 2F

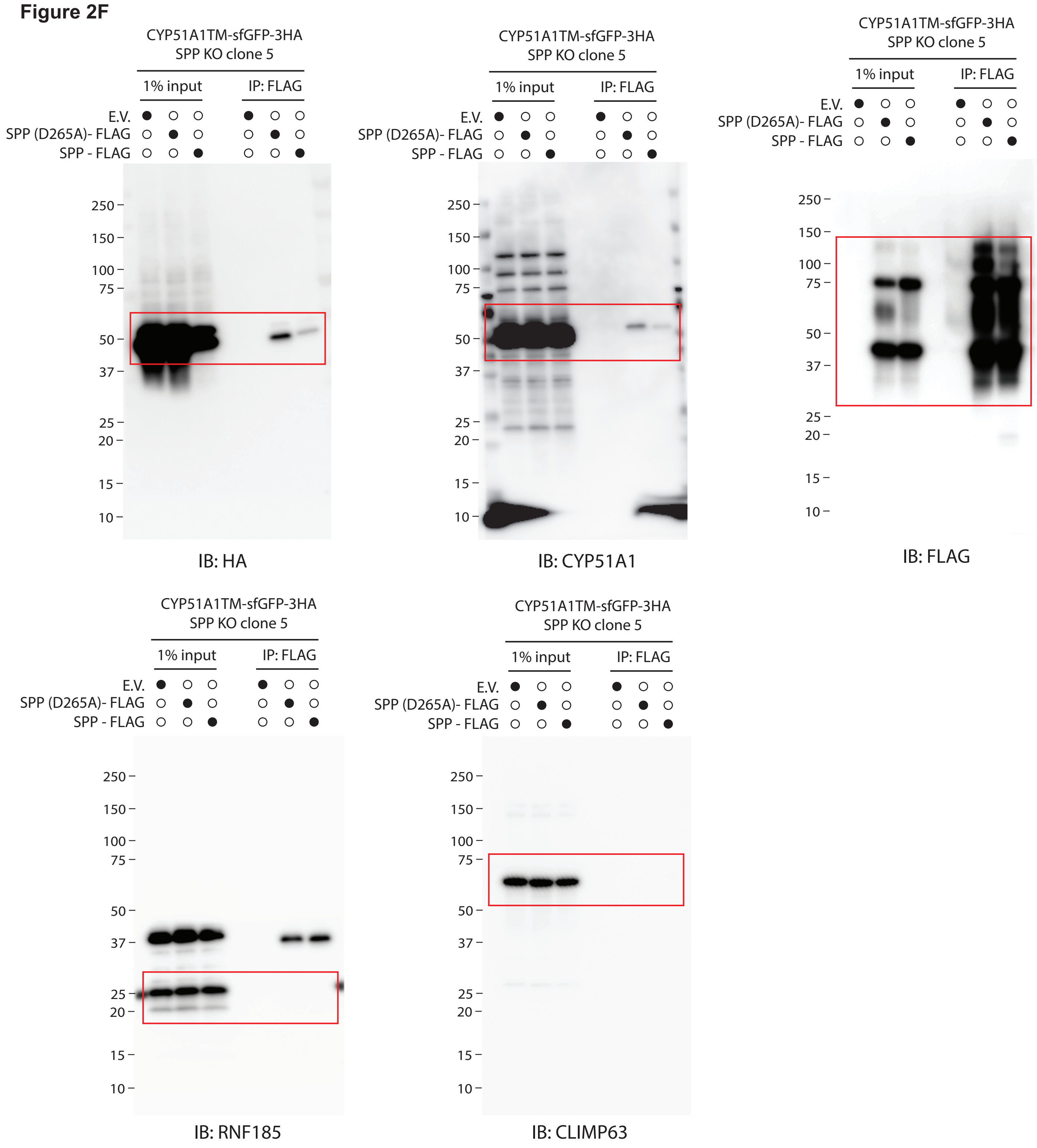

Fig. S12. Blot Transparency Figures - Full western blot images of Fig. 2F.

Journal of Cell Science • Supplementary information

Figure 2G

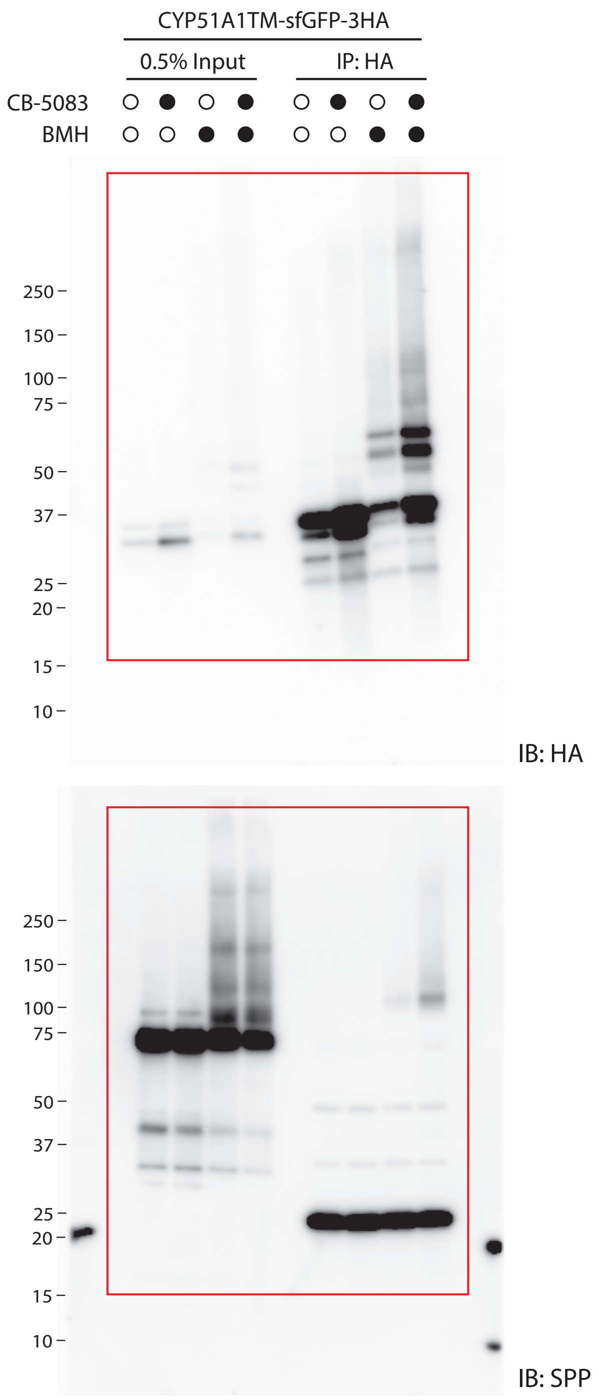

Figure 3A

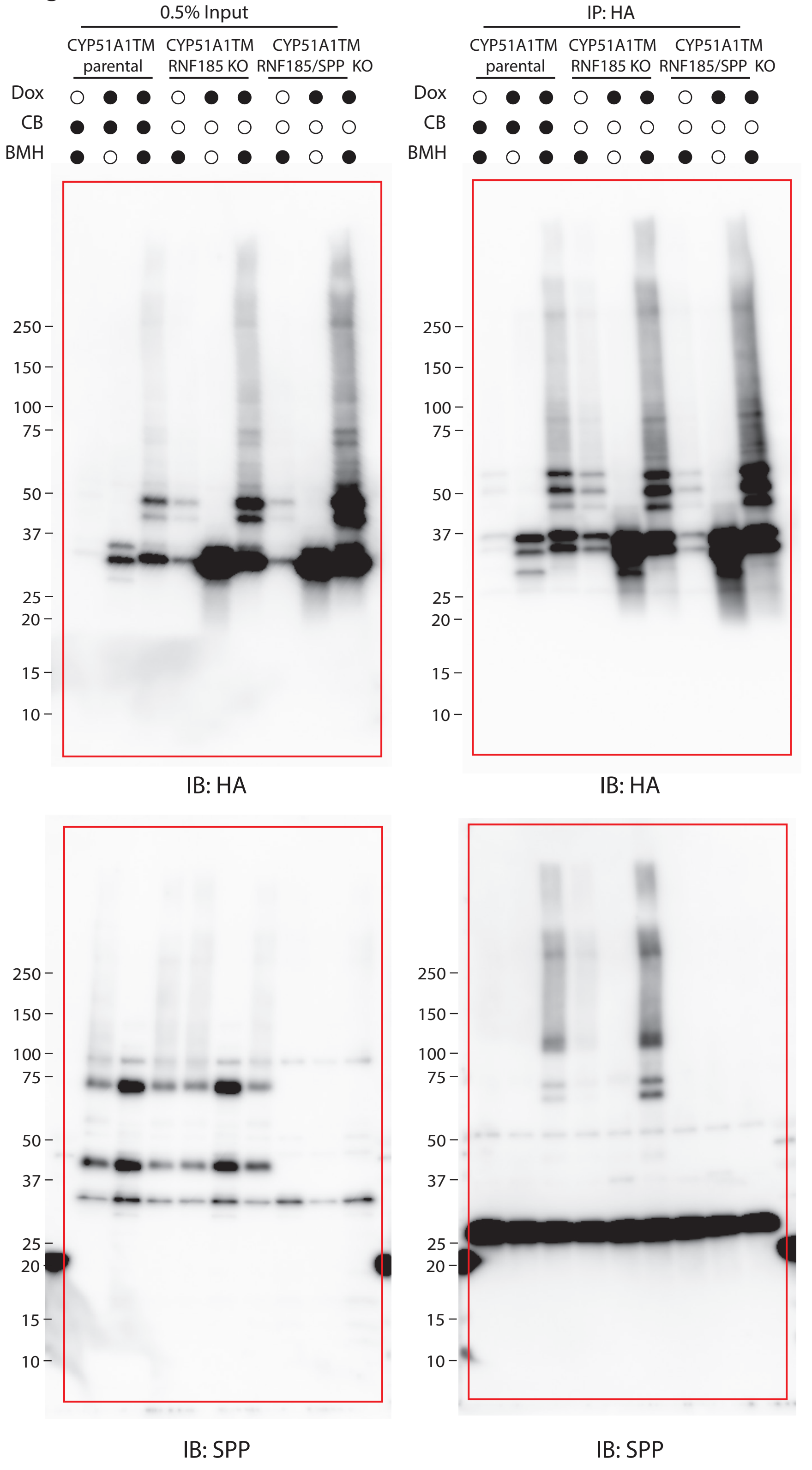

Fig. S13. Blot Transparency Figures - Full western blot images of Fig. 2G and Fig. 3A.

Journal of Cell Science • Supplementary information

Figure 3B

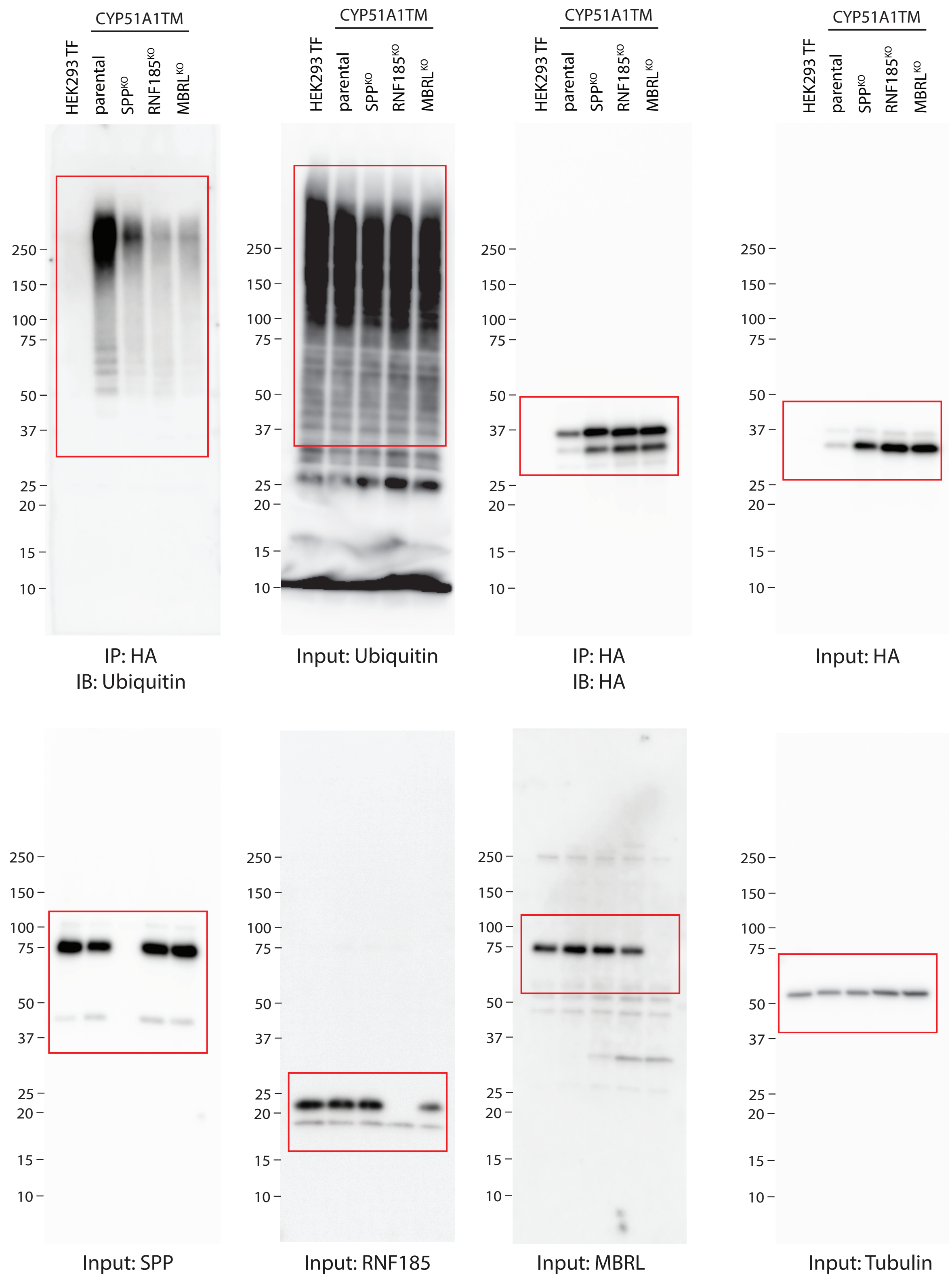

Fig. S14. Blot Transparency Figures - Full western blot images of Fig. 3B.

Journal of Cell Science • Supplementary information

Figure 4C

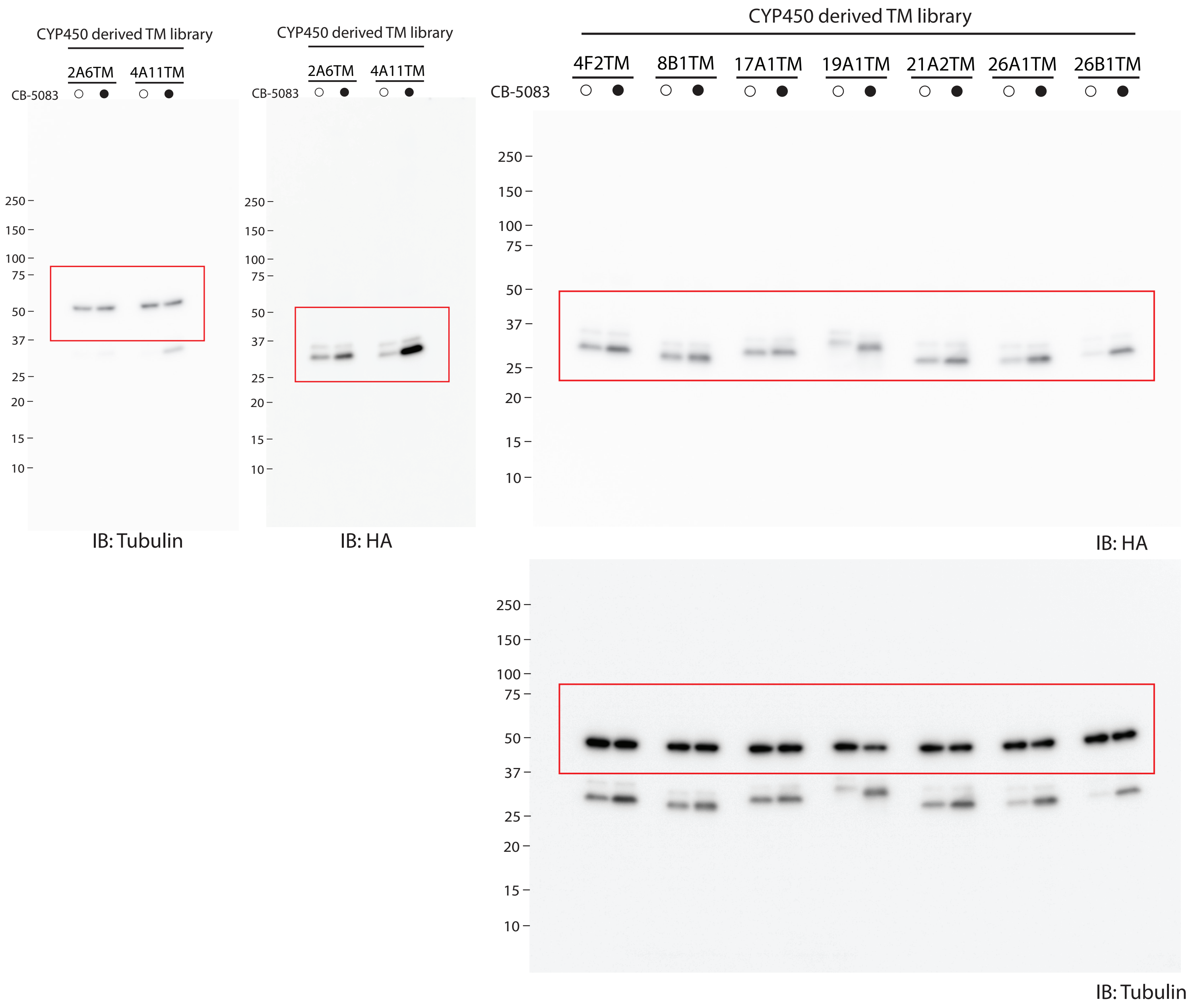

Fig. S15. Blot Transparency Figures - Full western blot images of Fig. 4C.

Journal of Cell Science • Supplementary information

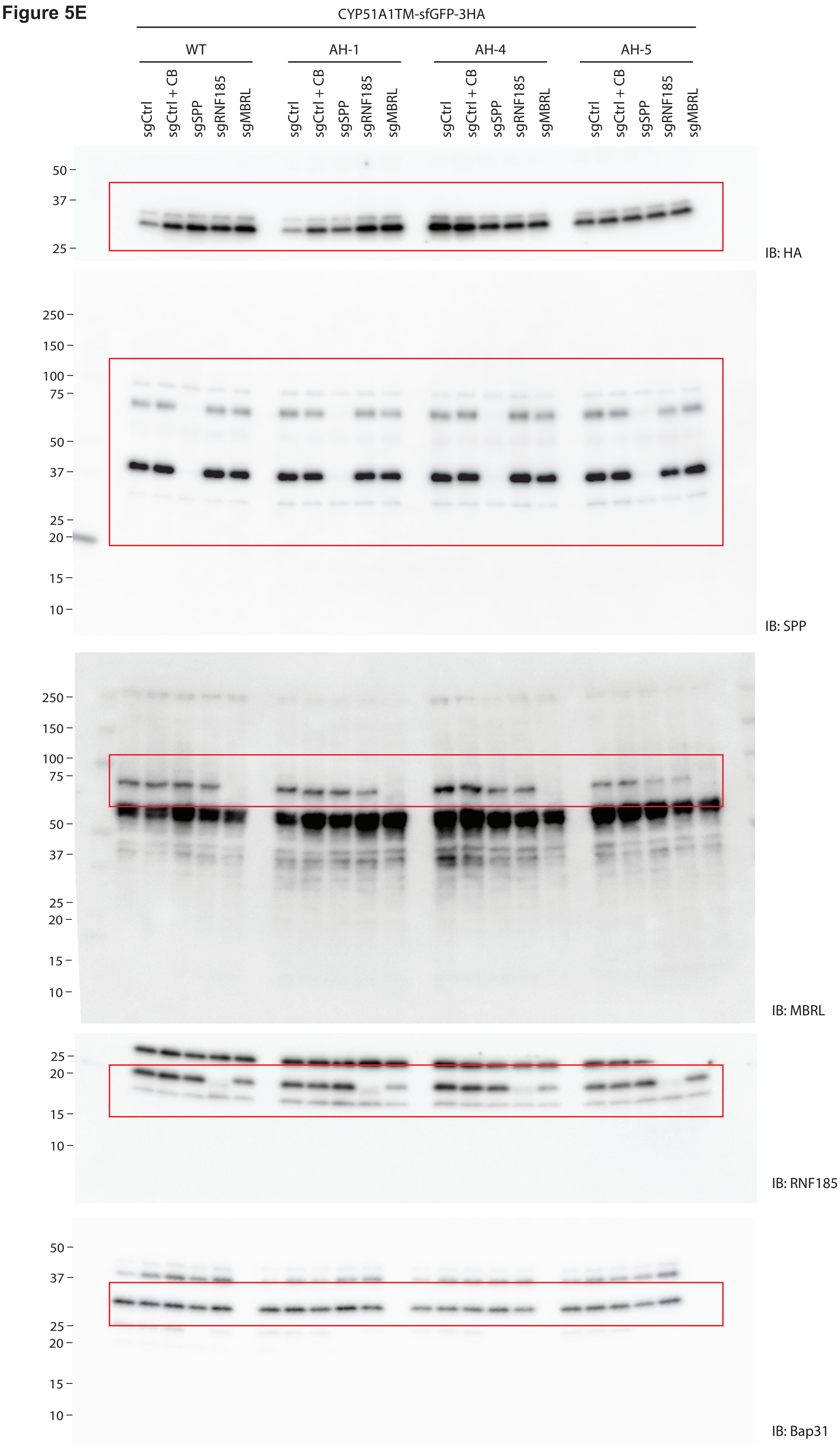

Fig. S16. Blot Transparency Figures - Full western blot images of Fig. 5E.

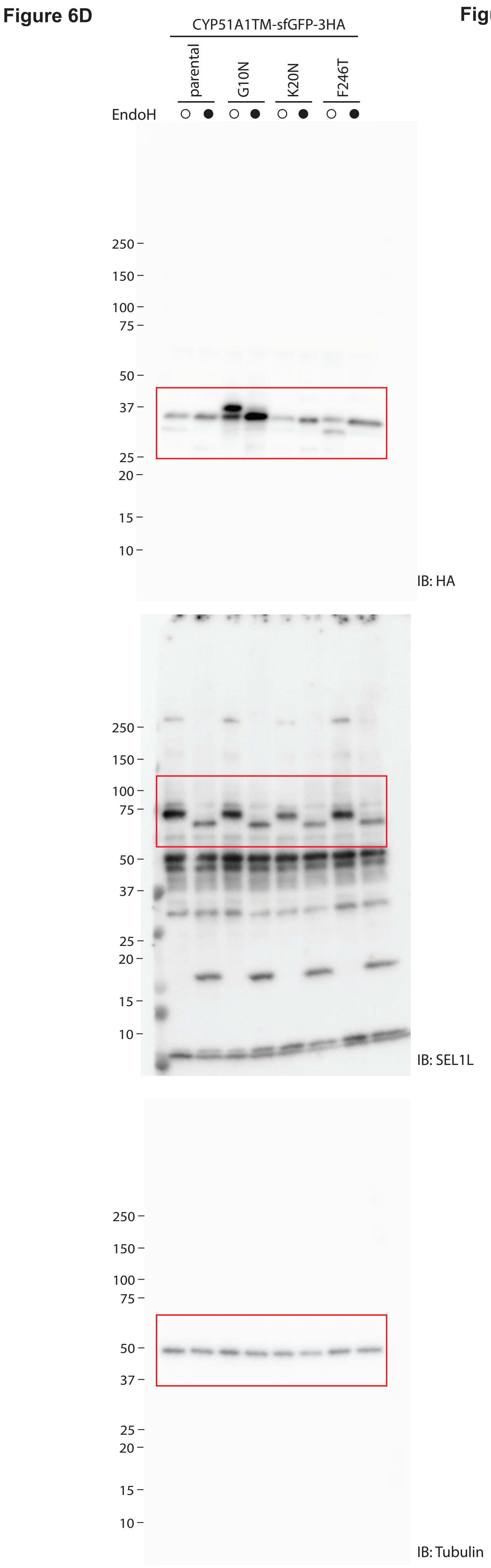

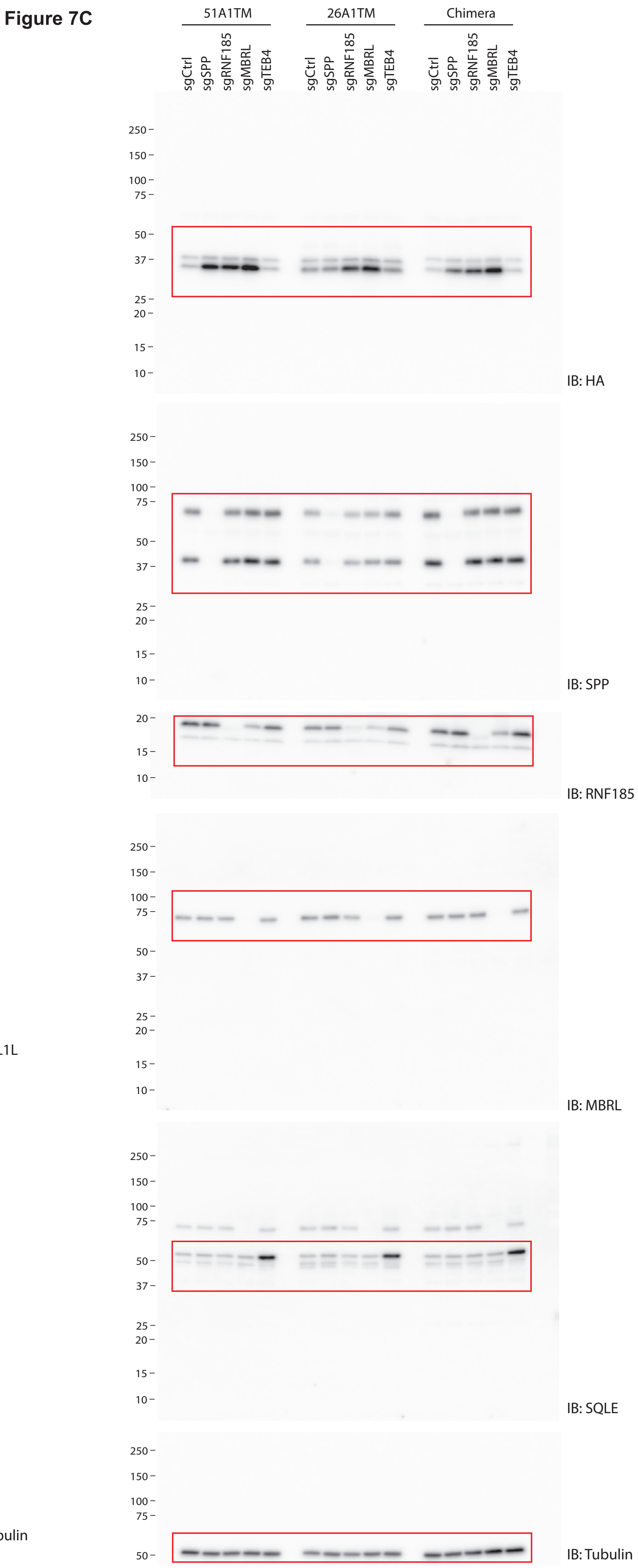

Fig. S17. Blot Transparency Figures - Full western blot images of Figure 6D and Fig. 7C.

Journal of Cell Science • Supplementary information

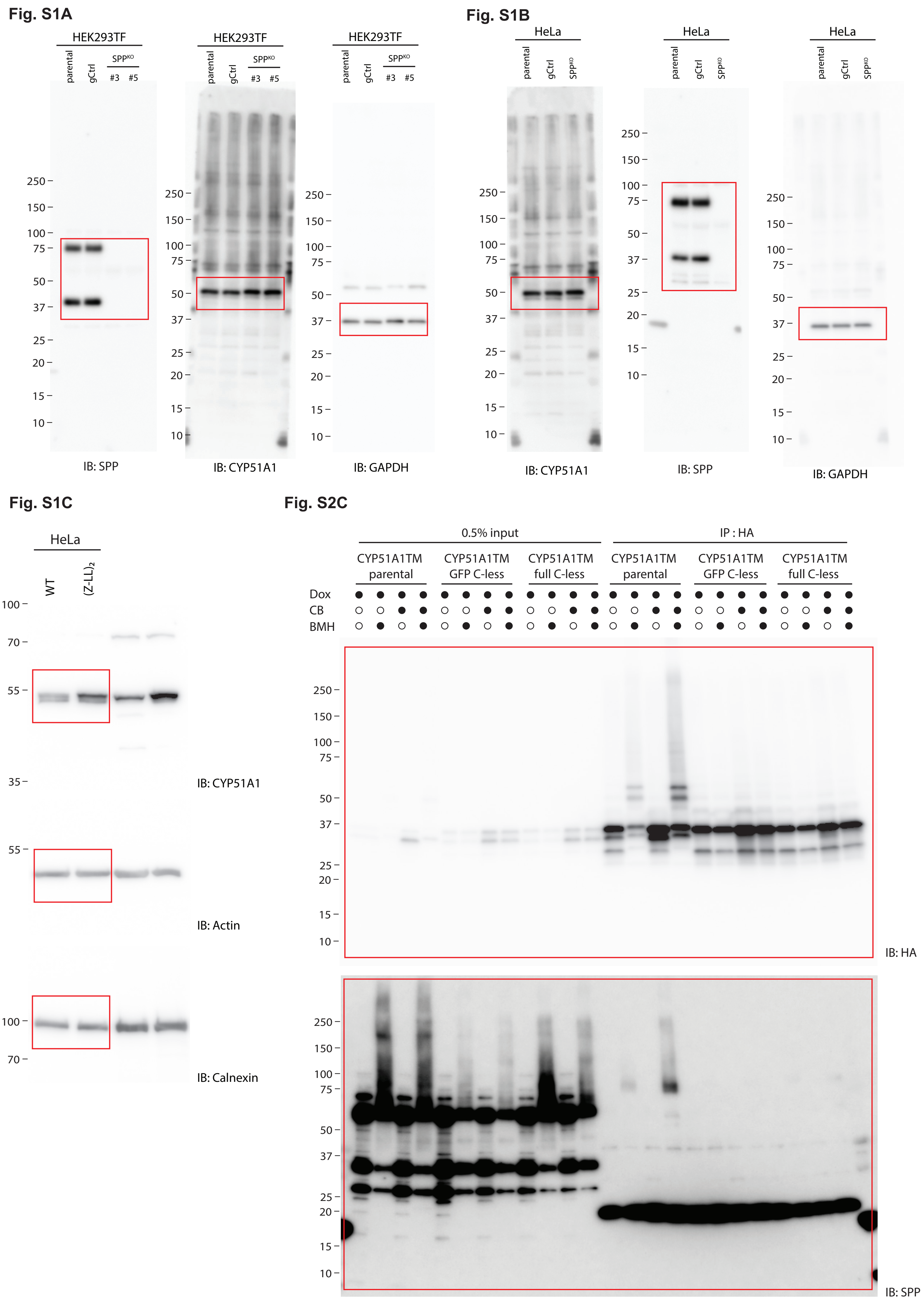

Fig. S18. Blot Transparency Figures - Full western blot images of Fig. S1A, S1B, S1C and S2C.

Journal of Cell Science • Supplementary information

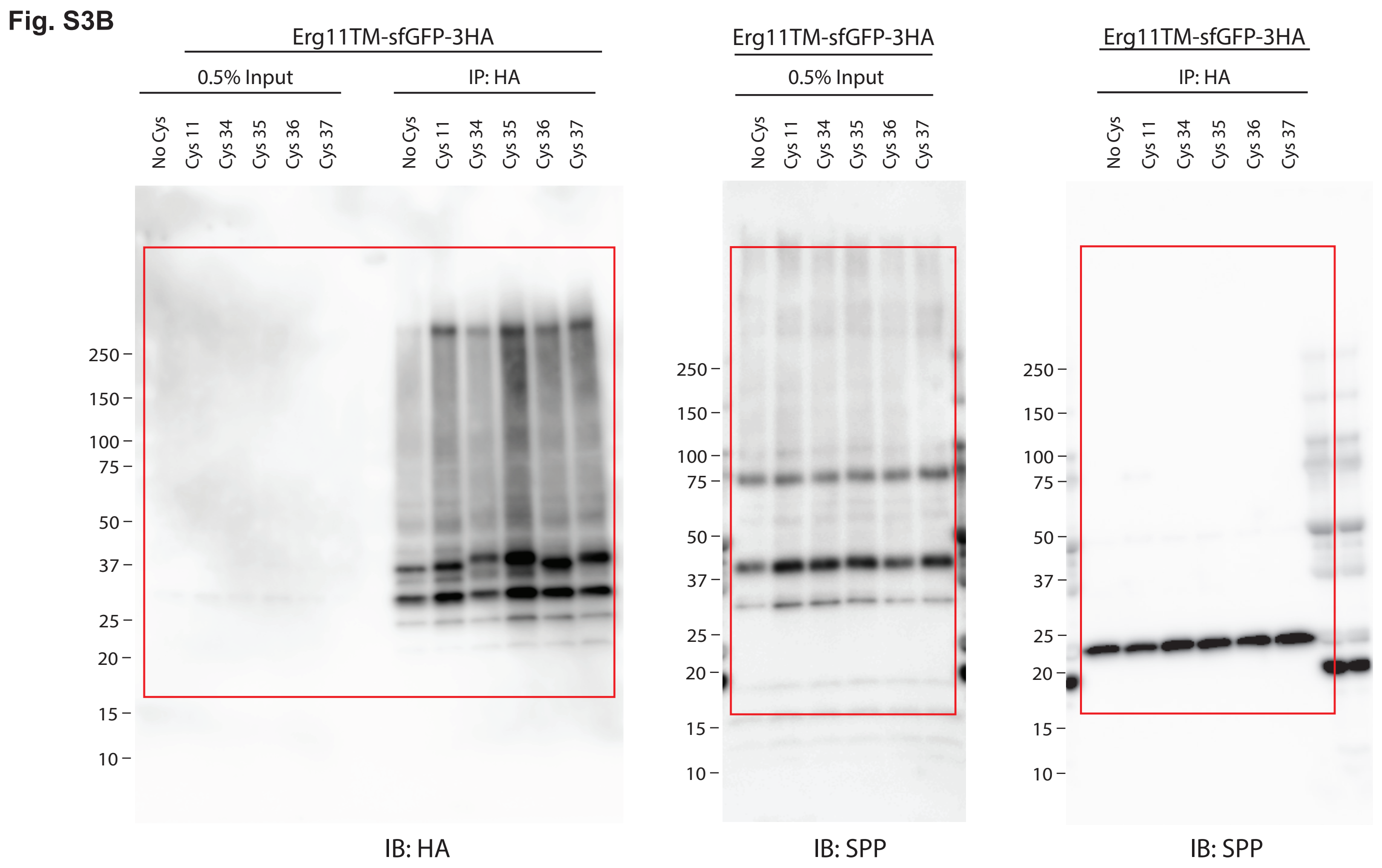

Fig. S19. Blot Transparency Figures - Full western blot images of Fig. S3B.

Journal of Cell Science • Supplementary information

Fig. S4

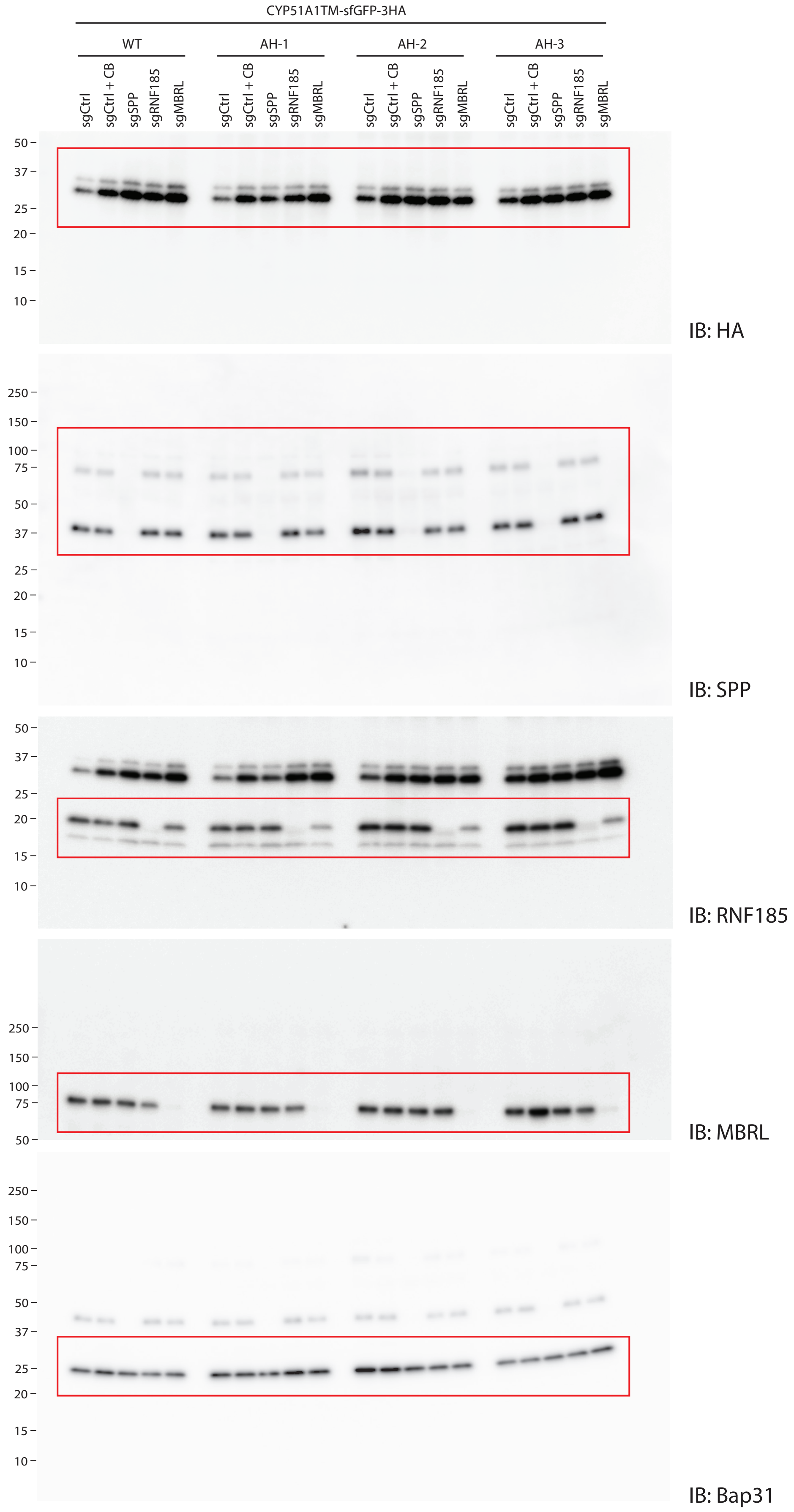

Fig. S20. Blot Transparency Figures - Full western blot images for Fig. S4.

Fig. S5F

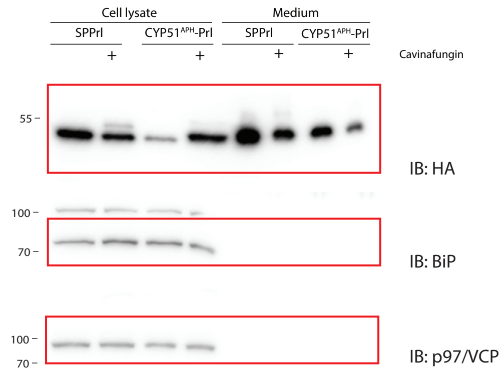

Fig. S7E

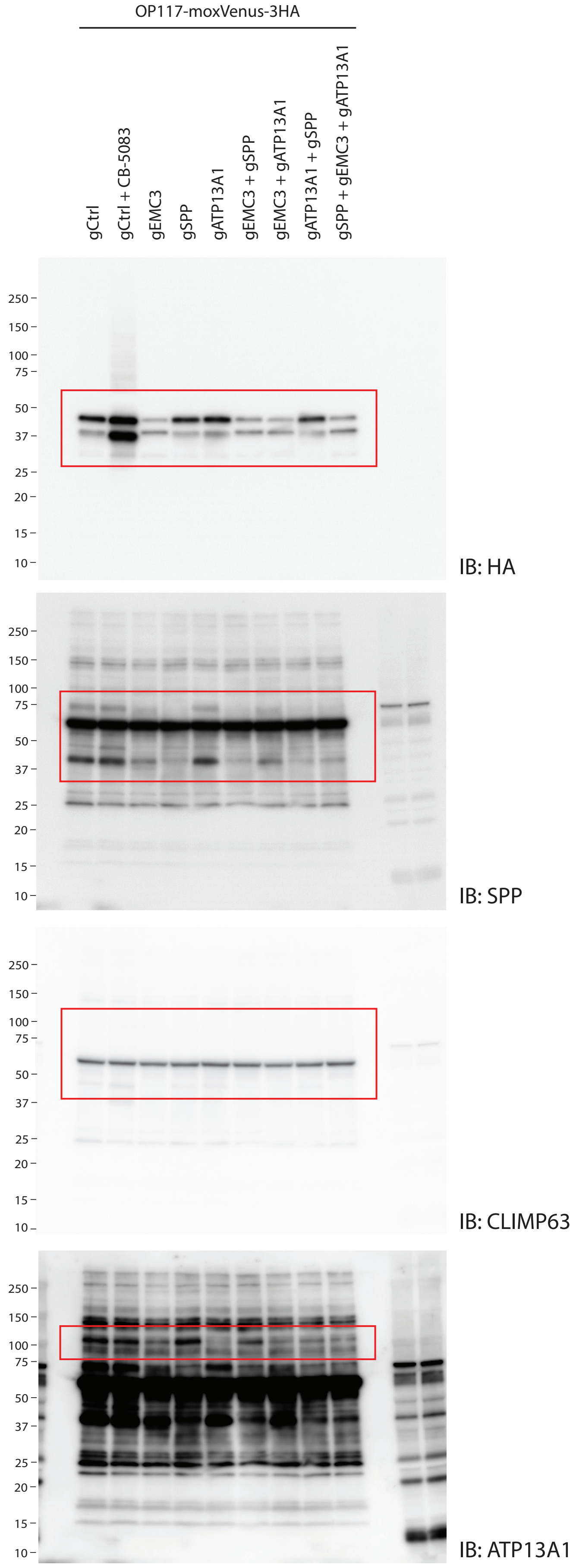

Fig. S21. Blot Transparency Figures - Full western blot images for Fig. S5F and S7E.

Journal of Cell Science • Supplementary information

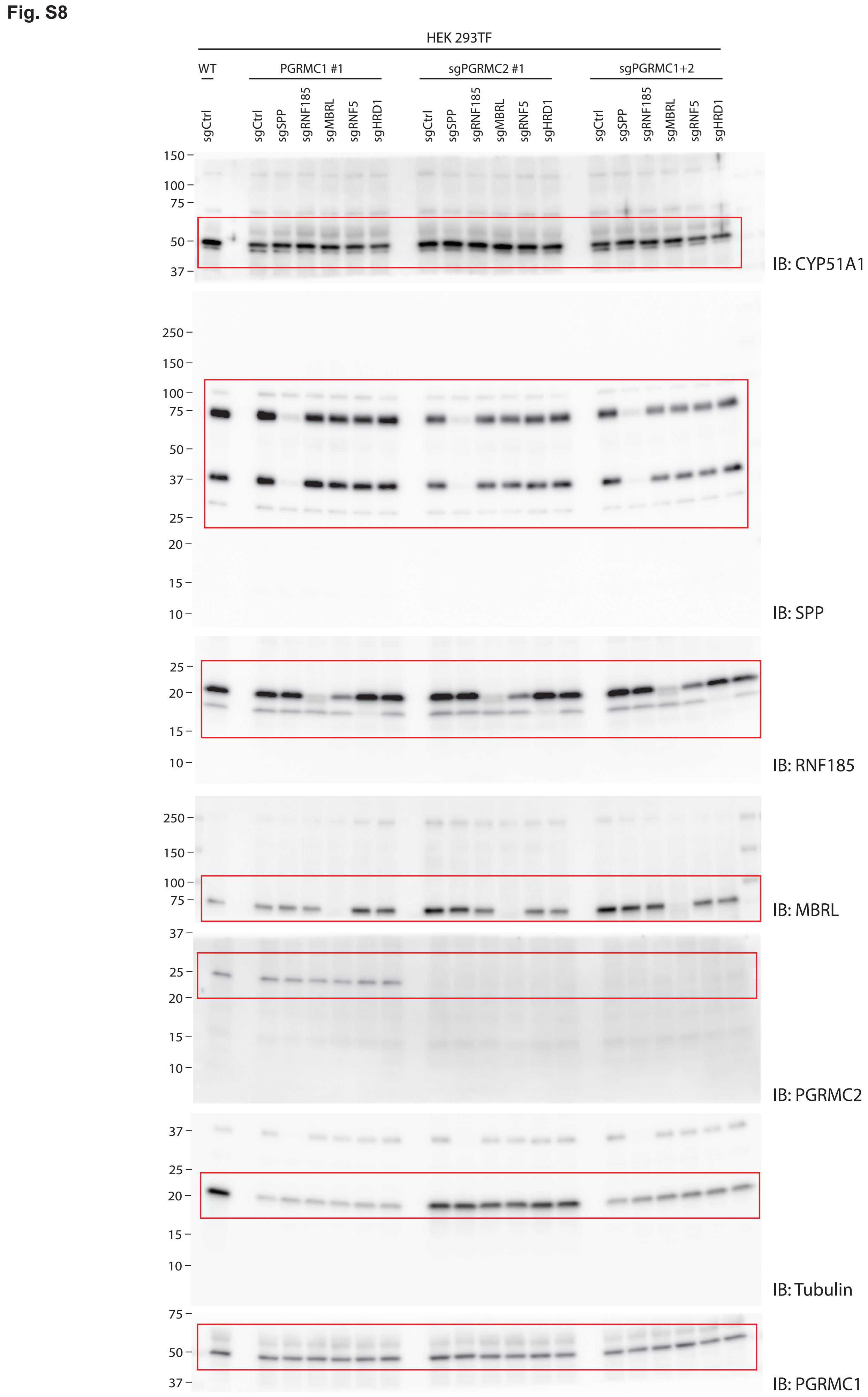

Fig. S22. Blot Transparency Figures - Full western blot images for Fig. S8.

Journal of Cell Science • Supplementary information
