# Supplementary material for: Topology surveillance of the lanosterol demethylase CYP51A1 by signal peptide peptidase
Source: J Cell Sci. 2024 Dec 12;137(23):jcs262333. doi: 10.1242/jcs.262333 (PMC11827857; doi:10.1242/jcs.262333)
Supplement: Supplementary file 1 [file joces-137-262333_review_history.pdf]

Journal of Cell Science

Topology surveillance of the lanosterol demethylase CYP51A1 by signal peptide peptidase

Nikita Sergejevs, Donem Avci, Michael L. van de Weijer, Robin A. Corey, Marius K. Lemberg and Pedro Carvalho
DOI: 10.1242/jcs.262333

Editor: Robert Parton

Review timeline

Original submission:	5 June 2024
Editorial decision:	22 July 2024
First revision received:	21 October 2024
Accepted:	4 November 2024

Original submission

First decision letter

MS ID#: JOCES/2024/262333

MS TITLE: Topology surveillance of the lanosterol demethylase CYP51A1 by Signal Peptide Peptidase

AUTHORS: Nikita Sergejevs, Donem Avci, Michael L van de Weijer, Robin A Corey, Marius K Lemberg, and Pedro Carvalho

We have now reached a decision on the above manuscript. As you will see the reviewers all felt that the manuscript made a significant contribution to the field and was potentially suitable for publication in the Journal of Cell Science. Reviewer 2 made a number of suggestions to improve the manuscript but did not feel that new experiments were absolutely essential. The other two reviewers raised some concerns that would need to be addressed in a revised manuscript.

I would be pleased to see a revised manuscript that addresses these concerns and I would then judge whether to return it to the reviewers.

To see the reviewers' reports and a copy of this decision letter, please go to: https://submit-jcs.biologists.org and click on the 'Manuscripts with Decisions' queue in the Author Area. (Corresponding author only has access to reviews.). The reviewers' comments are also included below.

Please ensure that you clearly highlight all changes made in the revised manuscript. Please avoid using 'Tracked changes' in Word files as these are lost in PDF conversion.

I should be grateful if you would also provide a point-by-point response detailing how you have dealt with the points raised by the reviewers in the 'Response to Reviewers' box. Please attend to all of the reviewers' comments. If you do not agree with any of their criticisms or suggestions please explain clearly why this is so.

Reviewer 1

Advance summary and potential significance to field

© 2024. Published by The Company of Biologists under the terms of the Creative Commons Attribution License (https://creativecommons.org/licenses/by/4.0/).

In this manuscript Sergejevs and colleagues report the role of signal peptide peptidase (SPP) in the quality control (ie topology surveillance) of CYP51A1. Using reporter constructs of CYP51A1, they show that SPP cleaves a fraction of CYP51A1 molecules and that the cleavage triggers ERAD-mediated clearance of CYP51A1. The authors identify a high selectivity of SPP for CYP51A1 molecules that contain an amphipathic helix inserted in the membrane with a type II orientation. This manuscript is of importance as it points towards the role of SPP in protein quality control in particular regarding topologically (and functionnally) impeded proteins. This information might be of particular relevance in specific genetic diseases altering topology of transmembrane proteins and documents another way for the cell to control (pre-emptively) ER homeostasis.

Comments for the author

The manuscript is well written and experimental results are of high quality. Conclusions drawn from the results are supported by the data. However several small issues should be addressed:

* Signal peptide peptidase activity is mediated by several proteins and it is not clear which component of SPP is targeted in the present manuscript, this should be indicated in a revised version

* Statistics should be performed on the graphs and the number of experimental repeats indicated in the figure legends

* for physiological relevance purposes it could be interesting to blunt SPP activity in cells naturally expressing high levels of CYP51A1 and evaluate its contribution to the regulation of CYP51A1 expression.

Reviewer 2

Advance summary and potential significance to field

This study provides evidence that CYP51A1 contains an N-terminal amphipathic helix (AH) that can inappropriately insert into the membrane in a type II (that is, N-cyt) topology, and that this topologic isoform is targeted for degradation. Degradation depends on cleavage of this AH by SPP, followed by ubiquitination by the RNF185/Membralin complex. The process seems to rather specific to CYP51A1 as none of ~20 other p450 enzymes or three mis-inserted GPCRs seem to be targets for SPP. The primary conceptual advance is that SPP can recognise and initiate the degradation of topologic errors in at least some proteins.

The data documenting the findings are generally convincing and the study is carefully interpreted throughout. I therefore support publication, and feel that additional experiments are not really necessary for the primary conclusions. I do however suggest two items that might benefit from some additional (straightforward) work, to be done at the authors' discretion, and a few minor points for clarification.

Comments for the author

Optional experiments:

1) Fig. 1D - the effect on endogenous CYP51A1 is rather modest, despite the fact that the exogenous protein is being overexpressed in those same cells (and hence, the total amount of CYP51A1-like proteins is actually being overexpressed). To make a more convincing case that endogenous CYP51A1 is also degraded by a SPP-dependent pathway, it seems better to monitor CYP51A1 in cells that are not expressing an exogenous substrate that could be saturating certain QC pathways. Have the authors verified that endogenous CYP51A1 levels increase in KO cells without exogenous CYP51A1TM overexpression?

© 2024. Published by The Company of Biologists under the terms of the Creative Commons Attribution License (https://creativecommons.org/licenses/by/4.0/).

1a) Related to point 1, it might be worth telling the reader how reliable the modest increase in endogenous CYP51A1 is (e.g., seen consistently in replicate experiments; perhaps an average with some measure of error could be useful).

2) I felt the most weakly supported conclusion is that the AH is in a type II topology. I am pretty sure the authors are correct, but that the experimental evidence is not very direct. The most direct way to show this would be to put a tag at the N-terminus and show that this tag is exposed to the cytosol (many ways one could do this). Tags appended to the N-terminus of a signal peptide do not affect the ability of the signal peptide to function (see PMID 26721998), so it might be that an N-terminally tagged CYP51A1TM would behave as the untagged protein. If it didn't, it might indicate that the AH inserts into the membrane from the lumen and the tag interferes with this. Regardless, it might be worth making such constructs and examining their behaviour.

Minor points:

1) In the crosslinking experiments, it is worth adding some labels for the major known products, including substrate, IgG light chain (presumably the major band in the IP samples blotted with SPP), and BMH-dependent products. Non-expert readers will otherwise have a hard time knowing what all the various bands are supposed to represent.

2) I wouldn't say "unique" in describing SPP's activity toward CYP51A1 since all type III proteins were not tested. Perhaps say SPP has "high selectivity" or something equivalent?

3) I don't feel the AF predictions really support the idea that the AH is dynamic. It seems more like the linker between the AF and TMD is flexible (which is not surprising given that there are two glycines in that linker). It seems more accurate to say that the AH does not make any confidently predicted interactions with the rest of the protein, arguing that its relative position is likely to be heterogeneous. Perhaps re-phrase?

4) I don't think the core N-liked glycan is "charged" as stated. Perhaps say "bulky hydrophilic glycan" instead.

5) The authors may wish to discuss why the AH seems to get cleaved by the signal peptidase complex (SPC) when fused to Prolactin but does not seem to get cleaved in its native CYP5A1 context.

Reviewer 3

Advance summary and potential significance to field

Nikita Sergejevs and coworkers describe in their manuscript a mechanism for Â‘Topology surveillance of the lanosterol demethylase CYP51A1 by Signal Peptide PeptidaseÂ’.
SPP appears to detect a specific membrane confirmation of an amphipathic helix of a CYP51A1 transmembrane domain model substrate, for proteolytic processing, prior to ERAD (RNF185-Membralin) mediated degradation. While the luminal amphipathic helix of CYP51A1 has the characteristics of a degron, it was apparently difficult to identify additional substrates of the SPP-RNF185-MBRL pathways, probably due to the high conformation / topology specificity. Regardless, the presented findings point to a novel role of for SPP in protein quality control, by topology surveillance coupled to ERAD of potentially non-functional conformers.

Comments for the author

To strengthen their conclusions, the authors should address the following minor and major points.

Major points:

1. Please show the localization of CYP51A1TM-sfGFP-3HA in different cells (WT, SPP, RNF185 and MBRL KO cells, as well as CB-5087 treated cells) using live cell microscopy or immunofluorescence.

© 2024. Published by The Company of Biologists under the terms of the Creative Commons Attribution License (https://creativecommons.org/licenses/by/4.0/).

2. Figure 1D. The steady state protein levels of endogenous CYP51A1 appeared to be regulated only marginally. Does turnover / half-life of the full length CYP51A1 depend on SPP, RNF185, MBRL? Can the SPP-RNF185-MBRL dependent turnover of CYP51A1 be modulated by drugs that alter the membrane composition (e.g. inhibitors of lanosterol synthesis?).

3. CYP51A1-TM can be crosslinked into a approx. 100kDa complex with an apparent SPP dimer. The SPP dimer is nicely visible on the blots, but the model substrate is sometimes hard to detect (e.g. Figure 3A, Figure 2G and others). Would it help to generate a CYP51A1-TM mutant in which only the cysteine in the membrane domain is mutated, to determine if cross-links occur exclusively within the GFP or within the TMD.

4. Figure 5 E, F: please do not quantify experiments with n=1. Please add additional biological samples for quantification.

5. To test the statement that CYP51A1-TM-G10N is indeed not recognized by SPP, could the authors test this assumption using a CO-IP experiment, or tone down the statement.

Minor points:

1. Please indicate how many cells were analyzed by FACS.
2. On some WB additional bands are visible for the model substrate, and for other proteins. Are all of these unspecific bands?
3. Please tone down the statement: '… SPP does not interfere with the general function of the RNF185/MBRL complex…' unless experimental evidence is provided.
4. Frequently, two bands for SPP are detected at 35 and 70kDa. Are these SDS resistant dimers?
5. Figure 2E: The 70kDa band for the SPP D265A appears to be reduced (?), please explain.
6. Figure 2F: please explain labelling: SPP (c-terminal)?
7. Line 239 '….during CYP51A1TM degradation (Figure 2F).' the wrong figure is called.
8. Line 254…do the authors really mean type III membrane protein or is this a typo? I would state a single pass type III transmembrane protein with a luminal n-terminus, but without a signal sequence.
9. In figure 5, it would be helpful to show a helical wheel of the AH and indicate the position of the artificial glycosylation site.
10. Figure S5 B,D – why are the colors of the TMDs swapped in Rank 1?
11. In some Figures (e.g. Figure S2A), there is a typo in the legend: 'incuced + p97 inhibitor'. Sometimes the authors use p97 inhibitor or CB-5087, please label the figures consistently.

First revision

Author response to reviewers' comments

Response to the reviewers

Reviewer #1

In this manuscript Sergejevs and colleagues report the role of signal peptide peptidase (SPP) in the quality control (ie topology surveillance) of CYP51A1. Using reporter constructs of CYP51A1, they show that SPP cleaves a fraction of CYP51A1 molecules and that the cleavage triggers ERAD-mediated clearance of CYP51A1. The authors identify a high selectivity of SPP for CYP51A1 molecules that contain an amphipathic helix inserted in the membrane with a type II orientation. This manuscript is of importance as it points towards the role of SPP in protein quality control in particular regarding topologically (and functionally) impeded proteins. This information might be of particular relevance in specific genetic diseases altering topology of transmembrane proteins and documents another way for the cell to control (pre-emptively) ER homeostasis.

© 2024. Published by The Company of Biologists under the terms of the Creative Commons Attribution License (https://creativecommons.org/licenses/by/4.0/).

The manuscript is well written and experimental results are of high quality. Conclusions drawn from the results are supported by the data. However several small issues should be addressed:

1- Signal peptide peptidase activity is mediated by several proteins and it is not clear which component of SPP is targeted in the present manuscript, this should be indicated in a revised version

We believe the reviewer is referring to the Signal Peptidase complex, an enzyme involved in the proteolytic cleavage of signal peptides. Our study focuses on a different enzyme: Signal Peptide Peptidase (SPP), an intramembrane aspartic protease that has received this name because among its substrates are signal peptides removed from proteins. In addition, SPP cleaves other substrates such as transmembrane domains of tail-anchored membrane proteins. SPP is not known to have any obligate binding partners to regulate its activity (PMID: 23585568, PMID: 37786993).

2- Statistics should be performed on the graphs and the number of experimental repeats indicated in the figure legends

We thank the reviewer for the suggestion. We have amended the figures and included the experimental repeats and quantifications, such as Fig. S1A, S1B.

3- for physiological relevance purposes it could be interesting to blunt SPP activity in cells naturally expressing high levels of CYP51A1 and evaluate its contribution to the regulation of CYP51A1 expression.

We have now analysed the impact of SPP ablation on the levels of endogenous CYP51A1 levels in two different cell lines, HEK293Trex Flip In and HeLa cells. In both cases we observe a small but significant increase in the steady state levels of CYP51A1 indicating that SPP contributes to maintain normal levels of this protein (Fig. S1A, S1B). These cell lines do not express CYP51A1TM or any other transgene, excluding the possibility of effects from exogenous proteins. A similar increase in the levels of endogenous CYP51A1 id observed when SPP activity is acutely inhibited by a selective inhibitor, Z-LL ketone (Fig. S1C). Altogether, these data show that SPP regulates the levels of endogenous CYP51A1.

Reviewer #2
Reviewer 2 Advance Summary and Potential Significance to Field...

This study provides evidence that CYP51A1 contains an N-terminal amphipathic helix (AH) that can inappropriately insert into the membrane in a type II (that is, N-cyt) topology, and that this topologic isoform is targeted for degradation. Degradation depends on cleavage of this AH by SPP, followed by ubiquitination by the RNF185/Membralin complex. The process seems to rather specific to CYP51A1 as none of ~20 other p450 enzymes or three mis-inserted GPCRs seem to be targets for SPP. The primary conceptual advance is that SPP can recognise and initiate the degradation of topologic errors in at least some proteins.

The data documenting the findings are generally convincing and the study is carefully interpreted throughout. I therefore support publication, and feel that additional experiments are not really necessary for the primary conclusions. I do however suggest two items that might benefit from some additional (straightforward) work, to be done at the authors' discretion, and a few minor points for clarification.

Reviewer 2 Comments for the Author...

Optional experiments:

1) Fig. 1D - the effect on endogenous CYP51A1 is rather modest, despite the fact that the

© 2024. Published by The Company of Biologists under the terms of the Creative Commons Attribution License (https://creativecommons.org/licenses/by/4.0/).

exogenous protein is being overexpressed in those same cells (and hence, the total amount of CYP51A1-like proteins is actually being overexpressed). To make a more convincing case that endogenous CYP51A1 is also degraded by a SPP-dependent pathway, it seems better to monitor CYP51A1 in cells that are not expressing an exogenous substrate that could be saturating certain QC pathways. Have the authors verified that endogenous CYP51A1 levels increase in KO cells without exogenous CYP51A1TM overexpression?

Please see response to Reviewer 1, point 3.

1a) Related to point 1, it might be worth telling the reader how reliable the modest increase in endogenous CYP51A1 is (e.g., seen consistently in replicate experiments; perhaps an average with some measure of error could be useful).

The increase in CYP51A1 levels upon SPP deletion/inhibition is reproducible across many biological replicates, observed on different SPP clones in HEK293Trex Flip In cells and multiple cell lines (HEK293Trex Flip In and HeLa) (Fig. S1A, S1B). We have now added a sentence in the text with this information.

2) I felt the most weakly supported conclusion is that the AH is in a type II topology. I am pretty sure the authors are correct, but that the experimental evidence is not very direct. The most direct way to show this would be to put a tag at the N-terminus and show that this tag is exposed to the cytosol (many ways one could do this). Tags appended to the N- terminus of a signal peptide do not affect the ability of the signal peptide to function (see PMID 26721998), so it might be that an N-terminally tagged CYP51A1TM would behave as the untagged protein. If it didn't, it might indicate that the AH inserts into the membrane from the lumen and the tag interferes with this. Regardless, it might be worth making such constructs and examining their behaviour.

We thank reviewer for this comment. We agreed that, in principle, the easiest way to monitor the dual topology of the AH would have been by adding a tag at the N- terminus of the protein. However, all attempts we made to introduce a N-terminal tag in resulted in its stabilization. We tried multiple epitope tags, with and without linker amino acids but invariably any modification blocked CYP51A1 recognition by SPP and degradation by ERAD. Some of these data is shown in Fig. S6.

As mentioned by the reviewer, we suspect that the addition of an N-terminal tag (normally composed of polar/charged) would destroy the metastable nature of the AH and “lock” it in the ER lumen, where it cannot be recognized by SPP and engage in ERAD.

Since an N-terminal tag was not an option, we resorted to the glycan acceptor sequence as shown in Figure 6. This strategy has the advantage of allowing the conditional block of glycosylation by the use of tunicamycin, as explained in the manuscript.

Minor points:

1) In the crosslinking experiments, it is worth adding some labels for the major known products, including substrate, IgG light chain (presumably the major band in the IP samples blotted with SPP), and BMH-dependent products. Non-expert readers will otherwise have a hard time knowing what all the various bands are supposed to represent.

We have now included the labels for the major known products of the crosslinking experiments, such as Figure 2G, Figure 3A, Fig. S2C, S3B.

2) I wouldn't say "unique" in describing SPP's activity toward CYP51A1 since all type III proteins were not tested. Perhaps say SPP has "high selectivity" or something equivalent?

We agree with the reviewer and have edited the statement.

3) I don't feel the AF predictions really support the idea that the AH is dynamic. It seems more

© 2024. Published by The Company of Biologists under the terms of the Creative Commons Attribution License (https://creativecommons.org/licenses/by/4.0/).

like the linker between the AF and TMD is flexible (which is not surprising given that there are two glycines in that linker). It seems more accurate to say that the AH does not make any confidently predicted interactions with the rest of the protein, arguing that its relative position is likely to be heterogeneous. Perhaps re-phrase?

We thank reviewer for this point. We have now rephrased the text.

4) I don't think the core N-liked glycan is "charged" as stated. Perhaps say "bulky hydrophilic glycan" instead.

We have changed the text accordingly.

5) The authors may wish to discuss why the AH seems to get cleaved by the signal peptidase complex (SPC) when fused to Prolactin but does not seem to get cleaved in its native CYP5A1 context

As mentioned in the main text, *in silico* prediction tool give a low score as an ER- targeting signal sequence for the CYP51A1 AH. A previous crystal structure of Erg11, the yeast homologue of CYP51A1 showed strong positional constrains between the AH, TMD and cytosolic catalytic domain (PMID: 24613931). When the AH is fused to Prolactin, those constrains are relieved and possibly facilitate it to adopt a type II topology and cleavage by SPC.

<u>Reviewer #3</u>

Reviewer 3 Advance Summary and Potential Significance to Field...

Nikita Sergejevs and coworkers describe in their manuscript a mechanism for ‘Topology surveillance of the lanosterol demethylase CYP51A1 by Signal Peptide Peptidase’. SPP appears to detect a specific membrane confirmation of an amphipathic helix of a CYP51A1 transmembrane domain model substrate, for proteolytic processing, prior to ERAD (RNF185-Membralin) mediated degradation. While the luminal amphipathic helix of CYP51A1 has the characteristics of a degron, it was apparently difficult to identify additional substrates of the SPP-RNF185-MBRL pathways, probably due to the high conformation / topology specificity. Regardless, the presented findings point to a novel role of for SPP in protein quality control, by topology surveillance coupled to ERAD of potentially non-functional conformers.

Reviewer 3 Comments for the Author...

To strengthen their conclusions, the authors should address the following minor and major points.

Major points:

1. Please show the localization of CYP51A1TM-sfGFP-3HA in different cells (WT, SPP, RNF185 and MBRL KO cells, as well as CB-5087 treated cells) using live cell microscopy or immunofluorescence.

We now show that CYP51A1TM localizes to the ER in parental, SPP KO, RNF185 and MBRL KO cells (Fig. S1D). Previously we showed that CYP51A1TM also localizes to the ER upon treatments with CB-5083 (p97 inhibitor) or Bortezomib (proteasome inhibitor) (PMID: 32738194).

2. Figure 1D. The steady state protein levels of endogenous CYP51A1 appeared to be regulated only marginally. Does turnover / half-life of the full length CYP51A1 depend on SPP, RNF185, MBRL? Can the SPP-RNF185-MBRL dependent turnover of CYP51A1 be modulated by drugs that alter the membrane composition (e.g. inhibitors of lanosterol synthesis?).

© 2024. Published by The Company of Biologists under the terms of the Creative Commons Attribution License (https://creativecommons.org/licenses/by/4.0/).

Please see the response to Reviewer #1 point 3 for the effect of SPP mutations on the levels of endogenous CYP51A1.

We analysed the turnover of endogenous CYP51A1 both using cycloheximide chase and S^{35} pulse-chase experiments. Both approaches indicate that most molecules of CYP51A1 are long lived.

We also tested if the levels of endogenous CYP51A1 changed upon sterol depletion. While we observed changes in control proteins (like HMGCR), the levels of endogenous CYP51A1 were largely unchanged (See Reviewers' Response Figure 1). Moreover, while addition of p97 inhibitor resulted in strong stabilization of HMGCR, it had only a marginal effect on CYP51A1. The data is included in this letter but we left it out of the manuscript since it does not add much information.

Considering all these results, we suspect that the increase in endogenous CYP51A1 levels in SPP, RNF185 and MBRL KO cells results from a small fraction of CYP51A1 molecules with the N-terminal helix in a type II topology becoming subject to quality control. There may be conditions favouring CYP51A1 to adopt this conformation but we could not identify them yet.

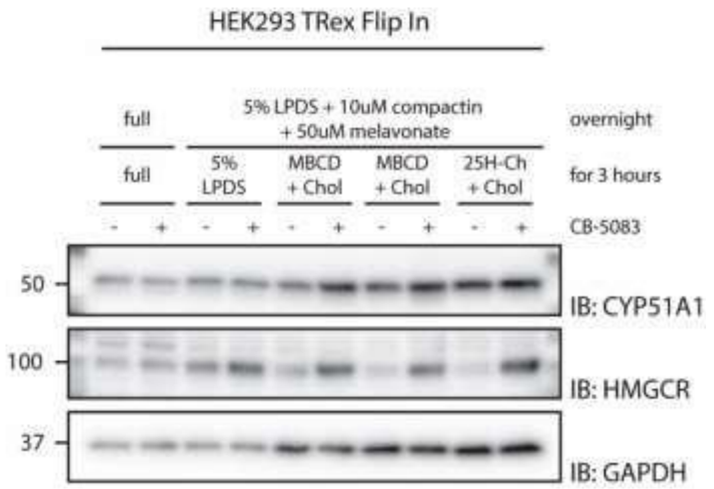

Reviewers' Response Figure 1. Steady state levels of endogenous CYP51A1 upon manipulation of cholesterol metabolism.

HEK293 cells were washed with PBS and cultured overnight in full or lipoprotein deficient medium (DMEM + 5% LPDS + 10 µM compactin +50 µM mevalonate + penicillin/streptomycin) before addition of 25-hydroxycholesterol (2 µg/ml) or MBCD-complexed cholesterol (20 µg/ml) for 3 hours in the absence or presence of the p97 inhibitor CB-5083 (2.5 µM) to analyse sterol-accelerated protein degradation. Protein levels of CYP51A1 and HMGCR were assessed by immunoblotting, GAPDH was included as a loading control.

3. CYP51A1-TM can be crosslinked into a approx. 100kDa complex with an apparent SPP dimer. The SPP dimer is nicely visible on the blots, but the model substrate is sometimes hard to detect (e.g. Figure 3A, Figure 2G and others). Would it help to generate a CYP51A1-TM mutant in which only the cysteine in the membrane domain is mutated, to determine if cross-links occur exclusively within the GFP or within the TMD.

We thank the reviewer for this suggestion. Our data indicate that the crosslinking to SPP occurs via cysteines within the GFP moiety of CYP51A1-TM (please see Fig. S2C). We observed that cysteine mutants within the GFP moiety of CYP51A1TM completely lost the ability to crosslink to endogenous SPP. This mutant protein contains a single cysteine in the

© 2024. Published by The Company of Biologists under the terms of the Creative Commons Attribution License (https://creativecommons.org/licenses/by/4.0/).

TMD region and behaves like its wild-type counterpart in all other assays we performed. Therefore, we concluded that the cysteine within the TMD does not contribute to the crosslinking to SPP.

The substrate crosslinks are hard to detect on the HA blot, because SPP- CYP51A1TM crosslinking bands represent a minor fraction of the total substrate crosslinks, hence, one way to see direct SPP-substrate crosslinks is by pulling down on the substrate (HA:IP) and then blotting for SPP (As in Figure 2G, 3A, Fig. S2C).

4. Figure 5 E, F: please do not quantify experiments with n=1. Please add additional biological samples for quantification.

We agree with the reviewer and have edited the figure to remove quantification of the experiment with n=1 (See Figure 6F, G).

5. To test the statement that CYP51A1-TM-G10N is indeed not recognized by SPP, could the authors test this assumption using a CO-IP experiment, or tone down the statement.

We agree with the reviewer and changed the text accordingly.

Minor points:

1. Please indicate how many cells were analyzed by FACS.

We have now included a statement in the Materials and Methods section specifying the number of cells used per condition analysed by FACS: “At least 10,000 cells per sample were analysed, gating on the main population in the forward scatter/side scatter (FSC/SSC) plot”.

2. On some WB additional bands are visible for the model substrate, and for other proteins. Are all of these unspecific bands?

Indeed, in some of the blots, the model substrates migrate as a doublet (Figure 1E, 1D, 2B, 2C, 2F). This behavior of the substrate has previously been observed in our laboratory (PMID: 32738194). We know that CYP51A1TM does not have glycosylation acceptor sequence, so the doublet cannot be attributed to N-linked glycosylation. We noticed that the presence of the doublet correlates with the lysis conditions (e.g lysis in Laemmli sample buffer vs milder DMNG detergent). In most experiments, the cells are lysed under non-denaturing conditions so it is likely that some degradation occurs in the lysate, even in presence of protease inhibitors. We have noticed that both bands are responsive to conditions analysed (e.g. Figure 1D, 1E, 2B) and importantly, both forms are still stably associated with the ER membrane, as shown previously (PMID: 32738194). In addition, the double bands were observed both in parental and the various KO cells analyzed (Figure 1D, 1E).

Additional lower molecular weight band in immunoblots looking at RNF185 (Figure 1D, 2F, 2C) represents RNF5, and the antibody is reported to recognise both proteins (RRID: AB_2922962).

For experiments involving BMH crosslinking, we have now included the labels for the major known products, such as Figure 2G, Figure 3A, Fig. S2C and Fig. S3B.

SPP is known to migrate as a monomer (~45kDa) and SDS-resistant dimer (~90 kDa), hence the presence of two species on the immunoblot (For example, Figure 1D, 2B, 2C, 2E and 2F) (PMID: 14704149) Doublet seen for TMUB1 represents two isoforms resulting from alternative start sites - long and short and has been seen previously (Figure 1D) (PMID: 32738194)

The lower molecular weight band on endogenous CYP51A1 blot represents a non- specific band and has been validated experimentally (Figure 1D) (See Reviewers’ Response Figure 2):

© 2024. Published by The Company of Biologists under the terms of the Creative Commons Attribution License (https://creativecommons.org/licenses/by/4.0/).

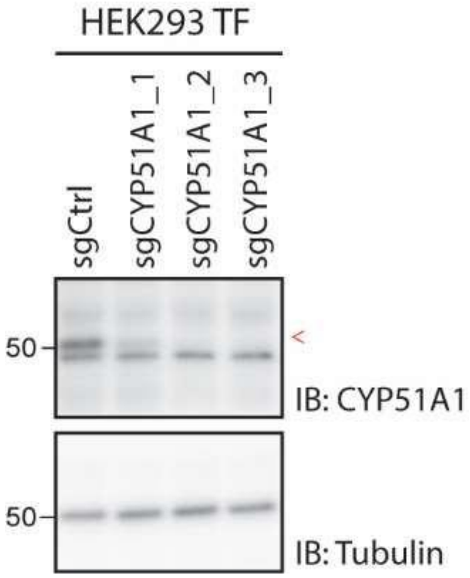

Reviewers' Response Figure 2. Validation of sgRNA's targeting endogenous CYP51A1. Top band represents CYP51A1 specific band and is marked with a red arrow.

3. Please tone down the statement: '… SPP does not interfere with the general function of the RNF185/MBRL complex…' unless experimental evidence is provided.

We show that depletion of SPP affects the turnover of CYP51A1TM but no other substrates of the RNF185/MBRL complex tested (such as TMUB2, CYP4A11TM, CYP26A1TM). Moreover, SPP does not appear to be part of the RNF185/MBRL complex (Figure 2C) and SPP KO cells have normal levels of both RNF185 and MBRL (Figure 1D). Together these data are a strong indication that depletion of SPP does not have a general effect on the function of the RNF185/MBRL complex.

4. Frequently, two bands for SPP are detected at 35 and 70kDa. Are these SDS resistant dimers?

Indeed, SPP forms SDS-resistant dimers on Western Blot, and it has been shown before in numerous studies (for example PMID: 14704149, PMID: 21636854).

5. Figure 2E: The 70kDa band for the SPP D265A appears to be reduced (?), please explain.

Possibly the SPP D265A mutant, which is catalytically inactive, folds less efficiently and may be less likely to form SDS-resistant dimers compared to WT SPP, as the amount of monomeric SPP is relatively similar (See Figure 2E, 2F).

6. Figure 2F: please explain labelling: SPP (c-terminal)?

The anti-SPP antibody recognizes an epitope at the very C-terminus of SPP. However, its ability to bind to SPP is strongly diminished when an epitope tag is fused to SPP C-terminus. Since that blot is unnecessary and may be a cause of confusion we decided to remove it from the Figure 2F.

7. Line 239 '….during CYP51A1TM degradation (Figure 2F).' the wrong figure is called.

We have amended the figure to refer to the right figure - Figure 2G.

8. Line 254…do the authors really mean type III membrane protein or is this a typo? I would state a single pass type III transmembrane protein with a luminal n-terminus, but without a signal sequence.

© 2024. Published by The Company of Biologists under the terms of the Creative Commons Attribution License (https://creativecommons.org/licenses/by/4.0/).

We have changed the text accordingly.

9. In figure 5, it would be helpful to show a helical wheel of the AH and indicate the position of the artificial glycosylation site.

We have now included an additional panel in Figure 6C with a helical wheel of the AH indicating the two positions of the artificial glycosylation site.

10. Figure S5 B,D - why are the colors of the TMDs swapped in Rank 1?

We have amended the figure to include the correct colour code for TMD in Rank 1 model (See Fig. S5B, D).

11. In some Figures (e.g. Figure S2A), there is a typo in the legend: 'incuced + p97 inhibitor'. Sometimes the authors use p97 inhibitor or CB-5087, please label the figures consistently.

We have amended the figures to remove typos (Fig. S3A) and labelled the figures consistently.

Second decision letter

MS ID#: JOCES/2024/262333

MS TITLE: Topology surveillance of the lanosterol demethylase CYP51A1 by Signal Peptide Peptidase

AUTHORS: Nikita Sergejevs, Donem Avci, Michael L van de Weijer, Robin A Corey, Marius K Lemberg, and Pedro Carvalho

I am happy to tell you that your manuscript has been accepted for publication in Journal of Cell Science, pending standard publication integrity checks.

Reviewer 1

Advance summary and potential significance to field

n/a

Comments for the author

no further comment on the manuscript, the authors addressed all the issues raised initially

Reviewer 2

Advance summary and potential significance to field

My few minor comments have been addressed and I support publication.

Comments for the author

My few minor comments have been addressed and I support publication.

Reviewer 3

Advance summary and potential significance to field

© 2024. Published by The Company of Biologists under the terms of the Creative Commons Attribution License (https://creativecommons.org/licenses/by/4.0/).

The manuscript describes how signal peptide peptidase (SSP) helps to perform topology surveillance of aberrantly membrane inserted CYP51A1 molecules at the ER. This fraction of CYP51A1 is cleaved by SSP for subsequent clearance by ERAD.

Comments for the author

The authors have addressed many of my original concerns and improved the manuscript. The manuscript provides interesting and well supported insight into membrane protein quality control and should be published.

© 2024. Published by The Company of Biologists under the terms of the Creative Commons Attribution License (https://creativecommons.org/licenses/by/4.0/).
